# An SP110–SP100 axis is a critical regulator of promyelocytic leukaemia body dynamics and mitotic fidelity

**DOI:** 10.1038/s41556-026-01916-w

**Published:** 2026-03-13

**Authors:** Eric J. Aird, Julius Rabl, Tabea Knuesel, Kevin Groen, Samah W. Awwad, Boris Korablev, Lynn Scherpe, Waleed Al-Herz, Robin Hupfer, Mike Recher, Stephen P. Jackson, Benjamin G. Hale, Jacob E. Corn

**Affiliations:** 1 Institute of Molecular Health Sciences, Department of Biology, ETH Zurich, Zurich, Switzerland; 2 Cryo-EM Knowledge Hub, ETH Zurich, Zurich, Switzerland; 3 Institute of Medical Virology, University of Zurich, Zurich, Switzerland; 4 Cancer Research UK Cambridge Institute, University of Cambridge, Cambridge, UK; 5 Department of Pediatrics, College of Medicine, Kuwait University, Kuwait City, Kuwait; 6 Immunodeficiency Laboratory, Department of Biomedicine, University of Basel, Basel, Switzerland; 7 University Center for Immunology, University Hospital, Basel, Switzerland

**Keywords:** Chromosome segregation, Functional genomics, PML bodies, Nuclear speckles, Cryoelectron microscopy

## Abstract

Stimulation of the innate immune system by foreign RNA elicits a potent interferon response and can trigger cell death. The mechanisms by which cells balance a robust response with cell-intrinsic lethality are still being uncovered. Here, using genome-wide CRISPR–Cas9 genetic screens with triphosphorylated RNA stimulation, we discover that promyelocytic leukaemia (PML) nuclear body-localized speckled protein 110 (SP110) is a potent inhibitor of type 1 interferon-driven cell death. Death suppression by SP110 counteracts a toxic activity of SP100, a major constituent of PML bodies. Loss of SP110 leads to mitotic retention of SP100 and PML bodies, which associate with and perturb segregating chromosomes, leading to micronucleus formation, DNA damage and genotoxic cell death. A combination of cryo-electron microscopy, AlphaFold modelling and cellular biochemistry reveals that SP110 dissolves toxic SP100 oligomers via necessary and sufficient direct interactions between their caspase activation and recruitment domains. These data reveal the critical roles of SP100 and SP110 in governing the disassembly of PML bodies during mitosis, as well as the repercussions if this process is misregulated.

## Abstract (web summary)

Aird et al. report that promyelocytic leukaemia (PML)-localized speckled protein 110 (SP110) controls SP100 oligomerization, which then regulates the mitotic dissolution of nuclear PML bodies.

## Main

The innate immune system guards against foreign pathogens and regulates cancer growth^1,2^. Both foreign and host molecules, including nucleic acids^3^, can stimulate an innate response and elicit the interferon signalling cascade leading to the upregulation of interferon-stimulated genes (ISGs). ISGs perform downstream functions including direct antipathogen activity^1^, stimulating the production of inflammatory cytokines^4^ and remodelling liquid–liquid phase-separated condensates to reinforce signalling cascades^5–7^. An estimated 10% of the human genome consists of ISGs, many of which are poorly characterized^8,9^.

Cell death is one intrinsic response to innate immune signalling, and hyperactivation or alterations to ISGs can disrupt the balance between sufficient innate immunity signalling and toxicity^10–12^. For example, insufficient amounts of cytosolic stress granule condensate proteins G3BP1/2 result in excessive inflammation and cell death in response to cytosolic DNA^13^. Precise mechanisms that balance the cellular response to innate immune stimulation with cell death are poorly described, especially in the context of large intracellular structures such as biomolecular condensates.

5′ triphosphorylated RNA (ppp-RNA) is a potent activator of ISG expression through recognition by diverse factors^14–16^, and ppp-RNA-induced interferon signalling leads to moderate amounts of intrinsic cell death^17,18^. Functional genomics has investigated genes that shape cellular fitness in response to various pathogens^19,20^. These studies yielded potential insights into interferon responses but were also confounded by factors such as viral entry and replication. A better understanding of the mechanisms that balance an appropriate cellular response to a molecularly defined innate immune stimulus with cell death could lead to novel biochemical insights and approaches to treating disease.

Using multiple genome-wide CRISPR inhibition (CRISPRi) screens with ppp-RNA as a stimulus, we discover that promyelocytic leukaemia (PML) body-localized speckled protein 110 (SP110) is a potent negative regulator of interferon signalling and downstream cell toxicity. This is mediated by an inhibitory direct interaction with PML body-localized SP100, whose interferon-induced upregulation is sufficient to trigger genotoxicity and cell death. Surprisingly, we find that balanced upregulation of SP110 in the face of interferon-induced SP100 expression is necessary for the normal mitotic dissolution of PML bodies. Failure to balance the SP110–SP100 axis leads to the accumulation of mitotically retained PML body/SP100 on segregating chromosomes, resulting in chromosome mis-segregation, micronucleus formation and lethal DNA damage. A cryo-electron microscopy (cryo-EM) structure of SP100 reveals that it forms homomeric oligomers, which are necessary and sufficient for cell death, via its caspase activation and recruitment domain (CARD). AlphaFold modelling and cellular biochemistry highlight the atomic details by which the necessary and sufficient CARD of SP110 disassembles the SP100 oligomers, enabling PML body dissolution. The balance of SP100 and SP110 expression is relevant during interferon signalling but also regulates appropriate PML body dissolution in non-immune contexts. Our findings together reveal a regulatory partnership between SP110 and SP100 that maintains cell health by appropriately remodelling biomolecular condensates.

## Results

### SP110 loss confers hypersensitivity to interferon stimulation

**Fig. 1:**
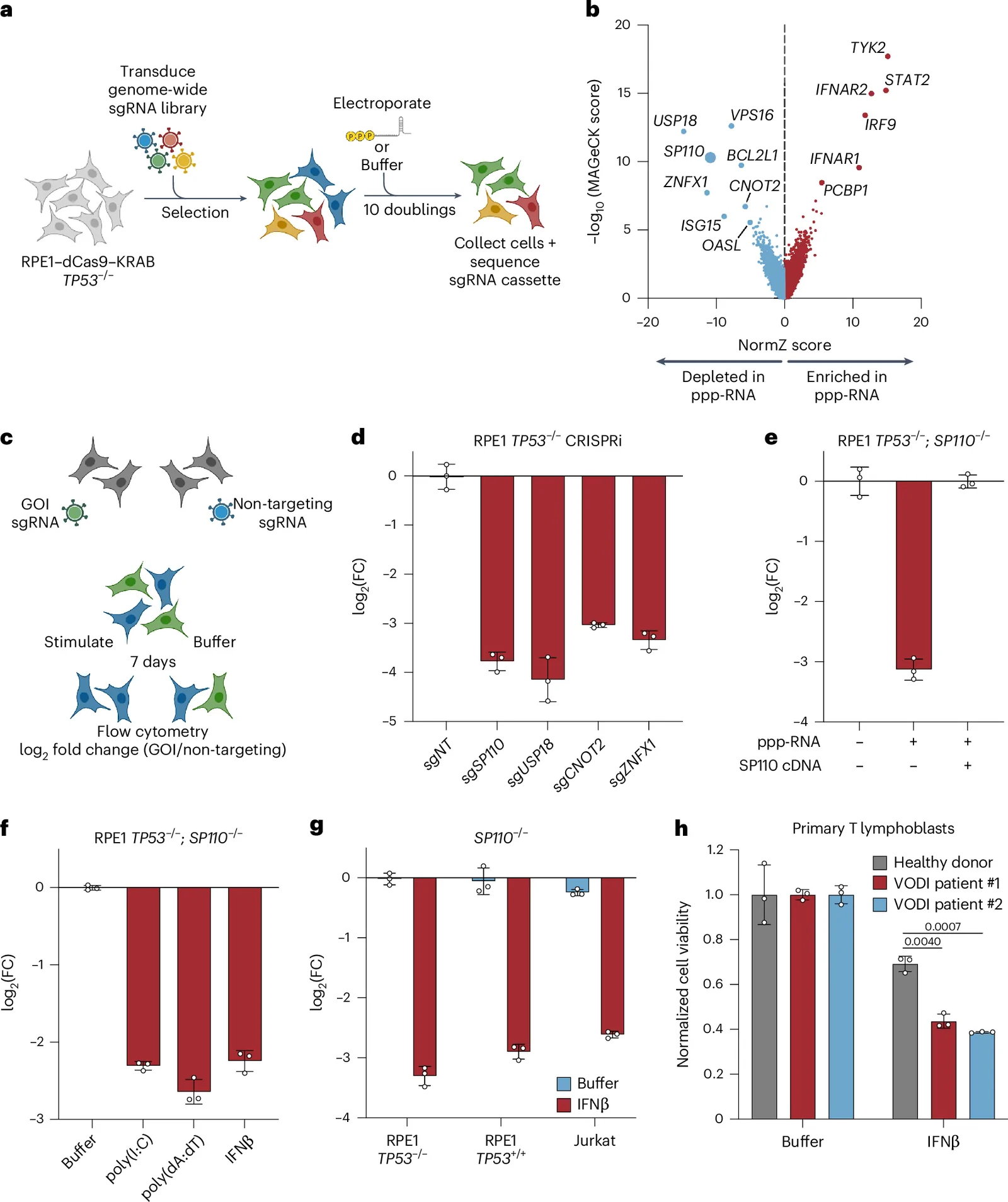
CRISPR screen reveals that SP110 protects against hypersensitivity to interferon stimulation. **a**, Schematic for the genome-wide CRISPRi screen in RPE1 WT (*TP53*^−/−^) cells. **b**, Gene-level scores comparing the MAGeCK score versus NormZ score. Each data point represents a gene. Genes left of *x* = 0 were depleted in the triphosphorylated (ppp-RNA)-treated condition. **c**, Schematic of the colorimetric cell competition assay format used throughout the study. GOI, gene of interest. **d**, Competition assay to individually validate candidate genes identified in the screen. All samples were treated with ppp-RNA. Data are plotted as the log_2_ value of the abundance of the indicated genotype relative to WT cells on day 7 versus day 0. sgNT, non-targeting sgRNA; FC, fold change. **e**, Rescue experiment in *SP110*-KO cells with full-length SP110 cDNA introduced via lentiviral overexpression. **f**, Competition assay comparing WT with *SP110*-KO cells treated with the indicated innate immune stimulators. **g**, Competition assay in the indicated cell types in an *SP110*-KO background either mock-treated (Buffer) or treated with recombinant IFNβ. **h**, Cell viability assay (using ATP abundance as a proxy) in mock- (Buffer) or recombinant IFNβ-treated primary patient-derived T lymphoblasts 6 days post-treatment. Statistical significance was determined with a two-tailed Welch’s *t*-test. Each data point in **d**–**h** represents an independent biological replicate (*n* = 3). Bar graphs are displayed as mean ± s.d. Schematics in **a** and **c** created in BioRender: Corn, J. https://biorender.com/hcz7tbl (2026). Source data

Our laboratory and others have reported that electroporation of triphosphorylated guide RNAs used in CRISPR genome editing elicits a remarkably strong interferon response^14,15^. Using a triphosphorylated guide RNA as a molecularly defined model for potent ppp-RNA stimulation, we sought to identify factors that regulate the balance between ISG signalling and cell death. We used a genome-wide CRISPRi screen to individually knock down (KD) the expression of each human gene and assess the impact on cellular fitness upon ppp-RNA treatment (Fig. 1a). The screen was performed in hTERT-RPE1 *TP53*^−/−^ cells, a non-cancerous and karyotypically normal cell line that has a robust interferon activation in response to foreign RNA^21^.

**Extended Data Fig. 1:**
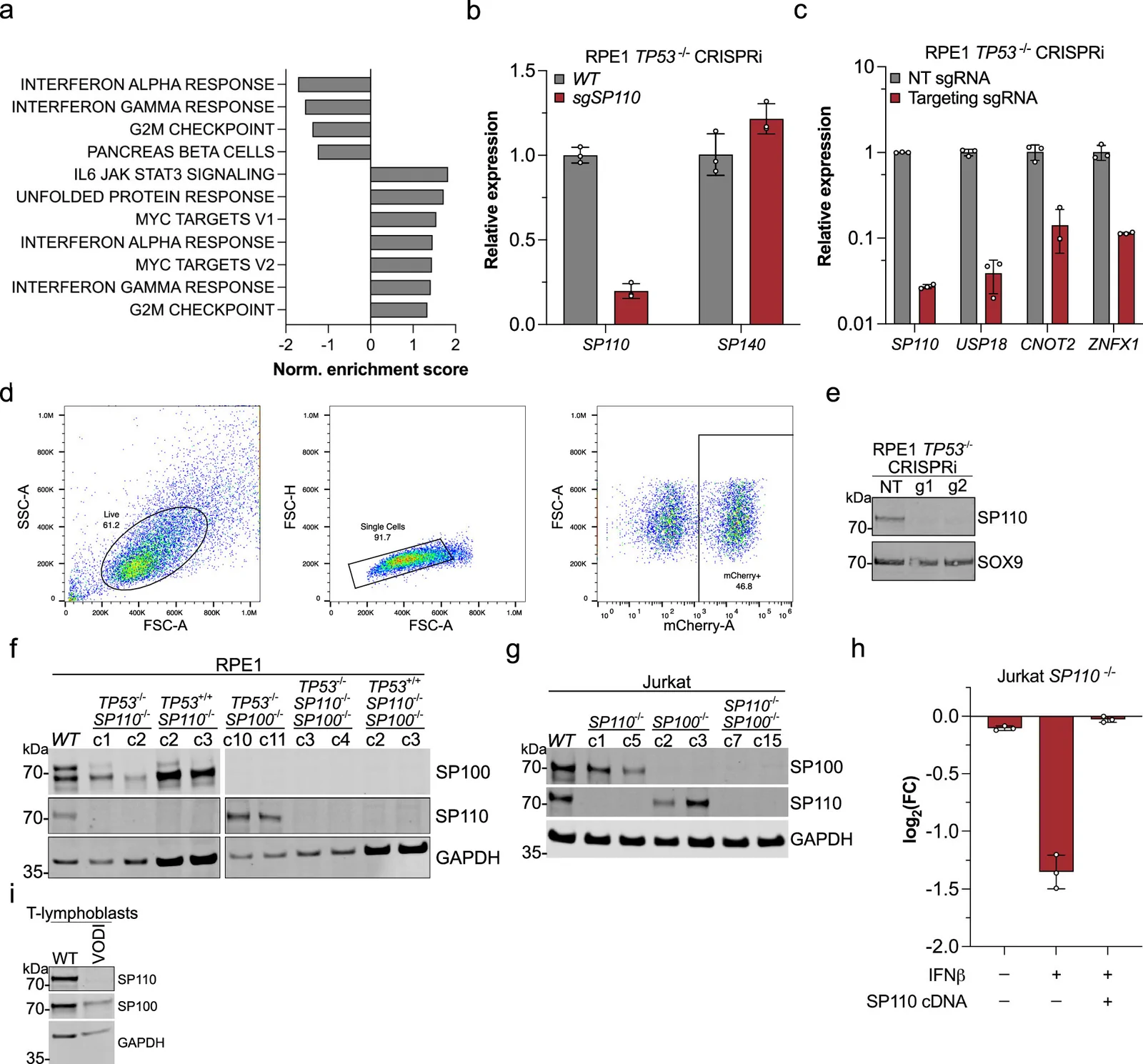
Validation of SP110 depletion. **a**. Gene Set Enrichment Analysis (GSEA) from the genome-wide screen from either the genes depleted (negative normalized enrichment score) or enriched (positive normalized enrichment score) in the ppp-RNA treated condition classified according to the Molecular Signatures Database (MSigDB) hallmark gene lists with a nominative *p*-value < 0.05 (sample permutation test). **b**. Validation of *SP110* specific knockdown by CRISPRi at the RNA level. Data points are technical replicates. Data is representative of *n* = 2 independent biological replicates. **c**. Validation of specific gene knockdown by CRISPRi from Fig. 1d. Data points are technical replicates. Data is representative of *n* = 2 independent biological replicates. **d**. Representative flow cytometry gating strategy. Live cells are gated on SSC-A versus FSC-A. Single cells are gated on FSC-H versus FSC-A. Fluorescent positive cells are gated on FSC-A versus fluorescent protein (mCherry, GFP, or BFP). **e**. Validation of SP110 knockdown at the protein level in the screening cells. NT = non-targeting guide RNA. **f** and **g**. Validation of SP110 specific knockout of selected clones in indicated cell backgrounds. c1 = clone 1. **h**. Competition assay in Jurkat *SP110*-KO cells ± IFNβ treatment and ± SP110 stable overexpression. Data points represent *n* = 3 individual biological replicates. **i**. Western blot assessing SP110 and SP100 expression in primary patient-derived T-lymphoblasts. WT = healthy donor. VODI = VODI patient #1. Bars in **b**, **c** and **h** denote mean ± s.d. Western blots in **e**, **f**, **g** and **i** were performed 2–3 independent times with similar results. Source numerical data and unprocessed blots are available in source data. Source data

A combination of statistical metrics (Methods) identified hundreds of genes that were significantly enriched or depleted in the ppp-RNA-treated condition relative to mock-treated cells (Fig. 1b, Supplementary Fig. 1 and Supplementary Data 1 and 2). Among these were several genes known to be essential for type 1 interferon signalling such as *IFNAR1*/*2, STAT2* and *TYK2*. Guide RNAs targeting these genes were enriched in the ppp-RNA-treated cells, presumably due to an inability to propagate an interferon response leading to a selective fitness advantage. RNA sensors of the DExD-box helicase (DDX) family such as *RIGI* (*DDX58*) and *DDX56*^22^ were also enriched in treated cells, with the lower effect sizes reflecting their known redundancy in recognizing ppp-RNA. Conversely, negative regulators of interferon signalling such as *USP18* and *ISG15* were significantly depleted in the treated condition. Gene set enrichment analysis (GSEA) reinforced that the screen accurately identified factors involved in innate immune signalling (Extended Data Fig. 1a).

Among the top depleted genes was *SP110*, one of four human speckled proteins (SPs) localized to phase-separated nuclear PML bodies that are generally linked to modulating the response to pathogen infections and regulating chromatin structure^23–25^. We individually validated the top depleted genes from the screen using a growth competition assay (Fig. 1c) and confirmed that *SP110* KD rendered cells extraordinarily hypersensitive to ppp-RNA treatment, to a similar extent as known negative regulators of the interferon response such as *USP18* (Fig. 1d and Extended Data Fig. 1b–d). We also generated isogenic *SP110*-knockout (KO) clones and verified that these phenocopy CRISPRi KD (Extended Data Fig. 1e). Innate immune hypersensitivity of *SP110*-KO cells was rescued through complementation of full-length SP110 via lentiviral overexpression (Fig. 1e), confirming the observed lethality is specific to loss of SP110.

We tested whether SP110 is directly involved in single-strand RNA sensing but instead found that bypassing single-strand RNA recognition with poly(I:C), poly(dA:dT) or recombinant interferon β (IFNβ) addition also induced cell death in *SP110*-KO cells (Fig. 1f). We established that IFNβ-mediated cell death is independent of *TP53* status and relevant in multiple cell types, as RPE1 *SP110*^−/−^; *TP53*^+/+^ cells and Jurkat *SP110*^−/−^ cells were hypersensitized to death caused by interferon stimulation (Fig. 1g and Extended Data Fig. 1f–h). In addition, primary T lymphoblasts isolated from *SP110*-deficient patients (*SP110* c.642delC) with veno-occlusive disease with immunodeficiency (VODI)^26^ exhibited augmented interferon-stimulated cell death relative to a healthy donor control (Fig. 1h and Extended Data Fig. 1i). Thus, SP110 is a potent negative regulator of interferon-induced cell death that acts downstream of ppp-RNA sensing. The strength of this effect and the unclear mechanistic basis led us to further investigate how SP110 protects against cell death.

### SP110 loss drives SP100 interferon susceptibility

**Fig. 2:**
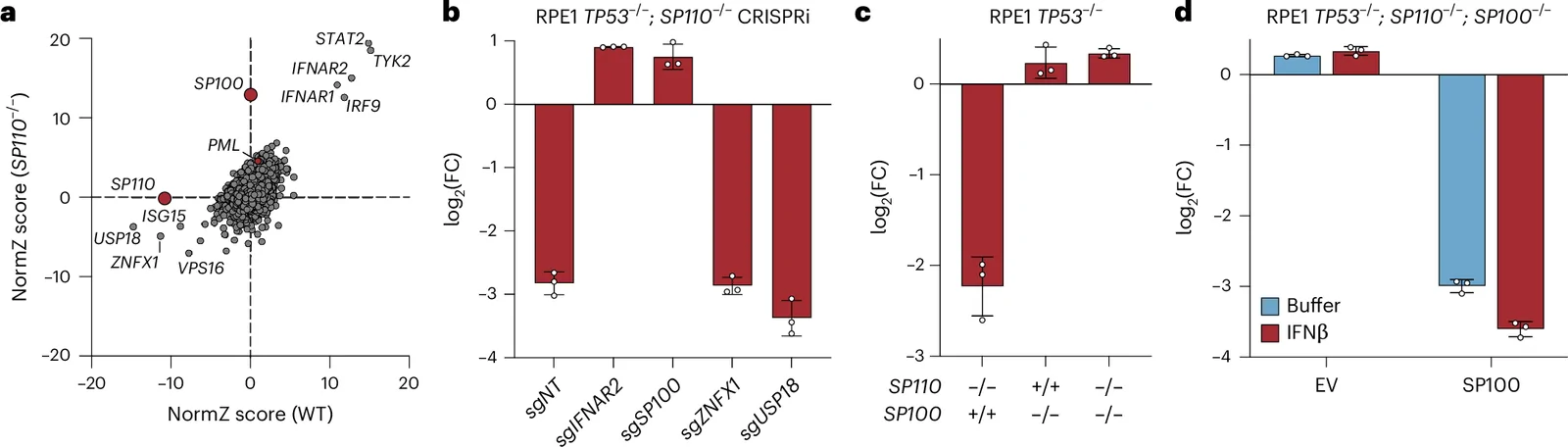
SP100 induces cellular toxicity in the absence of SP110. **a**, Comparison of gene-level NormZ scores from genome-wide screens in *SP110*-KO versus WT (*TP53*^−/−^) cells. **b**, Competition assay to validate candidate genes from the CRISPRi screen in *SP110*-KO cells comparing IFNβ treatment versus buffer. **c**, Competition assay in the indicated genotype relative to WT cells treated with IFNβ. **d**, Rescue experiment in *SP110*;*SP100* dual-KO cells with lentiviral overexpressed empty vector (EV) or SP100 treated with the indicated conditions. Each data point in **b**–**d** represents an independent biological replicate (*n* = 3). Bar graphs are displayed as mean ± s.d. Source data

**Extended Data Fig. 2:**
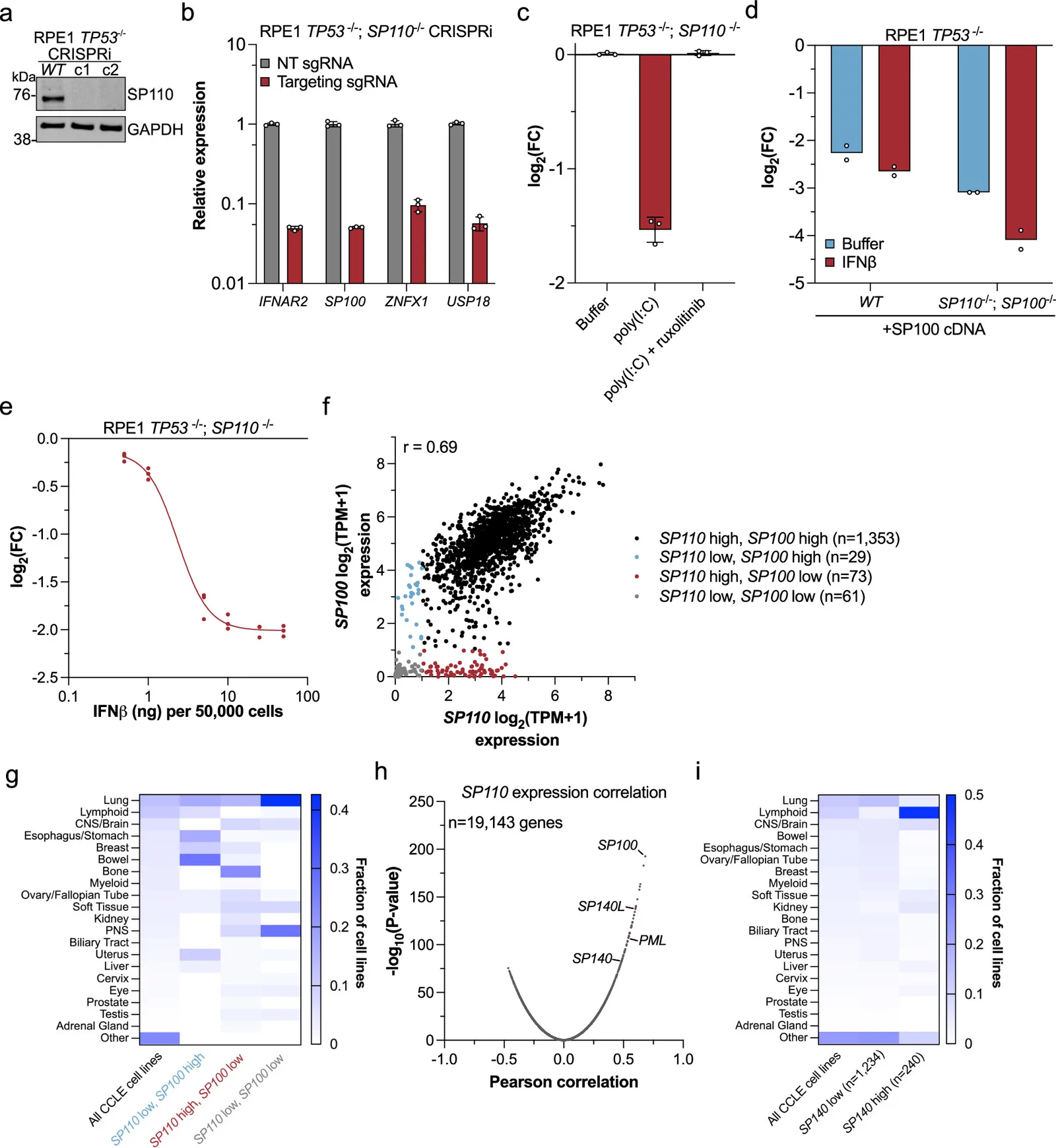
Elucidating the SP110-SP100 relationship. **a**. Confirmation of *SP110*-KO clones in the RPE1 *TP53*^*−/−*^ CRISPRi background. **b**. Validation of CRISPRi from genes assayed in Fig. 2b. Data points are technical replicates. Data is representative of *n* = 2 independent biological replicates. **c**. Competition assay comparing the ratio of *SP110*-KO versus WT cells on day 7 with the indicated treatments. **d**. Competition assay in the indicated genotypes stably expressing mCherry-SP100. Bars denote mean. **e**. Competition assay comparing *SP110*-KO versus WT cells with titration of IFNβ. Line denotes sigmoidal 4 parameter logistic curve. **f**. *SP100* versus *SP110* expression across all cell lines in the Cancer Cell Line Encyclopedia (CCLE) (*n* = 1474 cell lines) binned by low (log_2_ < 1 TPM) or high (log_2_ > 1 TPM) expressors. r = Pearson correlation coefficient. **g**. Heat map of fraction of tissue origin for each category of cell lines binned in **f**. **h**. Comparison of Pearson correlation coefficients between expression of *SP110* and every gene (*n* = 19,143) in the CCLE. **i**. Heat map of *SP140* expression in all CCLE cell lines grouped by low (log_2_ < 2 TPM) or high (log_2_ > 2 TPM) expression levels. Bars in **b** and **c** denote mean ± s.d. Data points in **c**-**e** represent individual biological replicates (*n* = 2–3). Source numerical data and unprocessed blots are available in source data. Source data

To discover which protein(s) or pathway(s) promote interferon-stimulated toxicity upon SP110 loss, we performed a synthetic viability CRISPRi screen in RPE1 *SP110*^−/−^;*TP53*^−/−^ cells (Extended Data Fig. 2a, Supplementary Fig. 1 and Supplementary Data 1 and 2). As in *TP53*^−/−^ RPE1 cells (indicated throughout as WT), *STAT2* and *TYK2* KD desensitized *SP110*-KO cells to ppp-RNA treatment, consistent with an SP110-independent effect (Fig. 2a). However, we found that KD of *SP100* was strongly protective against ppp-RNA treatment in *SP110*-KO cells without conferring a growth advantage to WT cells (Fig. 2a). SP100 is a major constituent of SP110-containing PML bodies and, like SP110, is induced by interferon signalling^23^. We also noticed that KD of *PML* partially rescued the IFNβ hypersensitivity of *SP110*-KO cells (Fig. 2a). Competition assays with individual KDs validated that positive-scoring genes (*IFNAR2* and *SP100*) rescued toxicity of stimulated *SP110*-KO cells to varying degrees and negative-scoring genes (*USP18*) exacerbated toxicity (Fig. 2b and Extended Data Fig. 2b). Chemical inhibition of interferon signalling using ruxolitinib also prevented cell death in *SP110*-KO cells (Extended Data Fig. 2c).

We further confirmed that SP100 loss rescues SP110-induced interferon-mediated cell death through the generation of *SP110;SP100* dual-KO cells (Fig. 2c and Extended Data Fig. 1e). We then performed rescue experiments in this dual-KO background. Reintroduction of SP100 was sufficient to recapitulate interferon-induced cell death (Fig. 2d). Surprisingly, we also found that overexpression of SP100 was sufficient to induce cell death in the absence of IFNβ stimulation. Even in *SP110*-proficient cells, overexpression of SP100 induced lethality (Extended Data Fig. 2d). The magnitude of cell death in WT cells was lower than in *SP110;SP100* dual-KO cells, implying that the balance between SP100 and SP110 expression is linked to the degree of toxicity. We explored this by titrating IFNβ in *SP110*-KO cells and observed dose-dependent toxicity (Extended Data Fig. 2e). We furthermore found that the basal expression of *SP110* and *SP100* are highly correlated (*r* = 0.69) across 1,474 cell lines in the Cancer Cell Line Encyclopedia (CCLE)^27^ (Extended Data Fig. 2f,g). Indeed, the *SP100* expression shows a stronger correlation with SP110 across all CCLE lines than with any other transcript (Extended Data Fig. 2h). SP110 and SP100 are broadly expressed across cell and tissue types in contrast to another SP, SP140, whose expression is largely lymphoid restricted (Extended Data Fig. 2i). Because SP110 and SP100 are both co-induced by interferon stimulation, our data suggest that SP110 levels are regulated to prevent cell death resulting from the toxic activity of SP100.

### SP100 toxicity is not dependent on transcriptional or viral restriction functions

**Extended Data Fig. 3:**
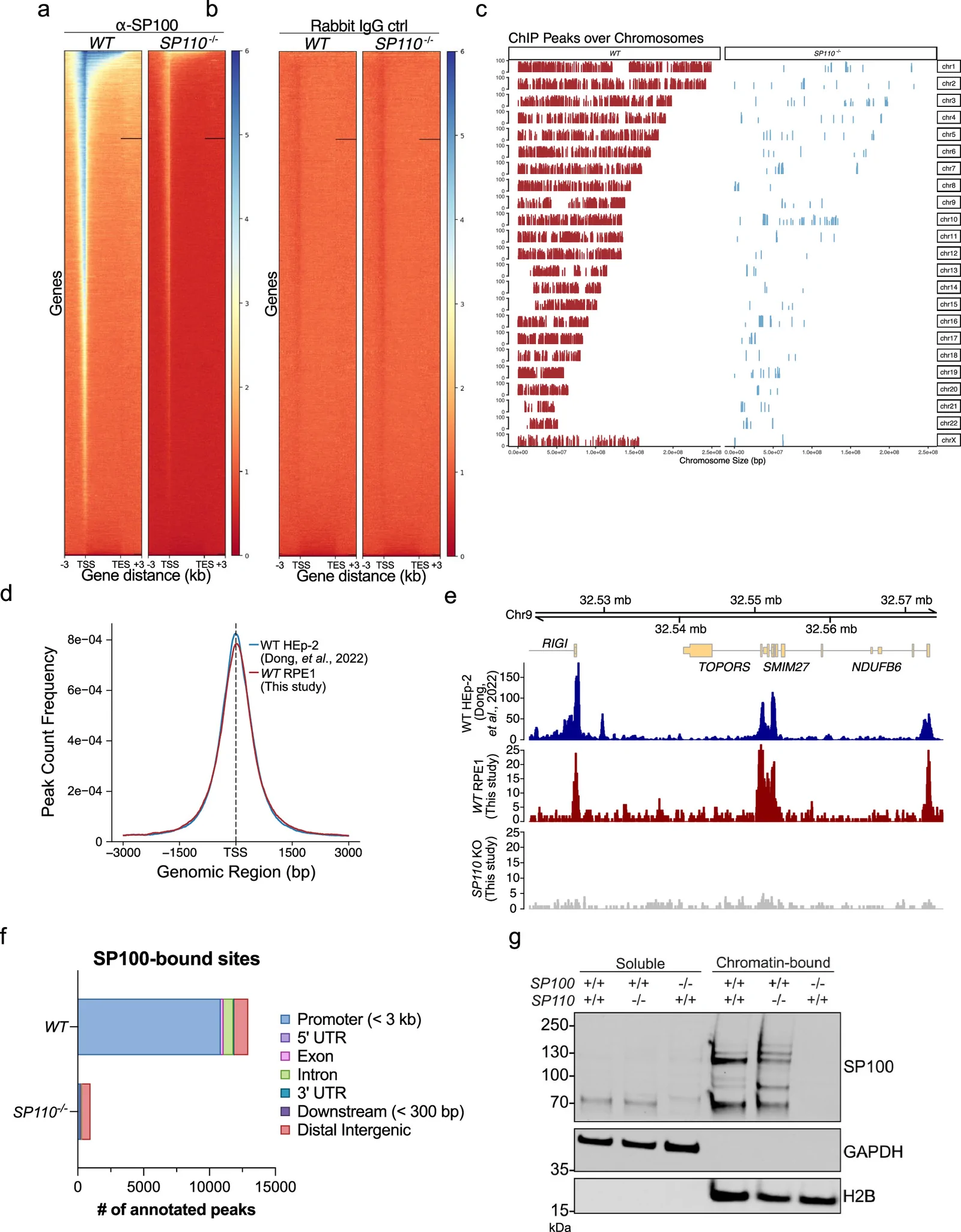
SP100 chromatin binding characteristics. **a**. Heat map of endogenous SP100 ChIP-Seq peaks in and surrounding gene bodies of normalized length. Each line corresponds to a gene. The color scale indicates the relative intensity of the signal. **b**. Heat map of rabbit IgG isotype control ChIP-Seq peaks in and surrounding gene bodies of normalized length. Each line corresponds to a gene. The color scale indicates the relative intensity of the signal. **c**. SP100 peak annotations based on chromosome location. **d**. Comparison of SP100 peak localization relative to the transcription start site (TSS) in stimulated RPE1 cells compared to HEp-2 cells^28^. **e**. Comparison of SP100 binding in HEp-2 cells^28^, WT RPE1 cells and *SP110*-KO RPE1 cells at an example locus. The *y*-axis corresponds to read depth. **f**. SP100 peak annotations based on location within gene body in RPE1 cells. **g**. Western blot assessing endogenous SP100 localization in soluble or chromatin-bound fractions. The soluble fraction is marked by GAPDH and the chromatin-bound fraction by histone H2B. Experiment was performed 2 independent times with similar results. Source numerical data and unprocessed blots are available in source data. Source data

SP110 and SP100 are both frequently associated with chromatin function^23^, so we assessed the genomic localization of endogenous SP100 in WT and *SP110*-KO cells. Chromatin immunoprecipitation followed by sequencing (ChIP-seq) confirmed that SP100 is predominately localized to promoters in WT cells as previously characterized^28^ (Extended Data Fig. 3a–e). However, in *SP110*-KO cells, SP100 no longer showed specific enrichment at promoters and instead displayed a diffuse ChIP-seq signal (Extended Data Fig. 3a,c,e,f). SP100 still preserved overall chromatin association in *SP110*-KO cells as in WT (Extended Data Fig. 3g). These data demonstrated that SP100’s precise chromatin positioning depends upon SP110, but its DNA binding capacity is independent of SP110.

**Extended Data Fig. 4:**
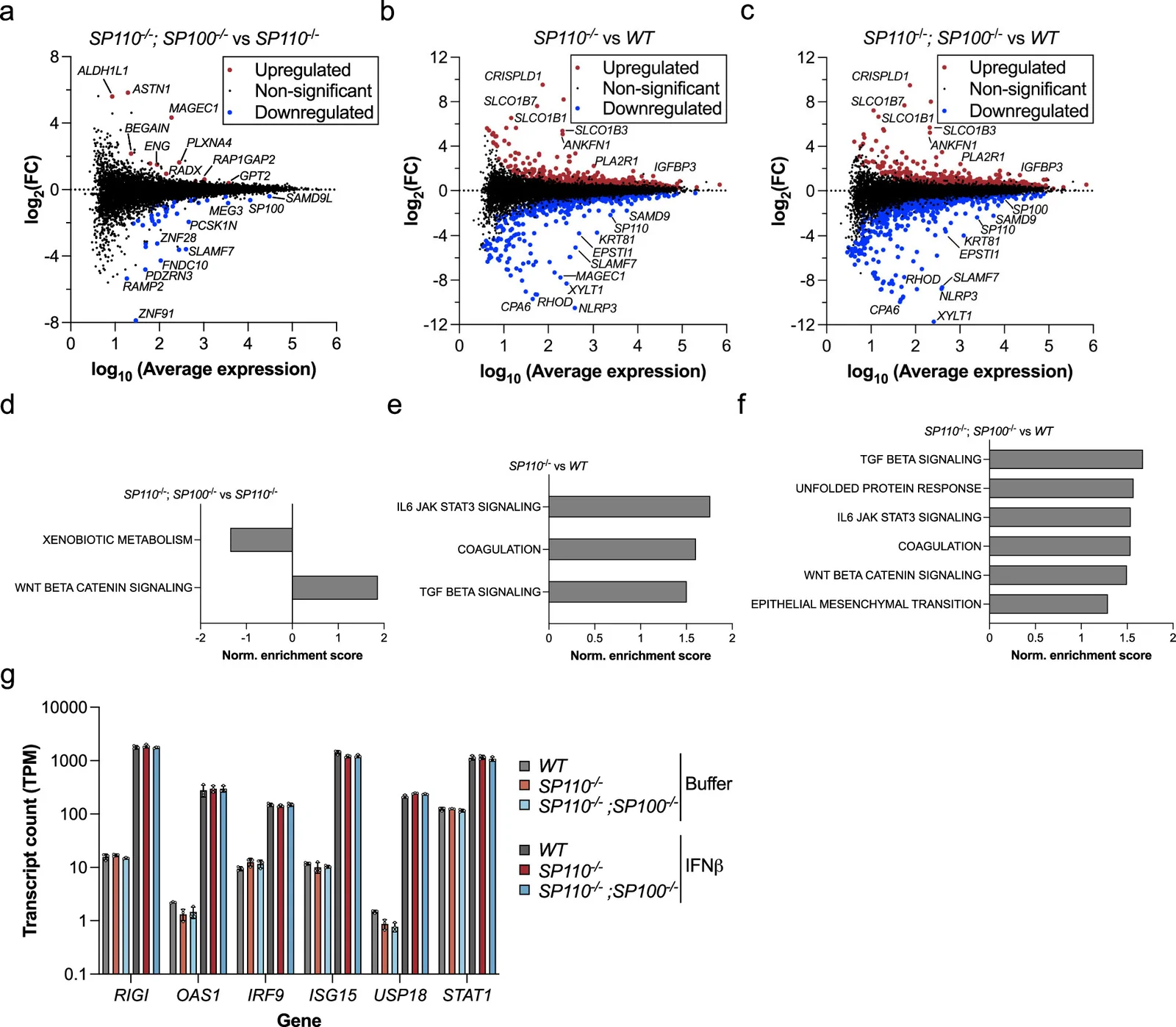
SP expression status does not broadly impact ISG expression. **a**-**c**. MA plots of log_2_ fold change (FC) in gene expression versus log_10_ average gene expression when comparing RPE1 (**a**) *SP110*;*SP100*-KO versus *SP110*-KO, (**b**) *SP110*-KO versus WT, and (**c**) *SP110*;*SP100*-KO versus WT cells as measured by RNA-seq with 6 h IFNβ stimulation. Each data point represents a gene. Red and blue data points = adjusted p-value < 0.05. Experiment was performed with biological triplicates. **d**-**f**. Gene Set Enrichment Analysis (GSEA) classified according to the Molecular Signatures Database (MSigDB) hallmark gene lists with a nominative p-value < 0.05 for the RPE1 (**d**) *SP110*;*SP100*-KO versus *SP110*-KO, (**e**) *SP110*-KO versus WT, and (**f**) *SP110*;*SP100*-KO versus WT cells comparison under IFNβ stimulated conditions. **g**. Transcript count (in transcripts per million, TPM) of various ISGs. Each data point represents an individual biological replicate (*n* = 3). Bars denote mean ± s.d. Source numerical data are available in source data. Source data

Due to its promoter localization, we initially hypothesized that SP100-induced cell death might be caused by a dysregulated interferon response. However, using RNA sequencing (RNA-seq) to assess global transcriptomic changes in stimulated WT, *SP110*-KO and *SP110;SP100* dual-KO cells, we observed few significant changes in expression (56 genes with *P*_adj_ < 0.05) between the single- and double-knockout cells (Extended Data Fig. 4a). Differentially expressed genes in WT versus *SP110*-KO and WT versus *SP110;SP100* dual-KO were frequently common between the conditions (Extended Data Fig. 4b,c). Transcriptome-wide, there was no interferon signature present among differentially expressed genes in either *SP110*-KO or *SP110;SP100* dual-KO cells when compared with WT cells (Extended Data Fig. 4d–f). All three cell types mounted an equal interferon-induced transcriptional response as measured by upregulation of common ISGs (Extended Data Fig. 4g), including *RIGI*, whose promoter is bound by SP100 in WT cells (Extended Data Fig. 3e). The interferon hypersensitive *SP110*-KO cells showed no evidence of ISG hyperactivation, and the interferon-resistant *SP110*;*SP100* dual-KO cells showed no evidence of ISG dampening. Combined with the chromatin binding data (Extended Data Fig. 3), these results suggest that the transcriptional functions of SP100 do not underlie its toxic effects in RPE1 cells.

**Extended Data Fig. 5:**
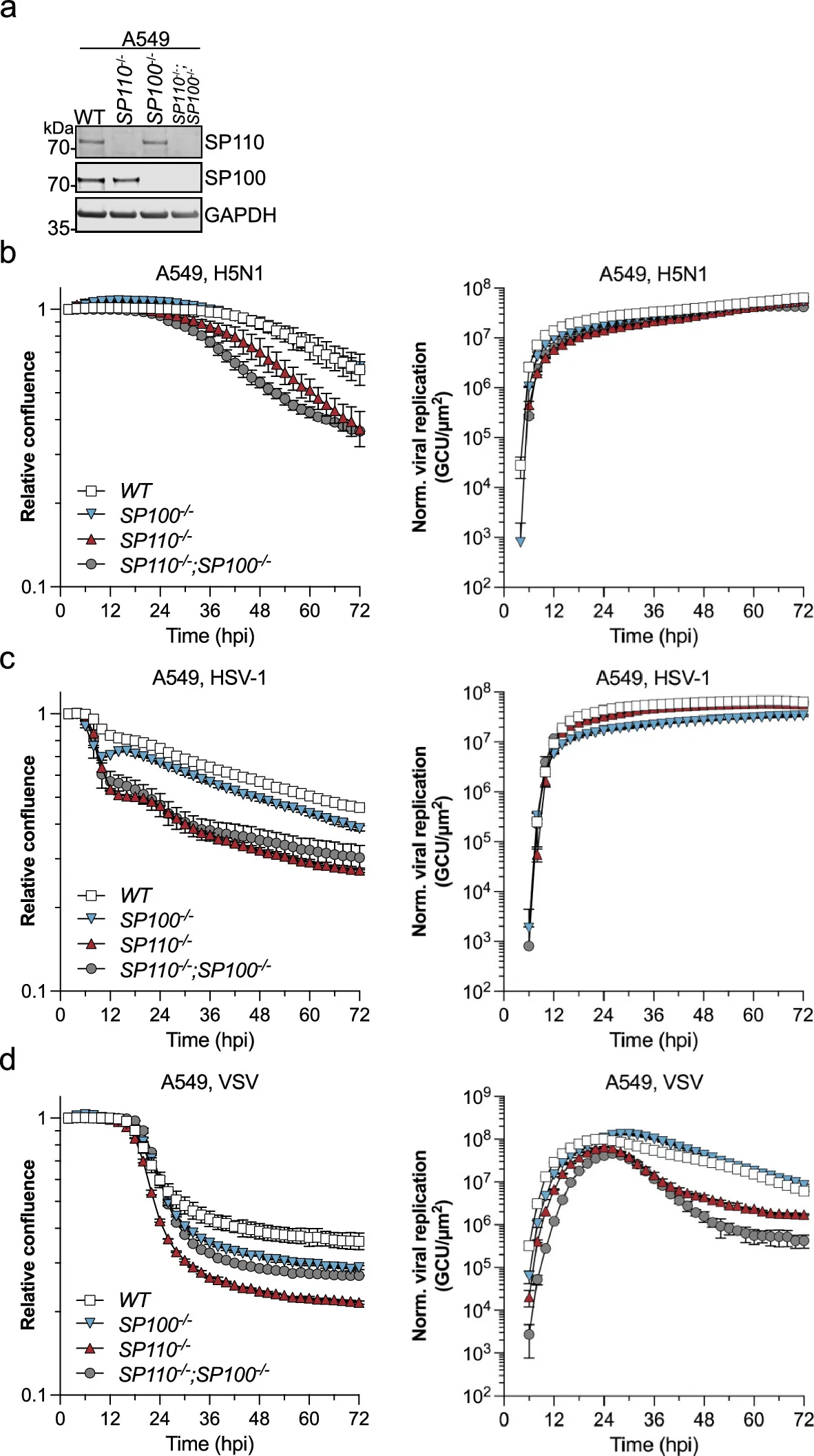
SP110 loss amplifies virus-induced cell death independent of SP100. **a**. Validation of SP100 and SP110 expression status in A549 WT pool or isogenic knockout clones. GAPDH served as the loading control. **b**-**d**. Time course of A549 cells infected with (**b**) H5N1-GFP, (**c**) HSV-1-GFP, or (**d**) VSV-GFP. Left panels: Relative cell confluence as quantified microscopically by cell area. Right panels: Normalized viral replication as quantified by GFP fluorescence measured per well area. hpi = h post-infection. Data points denote mean ± s.d. (*n* = 2 independent biological replicates). Source numerical data and unprocessed blots are available in source data. Source data

SP100 has described roles as a viral restriction factor^29,30^, and we sought to elucidate whether viral infection plays a role in the observed SP110/SP100 toxicity. We infected A549 cells (WT, *SP110*-KO, *SP100*-KO and *SP110*;*SP100* dual-KO) with three different viruses: H5N1 influenza A virus, herpes simplex virus 1 (HSV-1) and vesicular stomatitis virus (VSV) (Extended Data Fig. 5a). While we observed increased cell death in infected *SP110*-KO cells with all three viruses, this was not rescued in *SP110*;*SP100* dual-KO cells (Extended Data Fig. 5b–d). We also quantified viral replication and observed minimal differences with H5N1 or HSV-1 replication regardless of SP110 or SP100 expression status (Extended Data Fig. 5b,c). VSV replication was reduced in the absence of SP110, but this was not dependent on SP100 (Extended Data Fig. 5d). In summary, these data indicated that SP100-dependent cellular toxicity is independent of transcriptional regulation and viral replication restriction in our studied cellular contexts, suggesting unexplored cellular functions of SP100 and SP110.

### Mitotic retention of SP100 on segregating chromosomes induces genotoxicity and cell death

**Fig. 3:**
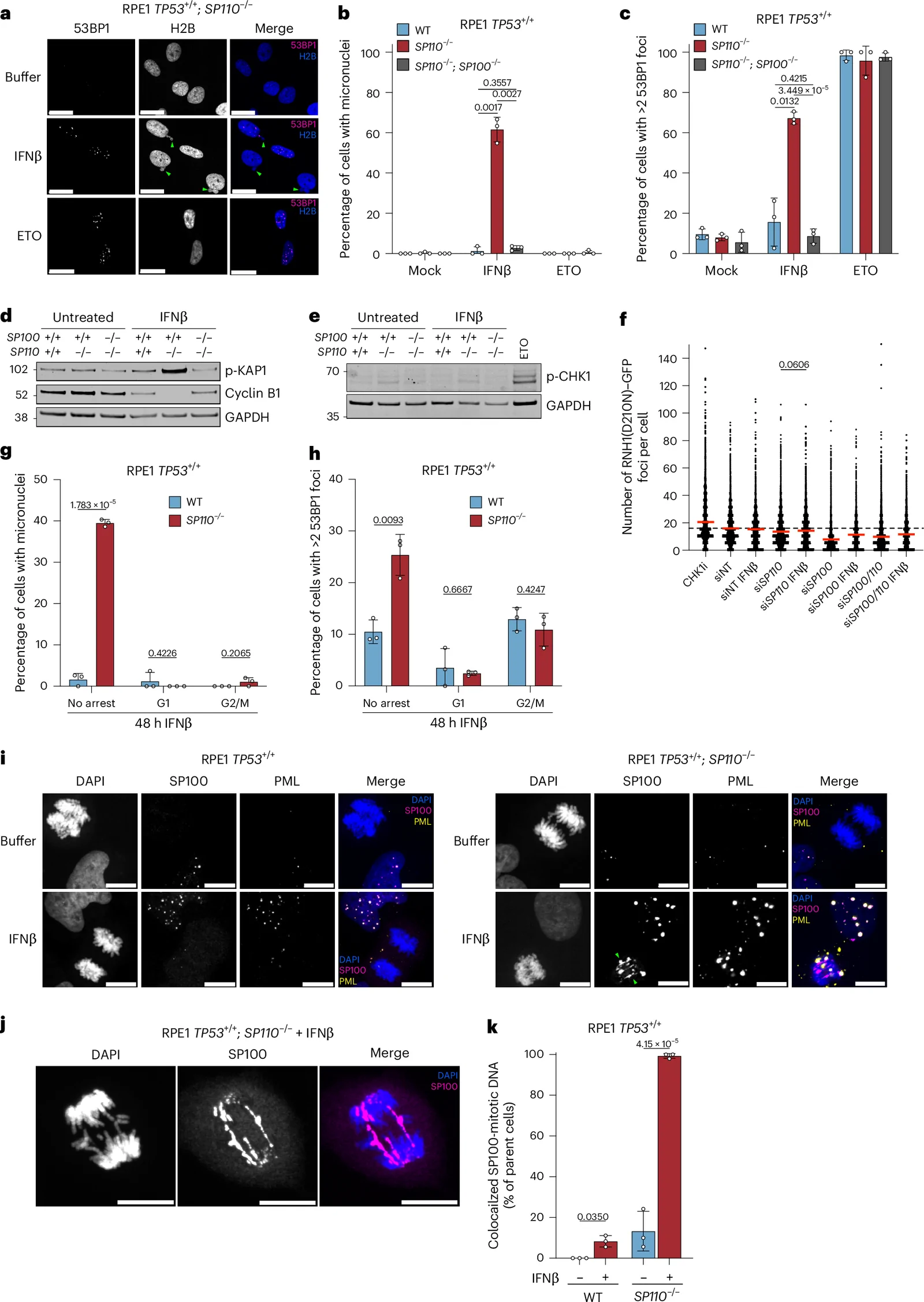
SP100 promotes genotoxicity via retention on mitotic DNA. **a**, 53BP1 immunofluorescence in RPE1 *TP53*^+/+^; *SP110*^*−/−*^ cells stably expressing H2B–GFP treated with buffer, IFNβ (10 ng ml^−1^), or etoposide (ETO; 25 µM) for 72 h. Scale bar, 20 µm. Green arrows denote micronuclei. **b**, Quantification of micronuclei from **a** and Extended Data Fig. 6a,b. Each data point corresponds to 51–100 cells (*n* = 3 biological replicates). **c**, Quantification of cells containing >2 53BP1 foci 72 h post-treatment from **a** and Extended Data Fig. 6a,b. Each data point corresponds to 51–100 cells (*n* = 3 biological replicates). **d**, Western blot of whole cell lysates from untreated and 72-h IFNβ-treated RPE1 *TP53*^+/+^ cells of the indicated genotype probing for phosphorylated KAP1 (S824) and cyclin B1 with GAPDH as the loading control. Molecular weight in kDa. **e**, Western blot of whole-cell lysates from untreated and 72 h IFNβ-treated RPE1 *TP53*^+/+^ cells of the indicated genotype probing for phosphorylated CHK1 (S824) and cyclin B1 with GAPDH as the loading control. Molecular weight in kDa. **f**, Quantification of catalytically inactive GFP–RNaseH1^D210N^ foci as a readout of R-loops in U2OS cells. The dotted line corresponds to the siNT (non-target control). Red bars correspond to the mean. Representative images are shown in Extended Data Fig. 6d. **g**, Quantification of micronuclei 48 h post-IFNβ treatment combined with continuous G1 or G2/M arrest. Each data point corresponds to 51–100 cells (*n* = 3 biological replicates). **h**, Quantification of cells containing >2 53BP1 foci 48 h post-treatment with IFNβ combined with continuous G1 or G2/M arrest. Each data point corresponds to 51–100 cells (*n* = 3 biological replicates). **i**, Representative SP100 and PML immunofluorescence in mitotic RPE1 cells in the indicated genotypes and treatment conditions. Green arrows denote SP100 retained on mitotic DNA. Scale bar, 10 µm. **j**, SP100 immunofluorescence in an IFNβ-stimulated mitotic RPE1 *TP53*^+/+^; *SP110*^−/−^ cell. Scale bar, 10 µm. **k**, Quantification of SP100 retained on mitotic DNA. Each data point corresponds to 20 mitotic cells (*n* = 3 biological replicates). Bars in **b**, **c**, **g**, **h** and **k** are displayed as mean ± s.d. Statistical significance was determined with a two-tailed Welch’s *t*-test in **b**, **c**, **g**, **h** and **k**; Kruskal–Wallis test in **f**. Source data

**Extended Data Fig. 6:**
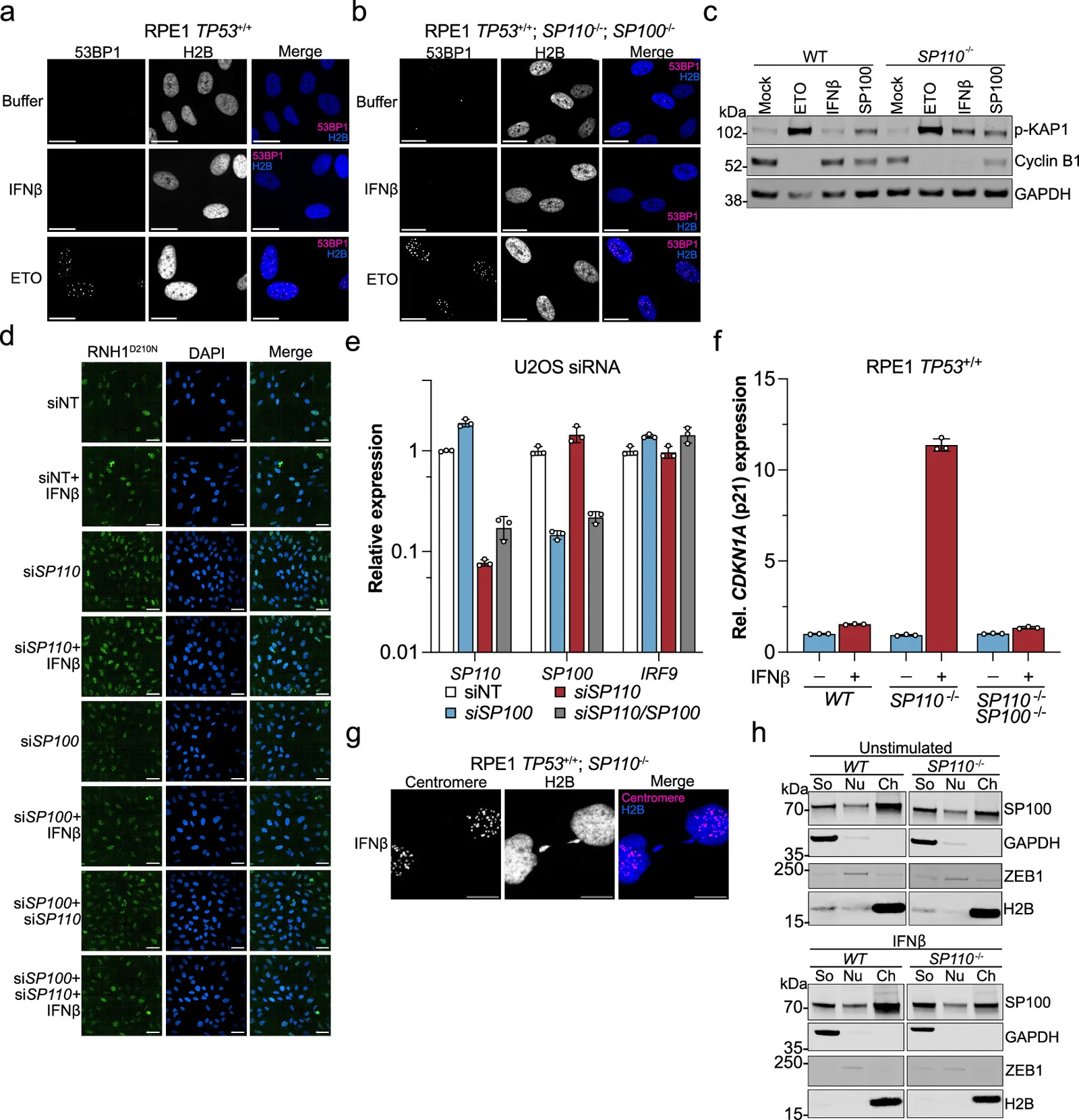
SP100 induces mitotic genotoxicity. **a**,**b**. 53BP1 immunofluorescence in RPE1 *TP53* ^+/+^ (**a**) WT and (**b**) *SP110;SP100* dual-KO cells stably expressing H2B-GFP treated with buffer, IFNβ (10 ng/mL), or etoposide (ETO; 25 µM) for 72 h. Scale bar, 20 µm. **c**. Western blot of the lysates from the indicated RPE1 *TP53*^+/+^ genotypes treated with buffer (mock), ETO, IFNβ, or transduced with SP100 cDNA. Note the transduction efficiency was ~30% and cells were not selected for positive integrations. Lysates were probed for phosphorylated KAP1 (S824) and cyclin B1 with GAPDH serving as the loading control. **d**. Representative fluorescence images of catalytically inactivate GFP-RNaseH1^D210N^ in U2OS cells treated with the indicated siRNAs (NT = non-target). Scale bar, 50 µm. Results are quantified in Fig. 3f. **e**. Quantification of knockdown efficiencies of the labelled genes with the indicated siRNAs. Each data point represents a technical replicate. Results are representative from *n* = 2 experiments. **f**. RT-qPCR of *CDKN1A* (p21) in untreated and IFNβ treated cells of the indicated genotype. Each data point corresponds to technical replicates from a representative experiment performed *n* = 2 times. **g**. Immunofluorescence of human centromere in RPE1 *TP53*^+/+^; *SP110*^*−/−*^ cells treated with IFNβ. Scale bar, 10 µm. **h**. Western blots of cellular fractions of G2/M arrested cells to identify SP100 localization. So = soluble/cytoplasmic fraction (marked by GAPDH); Nu = nuclear soluble fraction (marked by ZEB1); Ch = chromatin fraction (marked by histone H2B). Bars in **e** and **f** denote mean ± s.d. Images in **a**, **b** and **g** are representative from *n* = 3 independent biological replicates. Western blots in **c** and **h** were performed 2 independent times with similar results. Source numerical data and unprocessed blots are available in source data. Source data

To determine the mechanism of SP100-induced cellular toxicity, we used immunofluorescence to inspect *SP110*-KO cells after IFNβ stimulation. We observed marked micronucleus formation, a hallmark of genome instability, in over 60% of *SP110*-KO cells (Fig. 3a,b). This was in comparison with <3% micronuclei in stimulated WT and *SP110;SP100* dual-KO cells (Extended Data Fig. 6a,b). We stained for 53BP1, a marker of DNA double-strand breaks, and found that 67 ± 12% of *SP110*-KO cells contained more than two 53BP1 foci compared with 16 ± 12% of WT or 9 ± 4% of *SP110;SP100* dual-KO cells in the presence of IFNβ (Fig. 3c).

Ataxia-telangiectasia mutated (ATM) signalling, activated during double-strand break formation^31^, was upregulated in IFNβ-treated *SP110*-KO cells using phosphorylated KAP1 as a marker (Fig. 3d). As with cell death, SP100 overexpression was sufficient to induce ATM signalling even in the absence of IFNβ (Extended Data Fig. 6c). ATM signalling was not upregulated in the *SP110;SP100* dual-KO cells, demonstrating that DNA damage is SP100 dependent (Fig. 3d). By contrast, we observed no ATR signalling (via CHK1 phosphorylation), which is a marker of replication stress^31^ (Fig. 3e), nor did *SP110*-KO lead to an increase in R-loops formed during transcription–replication conflicts and replication stress (Fig. 3f and Extended Data Fig. 6d,e).

Loss of cyclin B1 and activation of p53 are linked with excessive DNA damage, cell cycle arrest and the induction of cell death^32,33^. We detected an absence of cyclin B1 in interferon-stimulated *SP110*-KO cells (Fig. 3d) and when overexpressing SP100 (Extended Data Fig. 6c). We measured p53 activation using *CDKN1A* (p21) expression as a marker and again found upregulation only in *SP110*-KO cells stimulated with IFNβ (Extended Data Fig. 6f). No p21 upregulation was measured in WT or *SP110;SP100* dual-KO cells, consistent with the observed DNA damage phenotype.

Micronuclei can be cause or consequence of DNA damage. We found that preventing cycling of *SP110*-KO cells rescued the formation of both micronuclei and 53BP1 foci to WT levels (Fig. 3g,h). The observed genotoxic DNA damage is therefore dependent on mitosis, suggesting a link to chromosome segregation. The SP100-induced micronuclei contained only acentric chromosome fragments, ruling out mitotic spindle failure or kinetochore damage as the cause (Extended Data Fig. 6g). We therefore examined the dynamics of SP100 and PML bodies during mitotic chromosome segregation.

**Extended Data Fig. 7:**
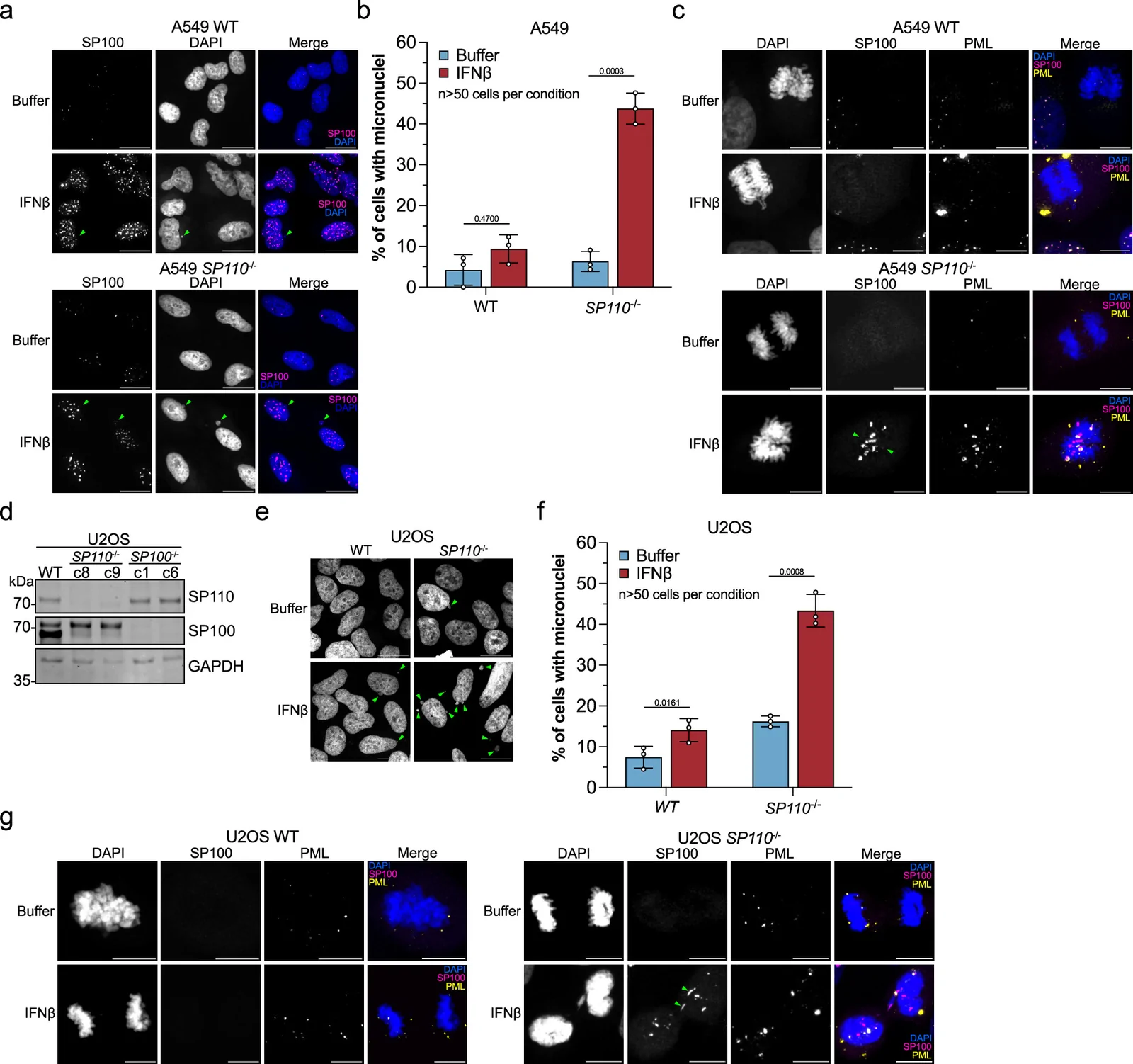
Multiple cell types exhibit mitotic SP100 retention and genotoxicity. **a**. Representative immunofluorescence images of SP100 to quantify micronuclei abundance 72 h post-treatment with buffer or IFNβ in the indicated A549 genotypes. Green arrows denote micronuclei. Scale bar, 20 µm. **b**. Quantification of micronuclei in A549 cells represented in **a**. Each data point corresponds to 51–100 cells (*n* = 3 biological replicates). **c**. Representative SP100 and PML immunofluorescence in mitotic A549 cells (WT, upper panels; *SP110* KO, lower panels). Green arrows denote SP100 retained on mitotic DNA. Scale bar, 10 µm. **d**. Western blot validation of SP100 and SP110 expression status in U2OS WT pool or isogenic knockout clones. GAPDH served as the loading control. Experiment representative of *n* = 2 independent biological replicates. **e**. Representative DAPI-stained images of U2OS cells to quantify micronuclei abundance 72 h post-treatment with buffer or IFNβ in the indicated genotypes. Green arrows denote micronuclei. Scale bar, 20 µm. **f**. Quantification of micronuclei in U2OS cells represented in **e**. Each data point corresponds to 51–100 cells (*n* = 3 biological replicates). **g**. Representative SP100 and PML immunofluorescence in mitotic U2OS cells. Green arrows denote SP100 retained on mitotic DNA. Scale bar = 10 µm. Experiment performed n = 3 independent times with similar results. Bars in **b** and **f** denote mean ± s.d. Each data point in **b** and **f** corresponds to an individual biological replicate quantifying 51–100 cells. Statistical significance in **b** and **f** was determined with a two-tailed Welch’s *t*-test. Source numerical data and unprocessed blots are available in source data. Source data

In WT and *SP110*-KO cells, SP100 and PML colocalized in interphase cells but were mostly diffuse during mitosis (Fig. 3i). This is consistent with the known mitotic disassembly of PML bodies^34^. We verified that SP100 is not relocalized or degraded during mitosis (Extended Data Fig. 6h). Remarkably, treatment of *SP110*-KO cells with IFNβ led to mitotic retention of SP100 and PML foci, although they do not exclusively colocalize (Fig. 3i). We also observed that SP100 preferentially bound to abnormally lagging chromosomes, forming prominent anaphase bridges (Fig. 3j). SP100 was retained on nearly 100% of mitotic *SP110*-KO cells stimulated with IFNβ (Fig. 3k). We also found IFNβ-induced micronuclei formation and mitotic retention of SP100 and PML in *SP110*-KO A549 lung carcinoma (Extended Data Fig. 7a–c) and U2OS osteosarcoma cells (Extended Data Fig. 7d–g), indicating that this phenotype is conserved across multiple cell types.

**Extended Data Fig. 8:**
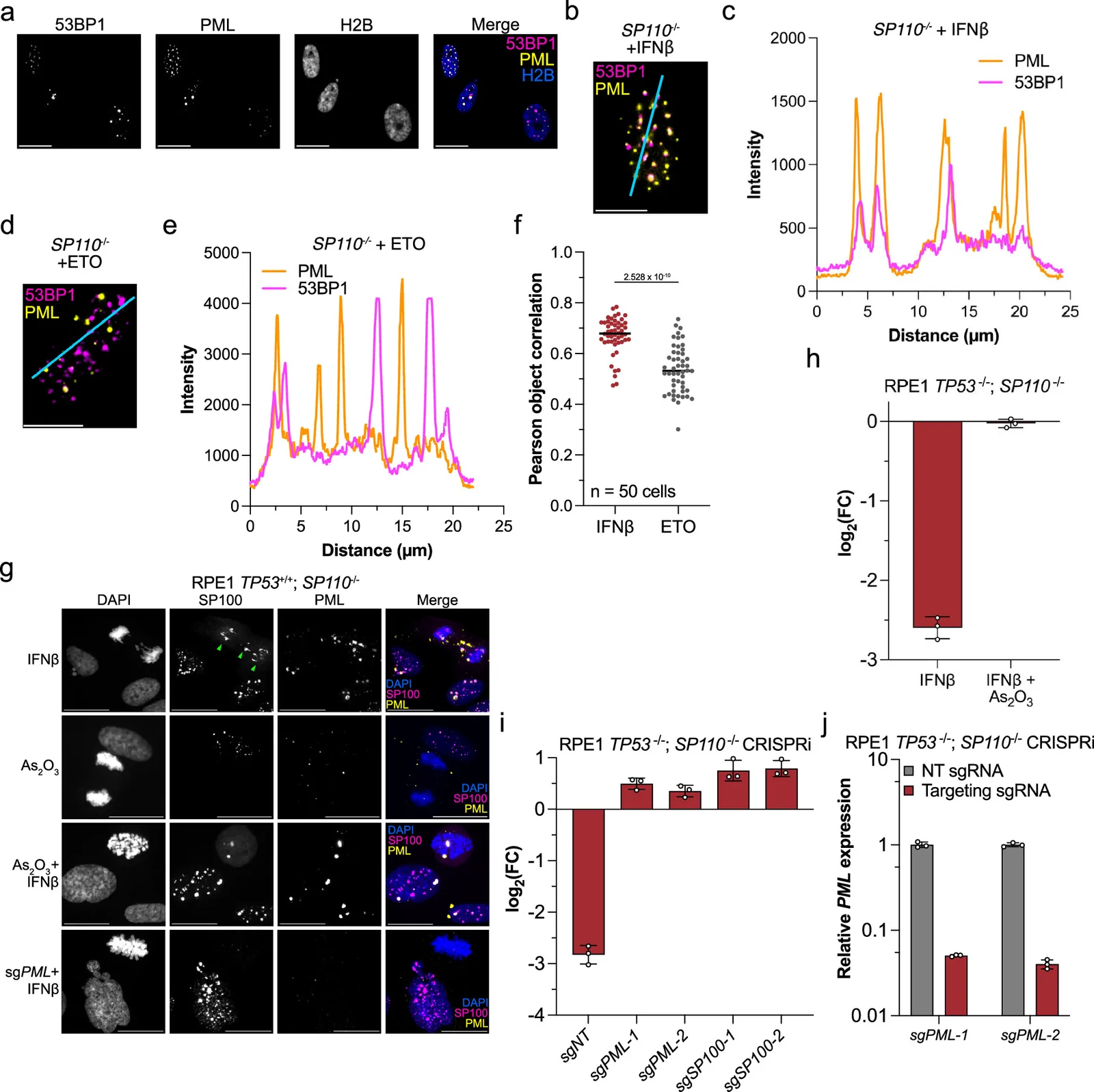
PML bodies colocalize with SP100-induced DNA damage. **a**. Immunofluorescence in RPE1 *SP110*-KO cells stably expressing H2B-GFP stimulated with IFNβ staining for 53BP1 and PML. Scale bar, 20 µm. **b**. Individual IFNβ treated *SP110*-KO cell stained with 53BP1 (magenta) and PML (yellow) with blue profile line drawn across. Scale bar, 10 µm. **c**. Quantification of intensity across the blue profile line in **b**. **d**. Individual etoposide treated *SP110*-KO cell stained with 53BP1 (magenta) and PML (yellow) with blue profile line drawn across. Scale bar, 10 µm. **e**. Quantification of intensity across the blue profile line in **d**. **f**. Pearson object correlation of colocalization between PML and 53BP1 foci. Each data point represents one cell (*n* = 50 for each condition). Bars denote the median. A Mann-Whitney U test was used to determine statistical significance. **g**. Representative SP100 and PML immunofluorescence images in RPE1 *SP110* KO cells treated with the indicated conditions. sg*PML* = CRISPRi knockdown of *PML*. Green arrows denote SP100 retained on mitotic DNA. Scale bar, 20 µm. **h**. Competition assay comparing ratio of RPE1 *SP110* KO versus WT cells on day 7 with the indicated treatments. **i**. Competition assay comparing the ratio of RPE1 *SP110*-KO CRISPRi cells expressing the indicated sgRNA versus *SP110* KO CRISPRi cells treated with IFNβ (*n* = 3 independent biological replicates). **j**. Knockdown verification of both *PML* targeting sgRNAs. Data points are technical replicates. Data is representative of *n* = 2 independent biological replicates. Bars in **h**-**j** denote mean ± s.d. Immunofluorescence experiments were performed *n* = 3 independent times with similar results. Source numerical data are available in source data. Source data

We observed strong colocalization of 53BP1 and PML foci in interphase IFNβ-treated *SP110*-KO cells (*r*_median_ = 0.68), further indicating that DNA damage following dysfunctional chromosome separation is coupled to the retention of PML bodies (Extended Data Fig. 8a–c). Etoposide-treated cells exhibited less overlap between 53BP1 and PML bodies than IFNβ-treated *SP110*-KO cells (*r*_median_ = 0.53; Extended Data Fig. 8d–f), showing that PML colocalization is not a general property of DNA damage. This suggested to us that retention of SP100-containing PML bodies was causative of chromosome lag, followed by micronucleus formation and chromosome shattering. Genetic or chemical perturbation with As_2_O_3_ of PML in *SP110*-KO cells abrogated IFNβ-induced SP100 retention (Extended Data Fig. 8g). Moreover, KD of *PML* or As_2_O_3_ treatment was sufficient to rescue IFNβ-induced cell death in *SP110*-KO cells (Extended Data Fig. 8h–j). Hence, the opposing activities of SP110 and SP100 are upstream and causative of dysregulated PML body dynamics, which is the eventual cause of cell toxicity. In summary, we found that loss of SP110 led to the inappropriate mitotic retention of SP100-localized PML bodies on segregating DNA, which caused chromosome mis-segregation, genotoxicity and cell death.

### PML body homeostasis is regulated by SP100 and SP110

**Fig. 4:**
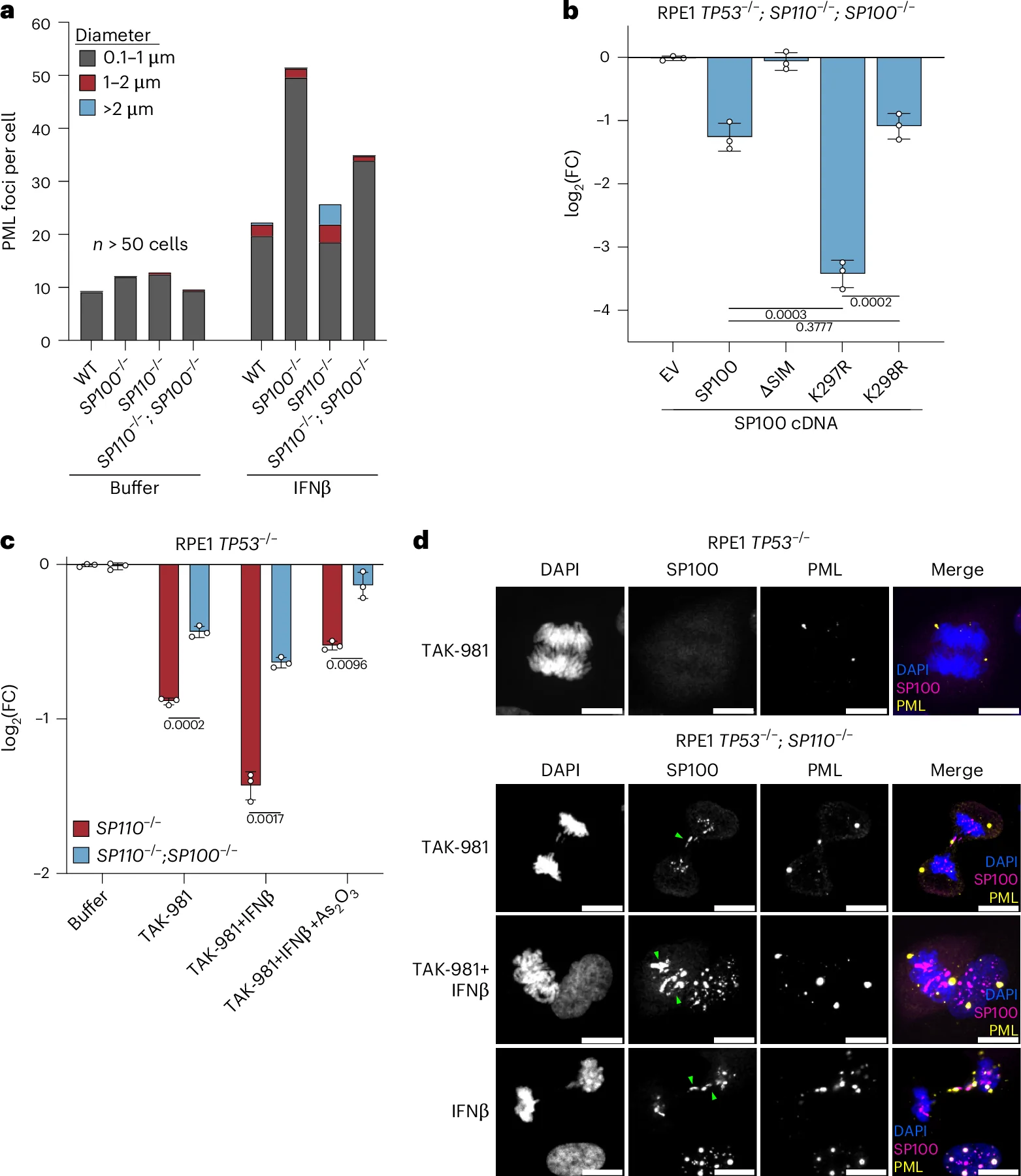
SUMO-deficient SP100 drives genotoxicity. **a**, Quantification of the mean PML body number and size in RPE1 *TP53*^−/−^ cells of the indicated genotypes and treatment conditions. PML body size is binned into three colour-coded classifications. Each bar graph corresponds to 51–100 cells from *n* = 3 independent experiments. Representative images are shown in Extended Data Fig. 9a. **b**, Competition assay in RPE1 *SP110;SP100* dual-KO cells stably expressing the indicated SP100 cDNA construct versus non-expressing cells with no immune stimulation. EV, empty vector. **c**, Competition assay comparing the indicated genotypes versus WT cells treated with pan-SUMOylation inhibitor (TAK-981), IFNβ and/or PML-body disruptor As_2_O_3_. **d**, Representative immunofluorescence of SP100 and PML in mitotic RPE1 cells with the indicated genotypes and treatment conditions. Green arrows denote SP100 retained on mitotic DNA. Scale bar, 10 µm. Each data point in **b** and **c** represents an independent biological replicate (*n* = 3). Bar graphs in **b** and **c** are displayed as mean ± s.d. Statistical significance was determined with a two-tailed Welch’s *t*-test in **b** and **c**. Source data

**Extended Data Fig. 9:**
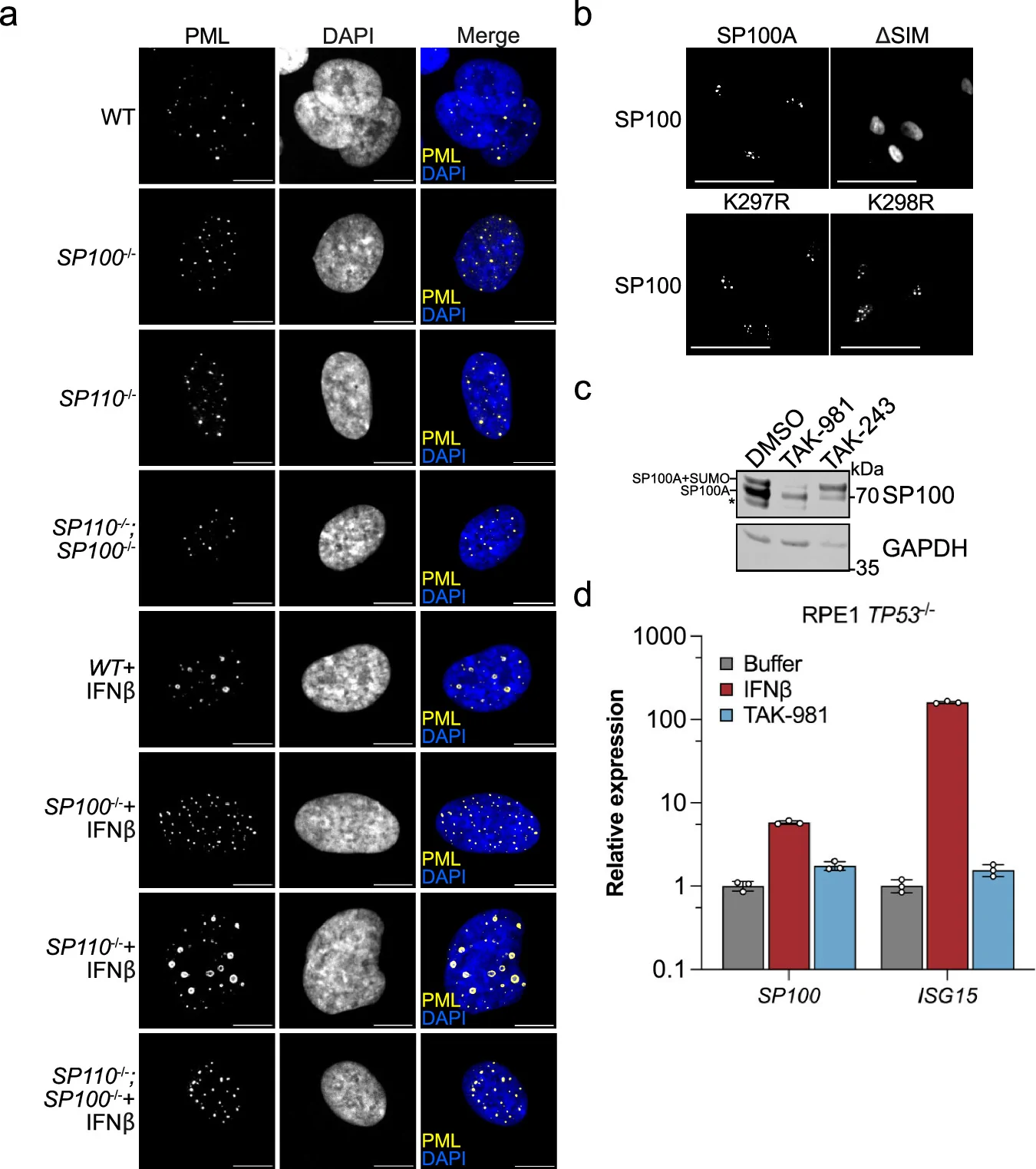
PML body and SUMOylation are important in SP100-induced toxicity. **a**. Representative PML immunofluorescence images used to quantify PML body size and number in Fig. 4a in RPE1 *TP53*^*−/−*^ cells. Scale bar, 10 µm. **b**. Fluorescence images of stably expressed mCherry-SP100 variants. SIM = SUMO interacting motif. K297R = SUMO-deficient SP100. Scale bar, 100 µm. **c**. Western blot assessing SUMOylation status of SP100 when subjected to pan-SUMOylation inhibitor TAK-981 or ubiquitin inhibitor TAK-243 in RPE1 *TP53*^*−/−*^ cells. **d**. Quantification of *SP100* or *ISG15* upregulation upon 24 h treatment with IFNβ or TAK-981 in RPE1 *TP53*^*−/−*^ cells. Each data point represents a technical replicate. Data is representative of *n* = 2 independent biological replicates. Bars denote mean ± s.d. Source numerical data and unprocessed blots are available in source data. Source data

We hypothesized that SP110/SP100 expression status could alter the properties of PML bodies, resulting in their retention on mitotic DNA. We quantified both the number and size of PML bodies with and without interferon stimulation in interphase cells. While PML body number and size were relatively consistent in unstimulated cells, we observed striking differences in IFNβ-treated cells (Fig. 4a and Extended Data Fig. 9a). In *SP110*-KO cells, PML bodies were larger relative to WT cells, 15% of which were >2 µm in diameter. Conversely, in *SP100*-KO cells, the number of PML bodies increased but were proportionally smaller than WT (96% were <1 µm).

PML body dynamics are regulated by SUMOylation^35,36^, and SP100 possesses both a SUMO-interacting motif (SIM) and a SUMO acceptor residue (K297)^37^. We asked how these molecular features regulate SP100-induced genotoxicity. In complementation experiments in *SP110*;*SP100* dual-KO cells, overexpression of SP100 ΔSIM failed to induce lethality and displayed diffuse nuclear localization (Fig. 4b and Extended Data Fig. 9b). By contrast, SUMO-deficient SP100-K297R elicited increased toxicity relative to WT or mutation of the neighbouring SP100-K298R (Fig. 4b). We also noted that *SP110* appears as a top hit in a genome-wide CRISPR screen for increased sensitivity to a pan-SUMO inhibitor (TAK-981), although this was not mechanistically characterized^38^. These results suggested that un-SUMOylated SP100 drives toxicity in *SP110*-KO cells.

We validated that broadly inhibiting SUMOylation with TAK-981 leads to cell death specifically in *SP110*-KO cells relative to WT cells, even in the absence of interferon stimulation (Fig. 4c and Extended Data Fig. 9c). TAK-981 did not induce a robust type 1 interferon response or *SP100* upregulation in RPE1 cells (Extended Data Fig. 9d). Notably, TAK-981 toxicity was significantly abrogated in *SP110*;*SP100* dual-KO cells (Fig. 4c). Moreover, perturbation of PML bodies with As_2_O_3_ in *SP110*-KO cells even mitigated the increased toxicity caused by combined treatment with IFNβ and TAK-981. TAK-981 treatment was also sufficient to induce mitotic retention of SP100 on lagging chromosomes in *SP110*-KO cells but not WT cells, and this was further exacerbated by IFNβ treatment (Fig. 4d). Taken together, these data demonstrated that SP100 SUMOylation is required for dissolution of SP100-containing PML bodies during mitosis, and that SP110 is critical to prevent the accumulation of enlarged PML bodies and toxic, un-SUMOylated SP100 that results in chromosome segregation errors.

### The SP110 CARD prevents SP100 CARD-induced lethality

**Fig. 5:**
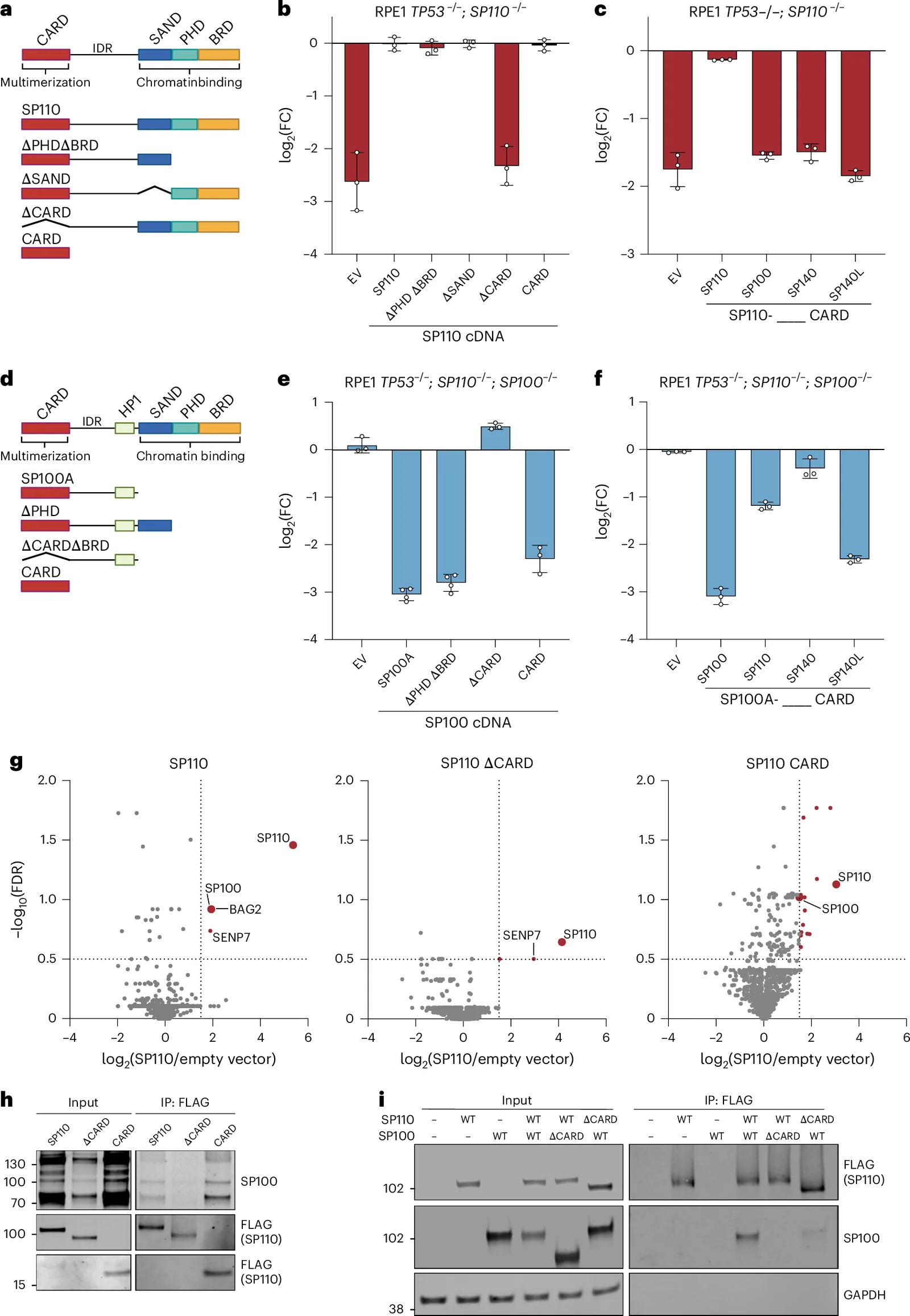
Physical SP110–SP100 CARD–CARD interaction prevents lethality. **a**, Schematic of SP110’s annotated domains. IDR, intrinsically disordered region. **b**, Competition assay comparing *SP110*-KO cells complemented with the indicated stably transduced SP110 domain mutants versus WT cells treated with IFNβ. **c**, Competition assay comparing *SP110*-KO cells complemented with the indicated stably transduced construct versus WT cells treated with IFNβ. The SP110 CARD was replaced with the CARD from the indicated speckled protein. **d**, Schematic of SP100’s annotated domains. HP1, heterochromatin protein 1. **e**,**f**, Rescue experiments of stably transduced mCherry-SP100 domain mutants in RPE1 *SP110*;*SP100* dual-KO cells (**e**), and with the SP100 CARD replaced with the CARD from the indicated speckled protein (**f**). **g**, Limma output of IP–MS spectral counts with the listed stably overexpressed FLAG–SP110 bait protein in *SP110*-KO cells. Dotted lines indicate thresholds at *x* = 1.5 and *y* = 0.5. Proteins lying above both thresholds are labelled in red. **h**, FLAG co-IP in RPE1 cells of stably expressed FLAG–SP110 variants. Molecular weight in kDa. Experiment was performed *n* = 3 independent times with similar results. **i**, FLAG co-IP in HEK-293T cells transiently overexpressing full-length (WT) or CARD deletion (ΔCARD) SP110 and SP100. Molecular weight in kDa. Experiment was performed *n* = 4 independent times with similar results. Each data point in **b**, **c**, **e** and **f** represents an independent biological replicate (*n* = 3–4). Bar graphs are displayed as mean ± s.d. Schematics in **a** and **d** created in BioRender: Corn, J. https://biorender.com/hcz7tbl (2026). Source data

**Extended Data Fig. 10:**
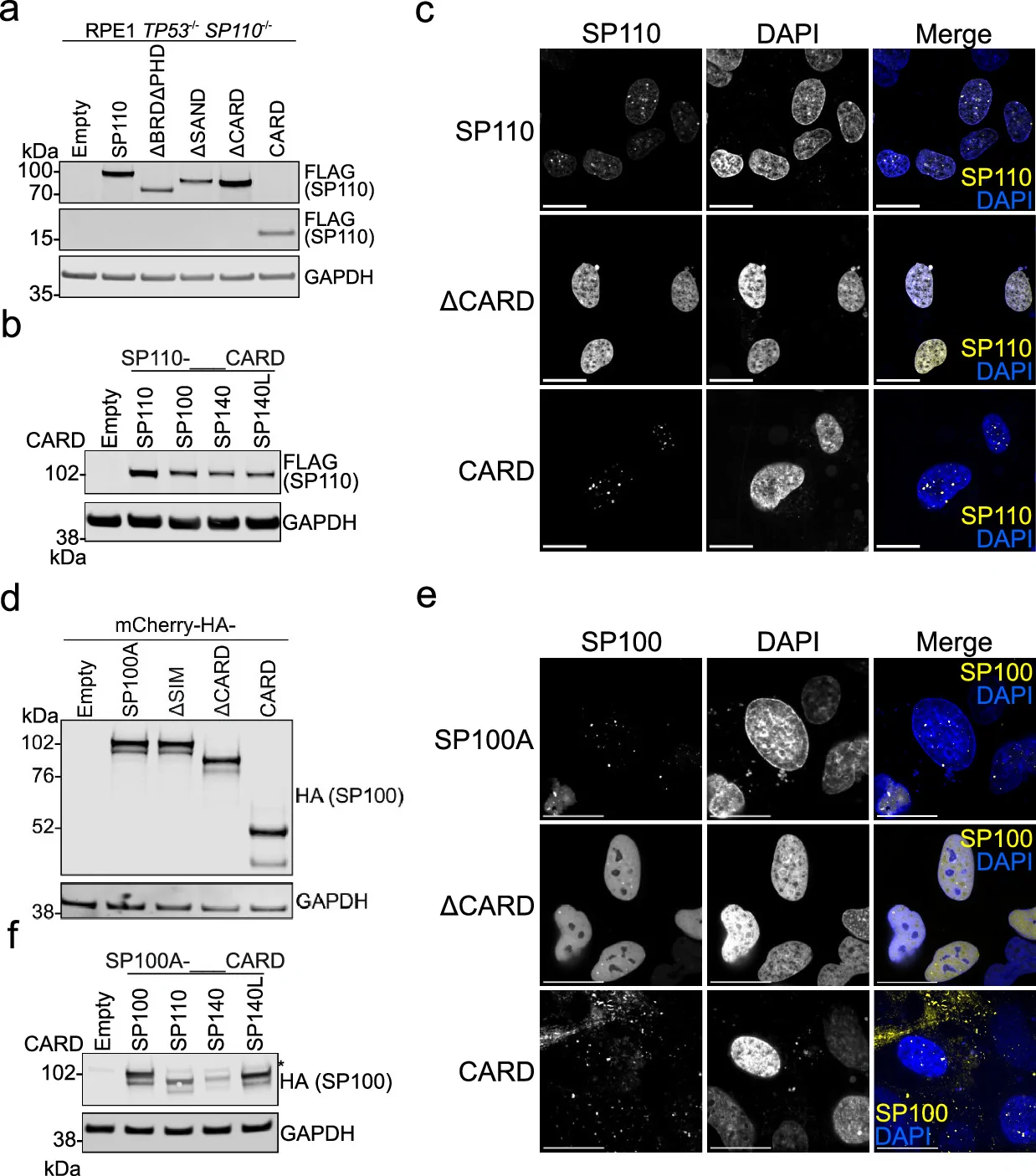
SP110 and SP100 localization and complementation expression. **a**. Western blot of stably expressed FLAG-SP110 variants in RPE1 *SP110*-KO background. **b**. Western blot of stably expressed FLAG-SP110 CARD variants RPE1 *SP110*-KO background. The indicated CARD replaces the SP110 CARD in the full-length SP110. **c**. Fluorescence microscopy imaging of stably expressed SP110-GFP (yellow) constructs in RPE1 WT cells treated with IFNβ. Scale bar, 20 µm. **d**. Western blot of stably expressed mCherry-HA-SP100 variants in RPE1 *SP100*-KO background. **e**. Fluorescence microscopy imaging of stably expressed mCherry-SP100 (yellow) constructs in RPE1 WT cells treated with IFNβ. Scale bar, 20 µm. **f**. Western blot of stably expressed mCherry-HA-SP100 CARD variants RPE1 *SP100*-KO background. The indicated CARD replaces the SP100 CARD in SP100A. All experiments were performed 2–3 independent times with similar results. Unprocessed blots are available in source data. Source data

All four speckled proteins contain C-terminal chromatin reader domains—Sp100, AIRE-1, NucP41/75, DEAF-1 (SAND), plant homeobox domain (PHD) and bromodomain (BRD)—that enhance their transcriptional regulatory potential, as well as an N-terminal CARD multimerization domain, for which no specific function has been attributed^23^. We generated domain deletion SP110 cDNAs for stable lentiviral re-expression in *SP110*-KO cells and tested their ability to rescue IFNβ-induced cell death (Fig. 5a and Extended Data Fig. 10a). In line with the lack of a transcriptional phenotype (Extended Data Fig. 4), the PHD, BRD and SAND domains were dispensable. Instead, SP110’s CARD was necessary to rescue cell toxicity (Fig. 5b). Moreover, expression of the SP110 CARD domain alone was sufficient to rescue cell death to a level comparable with full-length SP110. The SP CARDs have high sequence homology^23^, but we found that only the SP110 CARD could prevent interferon-induced toxicity (Fig. 5c and Extended Data Fig. 10b). Using confocal imaging, we found that the SP110 CARD is also required for nuclear focus formation (Extended Data Fig. 10c). These data reveal a distinct role for the SP110 CARD in protecting from cell death.

We applied the same structure–function analysis to SP100 in *SP110*;*SP100* dual-KO cells, testing the ability of SP100 constructs to induce cell death (Fig. 5d and Extended Data Fig. 10d). Like SP110, we found that PHD, BRD and SAND domains of SP100 were dispensable for cell death, but the CARD was necessary (Fig. 5e). Strikingly, the SP100 CARD alone was sufficient to induce cell death, an inverse of protection with the SP110 CARD alone. Like the SP110 CARD, SP100 localization to nuclear foci required its CARD (Extended Data Fig. 10e). We substituted other SP CARDs into SP100 and found they did not induce the same degree of toxicity as SP100’s CARD, except for SP140L, which has an 85% sequence homology due to a partial gene duplication of the SP100 CARD in primates^39^ (Fig. 5f and Extended Data Fig. 10f). In summary, SP100 toxicity relies solely on its CARD domain and is independent of its chromatin-binding domains.

CARDs are often involved in protein–protein interactions to form homo- and hetero-CARD–CARD oligomers^40^. We screened for interactors of SP110’s CARD using immunoprecipitation followed by mass spectrometry (IP–MS) with stably expressed full-length SP110, SP110 ΔCARD and SP110 CARD alone in *SP110*-KO cells. Only three proteins were enriched with full-length SP110: BAG2, the SENP7 deSUMOylase and SP100 (Fig. 5g). Strikingly, SP100 was enriched with full-length SP110 and CARD only, but not ΔCARD, suggesting a heteromeric CARD–CARD interaction. No other CARD-containing proteins, including other SPs or caspases, were enriched in any condition. We used co-immunoprecipitation (co-IP) and western blotting to confirm that SP110 and SP100 interact with each other, and that this interaction strongly depends on the CARD domain of each protein (Fig. 5h,i). Overall, our data reveal that the respective protective and cell killing activities of SP110 and SP100 are mediated by their CARD domains, that these proteins bind one another through a CARD–CARD interaction and that loss of this interaction renders cells hypersensitive to interferon signalling.

### SP110 modulates oligomerization of SP100 CARD

**Fig. 6:**
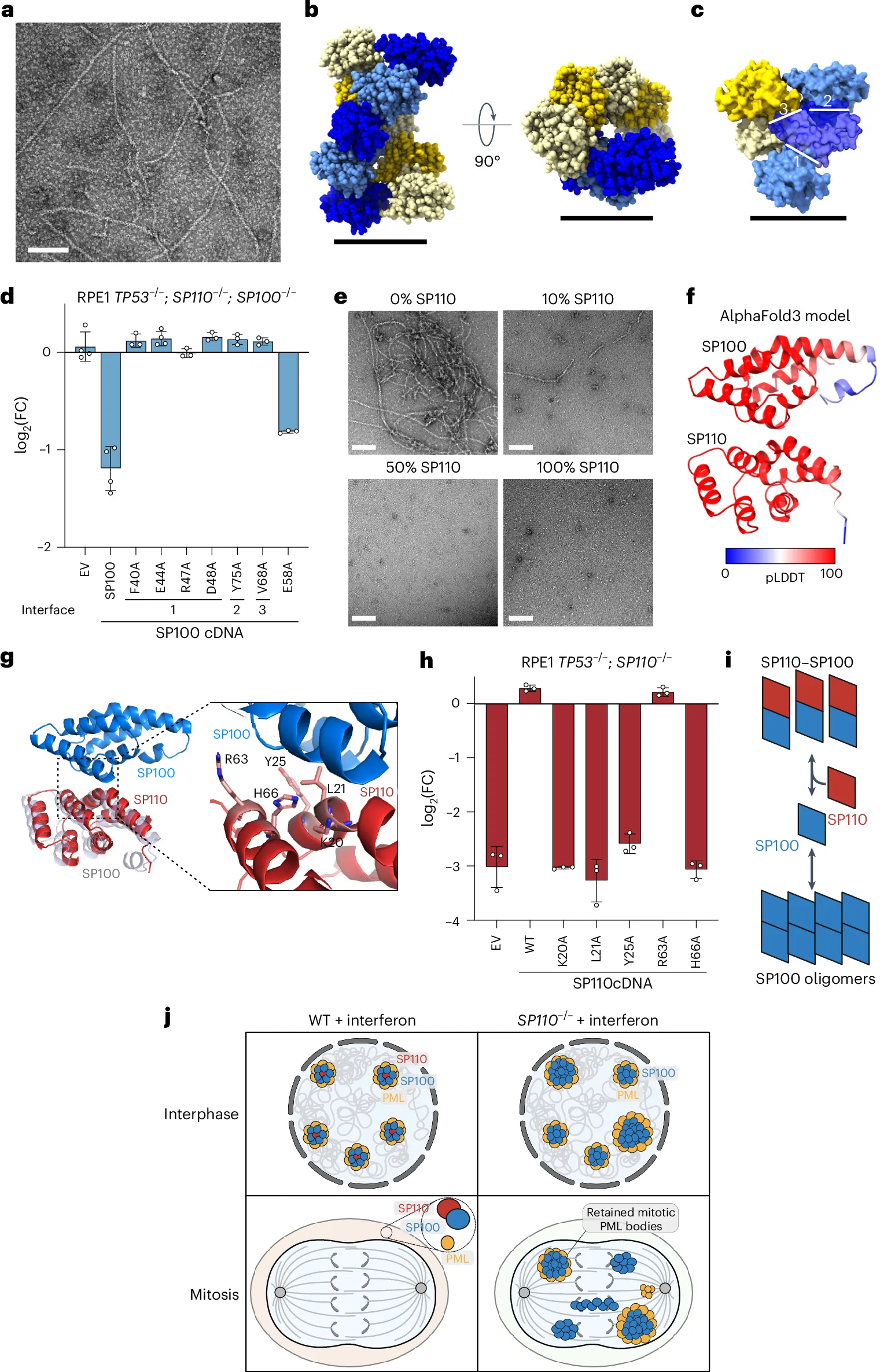
SP110 blocks toxic SP100 CARD oligomerization. **a**, TEM image of negatively stained recombinantly expressed and purified SP100 CARD. Scale bar, 100 nm. The experiment was performed *n* = 5 times with similar results. **b**, Cryo-EM structure of the SP100 oligomer. Scale bar, 50 Å. **c**, Representation of CARD–CARD binding interfaces in the SP100 filament. The three major interfaces are indicated. Scale bar, 50 Å. **d**, Rescue experiments of stably transduced SP100-T2A-mCherry point mutants in *SP110*;*SP100* dual-KO cells. EV, empty vector. **e**, TEM images of mixed SP100:SP110 with the indicated amount of SP100. Scale bar, 100 nm. Experiment was performed *n* = 2 times with similar results. **f**, AlphaFold3 model of an SP100-SP110 CARD heterodimer (ipTM 0.85). Colour coding is by predicted local distance difference test (pLDDT). **g**, AlphaFold3 model of a SP100 (blue)–SP110 (red) CARD heterodimer. The AlphaFold3 dimer is superimposed on an SP100–SP100 CARD dimer obtained from the cryo-EM structure using the blue SP100 monomer as the anchor. The inset highlights the SP110–SP100 CARD–CARD binding interface, and the predicted important residues on SP110 are labelled. **h**, Competition assay comparing *SP110*-KO cells complemented with stably transduced SP110 point mutants versus WT cells treated with IFNβ. **i**, Simplified model for SP110 sequestration of SP100 to prevent SP100 oligomerization. **j**, Model of the relationship between SP110, SP100 and PML in interferon stimulated WT and *SP110*-KO cells. Each data point in **d** and **h** represents an independent biological replicate (*n* = 3–4). Bar graphs are displayed as mean ± s.d. Schematic in **j** created in BioRender; Corn, J. https://biorender.com/hcz7tbl (2026). Source data

CARD domains can form heteromeric interactions with other CARDs and homo-oligomeric assemblies with themselves^41–43^. To explore the oligomeric state of the SP100 and SP110 CARDs, we recombinantly expressed and purified each CARD. Using negative staining and imaging by transmission electron microscopy (TEM), we observed that the SP100 CARD forms multimicrometre-long homo-oligomers in vitro (Fig. 6a). The SP100 CARD homo-oligomers potentially represented the toxic species that led to PML body enlargement and mitotic retention. Therefore, we used cryo-EM to solve a 3.5-Å structure and obtain atomic insights into its mechanism (Supplementary Fig. 2 and Supplementary Table 1). The SP100 CARD oligomer is composed of two antiparallel strands that form a double helix (Fig. 6b). The oligomer is 65 Å wide with a 10 Å central cavity and a twist length of 120 Å. Despite similar tertiary structures^44^, this quaternary architecture stands in contrast to CARD oligomers of MAVS^42^ and NLRC4^43^, alluding to different functional niches (Supplementary Fig. 3a–g).

Each SP100 CARD, consisting of six alpha helices, has two interaction interfaces in *cis* on the same strand and one interaction interface in *trans* to the opposite strand (Fig. 5c). We stably expressed SP100 with alanine point mutations at each binding interface in *SP110*;*SP100* dual-KO cells. Each single interface mutation was sufficient to completely prevent lethality to a similar extent as complete *SP100* KO, whereas a mutation outside of the interface still induced cell death (Fig. 6d and Supplementary Fig. 3h,i). Therefore, the SP100 homo-oligomeric CARD interactions contribute to cellular toxicity and may constitute the molecular basis of the SP100- and PML-containing structures retained on lagging anaphase chromosomes.

We observed no oligomer formation with recombinantly expressed SP110 CARD (Supplementary Fig. 3j). However, when testing the in vitro interaction between SP100 and SP110 CARDs, we found that SP110 had a striking ability to disassemble SP100 filaments. We mixed SP100 and SP110 at various ratios and imaged with TEM. As little as 1:10 SP110 CARD was sufficient to break SP100 oligomers, and a 1:1 ratio led to a complete loss of visible assemblies (Fig. 6e). We used Alphafold3^45^ to model an SP110–SP100 CARD heterodimer, obtaining a strong interface predicted template modelling (ipTM) score of 0.85 (Fig. 6f). This modelled heterodimer is predicted to block SP100 homo-oligomer formation, which would be consistent with SP110’s ability to support mitotic dissolution of the SP100-containing PML bodies (Fig. 6g). We tested this hypothesis by creating point mutations in full-length SP110. We found that four of five SP110 point mutations at the putative interface with SP100 (interface #1) were sufficient to cause IFNβ-induced cell death to a similar extent as complete *SP110* KO (Fig. 6h and Supplementary Fig. 3k). Taken together, these data reveal the molecular details of an SP100/SP110 CARD–CARD switch that regulates the dissolution of PML bodies during mitosis to support normal chromosome segregation (Fig. 6i).

## Discussion

In this study, we discovered that SP110 controls SP100 oligomerization, which in turn regulates the properties and mitotic dissolution of nuclear PML bodies. When SP110 is deficient, any condition that results in excess un-SUMOylated SP100 leads to inappropriate focal accumulation of PML bodies on mitotic DNA that restricts chromosome segregation, causing genotoxicity and cell death (Fig. 6j). The SP100–SP110 axis is molecularly achieved by newly described functions for the SP100 and SP110 CARD domains that mediate the assembly and disassembly of SP100 oligomers.

Human SPs share similar domain architecture yet perform distinct cell-type-specific functions^23^. Here, we establish pleiotropic functions of SP110 and SP100 that are independent of their reported roles in transcription and viral defence^23,24,30^. Although our study was initiated in the context of an interferon response, the roles of SP100 and SP110 are not confined to innate immunity. Both proteins are widely expressed across human cell types^46^, suggesting that their regulation of PML body homeostasis is not a specialized immune function but rather a fundamental process of cellular maintenance. Moreover, control of the SP110–SP100 axis is not restricted to transcriptional upregulation as SUMOylation inhibition induced mitotic SP100 retention (Fig. 4d). Differential SP110 phosphorylation was also observed in response to interferon signalling^47^, implying that other post-translational modifications may impact the SP110–SP100 axis. By contrast, human SP140 expression is largely restricted to immune cells, where it maintains heterochromatin status by repressing topoisomerases and governing macrophage cell fate^48,49^. This functional divergence positions the SP110/SP100 partnership as distinct from the more specialized, lineage-specific roles of SP140.

SP proteins, including SP100 and SP110, are best known for their roles in chromatin transactions^23^. However, we find that the activity of SP110 and SP100 in cell death strictly involves the CARDs of each protein to regulate PML body homeostasis. SP100’s cell killing capacity is achieved through the formation of un-SUMOylated CARD-mediated oligomers that prevent mitotic dissolution. Other proteins form CARD-dependent oligomers, but these are distinct in both structure and function^16,42,43^ (Supplementary Fig. 3). AIRE, a close relative of SPs, forms chromatinized homomultimers to facilitate transcriptional activity in T cells via its PHD^50,51^. This contrasts with SP100’s CARD homo-oligomerization that functions independent of its chromatin reading domains. MAVS forms CARD oligomers that amplify the RIG-I signalling cascade within ZCCHC3 phase-separated bodies^7,42^. Substituting the MAVS CARD with the CARD from ASC prevents recruitment into ZCCHC3 condensates^7^, which parallels our observation that SP CARDs are not interchangeable in regulating SP100-induced toxicity. Biologically, SP110’s CARD prevents SP100 over-oligomerization in *trans*, thereby enabling mitotic PML body dissolution and preventing chromosome mis-segregation. Purposefully perturbing SP100 CARD oligomerization could therefore present intriguing therapeutic options for patients with *SP110*-deficient VODI.

Nuclear PML bodies are dramatically disassembled during mitosis and coalesce into distinct structures (mitotic accumulations of PML protein (MAPPs)) that are kept separate from condensed chromosomes^34,35,52,53^. However, the molecular basis for PML body disassembly and the potential consequences of inappropriate PML retention were previously unknown. We find that SP110 is required for dissolution of PML bodies during mitosis by regulating SP100 oligomerization. Failure to disassemble SP100 oligomers leads to chromosomal PML body retention and a pathological gain of function that compromises genome stability. Biomolecular phase-separated bodies perform myriad critical cellular functions, but emerging data indicate that they present distinct challenges to the cell and must be actively surveilled to ensure cell health and prevent disease^54–58^. For example, TDP-43 is solubilized by PML-mediated SUMOylation to protect against aggregation that manifests as amyotrophic lateral sclerosis^56^. G3BP1 controls the assembly of phase-separated cytosolic stress granules, whose misregulation has been linked to neurodegenerative disorders^13,57,58^. Our work reveals the SP100–SP110 axis as a critical rheostat for phase-separated PML body homeostasis, demonstrating that disruption of its mitotic disassembly manifests as a potent source of genomic instability. Future research will be essential to establish whether the regulated disassembly of biomolecular phase-separated bodies is a general requirement to maintain cell health, the failure of which could underpin a wide variety of human pathologies.

## Methods

### Cloning, nucleotide and protein sequences

All nucleic acid sequences can be found in Supplementary Table 2. DNA was purchased from Integrated DNA Technologies and Microsynth AG. *SP110* and *SP100* full-length and domain deletion constructs were amplified from cDNA generated from RNA isolated from RPE1 cells. Domain deletion constructs were assembled using site-directed mutagenesis. Other cloning procedures were performed using either Gibson assembly or Golden Gate assembly.

### Cell culture

hTERT-RPE1 (ATCC), HEK-293T (ATCC), U2OS (ATCC), A549 (generously provided by Dr Tim Clausen), MDCK (ATCC) and Vero-CCL81 (ATCC) cells were cultured in Dulbecco’s modified Eagle medium (DMEM, Gibco), supplemented with 10% foetal bovine serum (FBS) and 100 µg ml^−1^ penicillin–streptomycin. Jurkat (ATCC) cells were cultured in RPMI medium (Gibco), 10% FBS and 100 µg ml^−1^ penicillin–streptomycin. Phytohemagglutinin (PHA)-stimulated T lymphoblasts were cultured in RPMI medium (Gibco), 0.1% IL-2, 10% FBS and 100 µg ml^−1^ penicillin–streptomycin. All cells were incubated at 37 °C and 5% CO_2_. Cell lines were routinely tested for mycoplasma contamination and tested negative (MycoAlert; Lonza). Cell lines were short tandem repeat profiled (Microsynth AG). Cell lines used in this study are listed in Supplementary Table 3. All clonal cell lines were genotyped by Illumina sequencing and phenotyped for absence of protein production by western blot.

### Chemical treatment conditions

Recombinant human IFNβ (Abcam ab71475) was added at 10 ng ml^−1^ and incubated for 1–3 days. Poly(I:C) (HMV; Invivogen) was added at 500 ng ml^−1^ and incubated 72 h. Poly(dA:dT) (Invivogen) was added at 25 ng ml^−1^ and incubated for 72 h. Ruxolitinib (MedChemExpress) was added for 72 h at 20 µM. To induce DNA damage, cells were treated with etoposide (25 µM) for 24 h as a positive control. As_2_O_3_ (10 µM) was used to disrupt PML bodies. To modulate the cell cycle, 1 µM palbociclib (G1 arrest; MedChemExpress) or 6 µM RO-3306 (G2/M arrest; Sigma-Aldrich) were used for 48 h. TAK-981 (75 nM; MedChemExpress) was used as a pan-SUMOylation inhibitor for 48 h. TAK-243 (500 nM; MedChemExpress) was used as a ubiquitination inhibitor for 48 h.

### Lentivirus production

Per reaction, 1 µg of genome vector, 800 ng of packaging plasmid (Addgene #8455; courtesy of Bob Weinberg), 200 ng of envelope plasmid (Addgene #8454; courtesy of Bob Weinberg) and 6 µl of polyethyleneimine were mixed in 200 µl Opti-MEM and incubated for 10 min at room temperature. Each mixture was then added dropwise to a well in a six-well plate containing 5 × 10^5^ plated HEK-293T cells. After incubating for 2–3 days, the supernatant was collected, passed through a 0.2-µm filter and used immediately or frozen at −80 °C. Cells were transduced in the presence of 8 µg ml^−1^ polybrene.

### In vitro transcription

ppp-RNA were produced in vitro using previously published methods^14^. In brief, overlapping oligomers containing a T7 promoter, the desired protospacer and single guide RNA (sgRNA) scaffold were amplified using Phusion polymerase (New England Biolabs). The unpurified DNA product was then subjected to in vitro transcription using the NEB HiScribe T7 High Yield RNA Synthesis Kit (New England Biolabs), incubating at 37 °C for 16 h. The following day, RNA was treated with DNase I, purified using the miRNeasy Mini Kit (Qiagen), quantified by Nanodrop and stored at −80 °C.

### Electroporation

Electroporations were conducted in 96-well format (1–2 × 10^5^ cells per well) or with 100-µl cuvettes (5 × 10^6^ cells per cuvette) using Lonza 4D electroporation kits. The kit/program for each cell type was as follows: HEK-293T (SF kit/DG-130), Jurkat (SE kit/CL-120) and RPE1 (P3 kit/EA-104). After electroporation, 80 µl of prewarmed media was added to each cuvette, and cells were allowed to sit at room temperature for 10 min before transferring to 2 ml medium in a six-well plate. For electroporation of Cas9–sgRNA to generate KOs, 100 pmol of Cas9 was complexed to 100 pmol of sgRNA and incubated for 10 min at room temperature, then added to the cells/electroporation buffer mixture. For transfection of ppp-RNA, 50 pmol of in-vitro-transcribed RNA per 1 × 10^5^ cells was added to the cells/electroporation buffer mixture.

### CRISPRi screen

RPE1 *TP53*^−/−^ WT or *SP110*^−/−^ cells stably expressing dCas9–blue fluorescent protein (BFP)–KRAB were transduced with a lentiviral CRISPRi sgRNA library^59^ containing ~104,000 elements (5 sgRNAs per gene + non-targeting controls) at a target multiplicity of infection of 0.3 in the presence of 8 µg ml^−1^ polybrene. The target sgRNA coverage was 1,000×. The lentivirus genome contained an untethered mCherry reporter gene to monitor transduction efficiency. Two days post-transduction, cells were split into two replicates and treated with 10 µg ml^−1^ puromycin for 7 days. Cells were passaged and fresh puromycin was added every 2 days. Nine days post-transduction, cells were electroporated with either in-vitro-transcribed ppp-RNA or a phosphate-buffered saline (PBS) buffer control. After roughly ten doublings (~14 days), cell pellets were collected and flash frozen. Genomic DNA was purified using the Puregene kit (Gentra), and the concentration was measured using a Nanodrop. The sgRNA cassette was amplified with NEBNext Ultra II Q5 HiFi polymerase (New England Biolabs) with indexed primers using the following steps with 5 µg of gDNA input per 50-µl reaction: 1 × 98 °C for 30 s; 22× of 98 °C for 10 s, then 65 °C for 75 s; 1 × 65 °C for 5 min. All common reactions were pooled and purified in a double solid phase reversible immobilization (SPRI) bead purification (SeraMag; Cytiva). In the first round, 0.65× SPRI beads were added, and the supernatant was collected. In the second round, 1× SPRI beads were used, and the amplicon was eluted in 20 µl H_2_O. The purified amplicons were analysed on a TapeStation DNA flow cell (Agilent) for the expected amplicon size of 270 bp. Indexed amplicons were sequenced on a NextSeq2000 P3 flow cell (Illumina) with 50-bp single end reads with a target of 50 million reads per sample. Reads were demultiplexed and analysed by both MAGeCK^60^ and DrugZ^61^, first at the sgRNA level and then at the gene level, to identify enriched and depleted genes. Raw sgRNA counts are provided in Supplementary Data 1, and the gene-level drugZ output is listed in Supplementary Data 2. GSEA (v4.3.3) was performed separately on all enriched and depleted genes, with reported gene lists having a nominative *P* value <0.05.

### Competition assay

Two populations of cells (that is, WT versus *SP110*^−/−^ transduced with a fluorescent marker protein) were equally mixed on day −1 in a six-well plate. On day 0, cells were analysed by flow cytometry and treated with the indicated conditions. Cell populations were passaged and subsequently assessed by flow cytometry for 7 days post-treatment.

### Flow cytometry

Fluorescently labelled cells (via lentiviral transduction) were analysed using an Attune NxT Flow Cytometer (Thermo Fisher), software v3.2.1. Data were analysed in FlowJo (v10.9). Live cells were gated on side scatter area (SSC-A) versus forward scatter area (FSC-A), followed by single cells on forward scatter height (FSC-H) versus FSC-A. Fluorophore presence was assessed by gating on SSC-A versus a fluorescent protein (XFP) (BFP, green fluorescent protein (GFP) or mCherry).

### Clone genotyping

Genomic DNA was isolated using QuickExtract solution (Lucigen). Reactions were incubated for 10 min at 65 °C, followed by 5 min at 98 °C and cooling to 4 °C. Primers containing Illumina adapter sequences were designed to amplify a ~200-bp region surrounding the Cas9 target sites in *SP110* and *SP100*. Genomic DNA was amplified in two rounds using NEBNext Ultra II Q5 HiFi polymerase (New England Biolabs). In PCR 1, the above designed primers were used to amplify genomic DNA in 22 cycles. One microlitre from PCR 1 was used as input for PCR 2, where Illumina indexes were added in ten cycles. Reactions were purified using 0.8× SPRI beads (SeraMag; Cytiva). Concentrations were measured on a Qubit and analysed on a TapeStation DNA flow cell (Agilent). Samples were sequenced on either a MiSeq or NextSeq2000 with 2 × 150 paired-end reads. The target read count per sample was 100,000 reads. Reads were demultiplexed and analysed with CRISPResso2^62^ (v2.0.20b) using default settings.

### Western blot

Cell lysates were collected by pelleting, washing and resuspending cells in 1× RIPA buffer supplemented with 1× protease inhibitor cocktail (Thermo Fisher). Lysates were incubated on ice for 15 min and pelleted at 15,000*g* for 15 min, and the soluble fraction was collected. Protein concentration was determined using Bradford assay. Twenty micrograms of input was electrophoresed on a 4–20% SDS–polyacrylamide gel electrophoresis gel. A wet transfer to a 0.8-µm nitrocellulose membrane was performed at 4 °C. The membrane was blocked in Tris-buffered saline + Tween 20 (TBS-T) + 5% milk for 45 min, followed by overnight incubation at 4 °C in TBS-T + 2% bovine serum albumin (BSA) containing primary antibody. The antibodies and concentrations used are found in Supplementary Table 4. The next day, membranes were washed in TBS-T and then incubated in TBS-T + 5% milk + 1:10,000 secondary antibody (LI-COR 800CW and/or 680RD). Membranes were again washed in TBS-T. A final wash in PBS was performed before imaging on a LI-COR Odyssey using the 800-nm and 700-nm channels.

### Reverse-transcription quantitative PCR

Cellular RNA was isolated using the RNeasy kit (Qiagen) with on-column DNase digestion. RNA concentration was measured by Nanodrop, and 125 ng was used as input for reverse transcription (iScript cDNA Synthesis Kit; Bio-Rad). Quantitative PCR was then performed with a 1:10 dilution of cDNA using SsoAdvanced Universal SYBR Green Supermix (Bio-Rad) on a QuantStudio 6 (Thermo Fisher) per manufacturer’s protocol. Data were then analysed via the ddCt method using *ACTB* as the endogenous control.

### T-lymphoblast viability assay

Patient- or healthy-control-derived peripheral blood mononuclear cells were expanded in 96-well plates (Axonlab) with initial stimulation using PHA (5 µg ml^−1^, Sigma-Aldrich) and human IL-2 (200 units ml^−1^, Novartis). Fresh IL-2 was added to the culture every 5–7 days. Frozen T cell blasts were used for the indicated experiments after thawing. Cells from patients with VODI, both harbouring the *SP110* c.642delC mutation^26^, or healthy donor control cells, were treated with a mock buffer control or 25 ng ml^−1^ IFNβ for 72 h. Six days post-stimulation, the relative cell viability was measured by quantifying ATP abundance (CeltTiter-Glo; Promega). The patient cohort and associated immune cell characterization have been approved by the Ethical Commission of Northwestern and Central Switzerland (EKNZ-2015-187).

### ChIP-seq

A total of 10 × 10^6^ RPE1 WT or *SP110*^−/−^ cells were incubated with 10 ng ml^−1^ IFNβ for 6 h. Cells were detached, washed, fixed in 1% paraformaldehyde (PFA) for 15 min at room temperature and quenched with 50 mM glycine. Cell pellets were washed and flash frozen in liquid nitrogen. Pellets were lysed in three sequential lysis buffers to remove cytoplasm and obtain isolated nuclei according to a previously published protocol^63^. Nuclei were sonicated with a Covaris S220 to obtain a desired fragment length of 300 bp. Lysates were then incubated overnight at 4 °C with 5 µg of prebound anti-SP100 or rabbit IgG isotype control protein A dynabeads (Thermo Fisher). The next morning, beads were washed in 6× RIPA buffer, followed by 1× TBS, and eluted in 50 mM Tris–HCl, pH 8, 1 mM EDTA, 1% SDS by incubating at 65 °C overnight to reserve crosslinks. The following day, the dynabeads were collected on a magnetic stand, and the supernatant was collected. The supernatant was then incubated with RNase A and proteinase K (Qiagen). DNA was purified (MinElute kit, Qiagen) and analysed on a TapeStation (Agilent). The NEBNextII kit (New England Biolabs) was used to prepare the sequencing library, and the indexed library was sequenced on a NovaSeq 6000 (Illumina) with 150-bp paired-end reads with a target of 25 million reads per sample. Reads were demultiplexed, aligned with Bowtie2^64^ and peaks called with MACS3^65^. Graphics were generated using deepTools^66^ and ChIPseeker^67^ (v1.32.1). Data from the comparison with Dong et al. (2022) were retrieved from SRA SRR17062883^28^.

### RNA-seq

A total of 1 × 10^6^ RPE1 WT, *SP110*^−/−^ or *SP110*^−/−^; *SP100*^−/−^ cells were electroporated ± ppp-RNA and collected 6 h later. Cellular RNA was isolated with the RNeasy kit (Qiagen) with on-column DNase digestion. RNA samples were sent to Novogene for library preparation and sequencing. The experiment was performed with three biological replicates. Reads were aligned to the genome using STAR^68^, and differentially expressed genes were annotated using DEseq2^69^. The DEseq2 results are given in Supplementary Data 3.

### Virus stocks

Low-pathogenic H5N1–GFP (recombinant influenza virus A/Vietnam/1203/2004 encoding HA without the multibasic cleavage site; a kind gift from Dr Balaji Manicassamy^70^) was propagated in MDCK cells. Virus titres, expressed as plaque-forming units (PFU) per millilitre, were determined by plaque assay as previously described^71^. VSV–GFP was a kind gift from Dr Peter Palese. HSV-1–GFP (strain C12; a kind gift from Dr Stacey Efstathiou^72^) was propagated and titrated in Vero-CCL81 cells, expressed as 50% tissue-culture infectivity dose (TCID_50_) as previously described^71^.

### Viral replication and cell death assays

A total of 2 × 10^4^ cells were seeded in a 96-well flat-bottom plate and incubated overnight at 37 °C and 5% CO_2_. Cells were inoculated with H5N1–GFP, VSV–GFP or HSV-1–GFP for 1 h at a multiplicity of infection of 10, washed once with PBS, and the medium was replaced with DMEM supplemented with 100 U ml^−1^ penicillin–streptomycin, 0.3% BSA, 0.1% FBS, 0.2 M HEPES and 1 µg ml^−1^ TPCK trypsin for H5N1–GFP, or with DMEM supplemented with 2% FBS and 100 U ml^−1^ penicillin–streptomycin for VSV–GFP and HSV-1–GFP. GFP expression (green calibrated units × µm^2^/image) and cell confluence were monitored using the Incucyte S3 live-cell analysis system (Sartorius).

### Immunofluorescence

Stably expressing H2B–GFP RPE1 WT and *SP110*^−/−^ cells were generated using lentivirus generated from Addgene plasmid #91788 (courtesy of Beverly Torok-Storb). Cells were plated on eight-well glass slides (Ibidi) and incubated in ± 10 ng IFNβ for 3 days. Cells were fixed in 4% PFA for 15 min at room temperature, washed in PBS and permeabilized in PBS + 0.1% Triton X-100 for 15 min. Following another PBS wash, cells were blocked in PBS + 2% BSA for 1 h at room temperature. Primary antibody (Supplementary Table 4) was added in PBS + 1% BSA and incubated overnight at 4 °C. The next day, cells were washed and incubated in PBS + 1% BSA with fluorophore-conjugated secondary antibody for 1 h at room temperature. Cells were washed, air dried and mounted in ProLong Diamond Mountant (Thermo Fisher). Slides were imaged on a Nikon Spinning Disk SoRa (ETHZ ScopeM) with a 100× oil-immersion objective. Images were analysed using ImageJ^73^ (v2.14.0). For 53BP1 foci and PML body quantification, images had a constant threshold applied, and then the ‘analyze particles’ function was used. Micronuclei were counted manually in the H2B channel. PML and 53BP1 Pearson object correlation was calculated using the JACoP plugin^74^.

### R-loop detection assay

Detection of RNA:DNA hybrids (R-loops) was performed using the RNaseH1^D210N^–GFP reporter assay as previously described^75^. Small interfering RNAs (siRNAs) were purchased as SMARTpools from Horizon (si*SP100*: L-015307-00-0005; si*SP110*: L-011875-00-0005). Twenty-four hours after siRNA transfection, cells were plated in 24-well plates, and expression of GFP–RNaseH1^D210N^ was induced by adding doxycycline (1 ng ml^−1^). After an additional 24 h, cells were treated with either dimethyl sulfoxide or 25 ng ml^−1^ IFNβ. CHK1 inhibitor (LY2603618) treatment was used as a positive control for the induction of R-loops. Cells were pre-extracted with CSK buffer (10 mM HEPES–NaOH pH 7.4, 100 mM NaCl, 3 mM MgCl_2_, 300 mM sucrose and 0.5% Triton X-100) for 5 min on ice before fixation in 4% PFA, and DNA was counterstained with 4′,6-diamidino-2-phenylindole (DAPI; 1 µg ml^−1^). Image acquisition was performed on an Opera Phenix spinning disc confocal microscope (PerkinElmer) at 40× magnification. GFP nuclear intensity was analysed with Harmony image analysis.

### Subcellular fractionation

Pelleted and PBS-washed RPE1 cells were resuspended in cytoplasmic lysis buffer (10 mM Tris, pH 8.0, 60 mM KCl, 300 mM sucrose, 0.25% Triton X-100 (v/v) and 1 mM EDTA) and incubated on ice for 10 min. Lysate was pelleted at 400*g* for 5 min at 4 °C. The resulting supernatant was collected as the soluble (So) fraction. The remaining nuclear pellet was washed with nuclear wash buffer (10 mM Tris, pH 8.0, 10 mM KCl, 300 mM sucrose and 1 mM EDTA) and pelleted at 400*g* for 5 min at 4 °C. The supernatant was aspirated, and the pellet was resuspended in nuclear lysis buffer (50 mM Tris, pH 8.0, 140 mM NaCl, 1% NP-40 (v/v) and 1 mM EDTA) and vortexed for 30 s. The resuspended nuclear fraction was incubated on ice for 20 min and then pelleted at 18,000*g* for 10 min at 4 °C. The resulting soluble nuclear fraction (Nu) was collected. The chromatin-containing pellet was resuspended in nuclear lysis buffer, sonicated for five duty cycles, 30 s on, 30 s off using a water bath sonicator, and pelleted at 18,000*g* for 10 min at 4 °C. The solubilized supernatant (Ch) was then collected. All fractions were stored at −20 °C.

### IP–MS

RPE1 *SP110*^−/−^ cells were stably transduced with FLAG-tagged SP110, SP110 ΔCARD or SP110 CARD and selected with puromycin for 7 days. The input for immunoprecipitation was collected from 5 × 10^6^ cells stimulated for 24 h with 10 ng ml^−1^ IFNβ. All lysis steps were performed on ice. Cells were pelleted, washed and resuspended in cytoplasm lysis buffer (10 mM Tris–HCl, pH 8, 150 mM NaCl, 320 mM sucrose, 0.5% Igepal, 0.1 mM EDTA and protease inhibitor cocktail) for 15 min. The nuclei were gently pelleted and resuspended in nuclear lysis buffer (50 mM Tris–HCl, pH 8, 500 mM NaCl, 1% Igepal, 10% glycerol, 1 U ml^−1^ benzonase, 1 mM MgCl_2_ and protease inhibitor cocktail). The nuclei were lysed by passing through a douncer 30 times. The insoluble fraction was pelleted, and the supernatant was used as input for immunoprecipitation. Anti-FLAG M2 magnetic beads (Sigma) were incubated with supernatant overnight at 4 °C, rotating. Beads were washed in 3× lysis buffer followed by 1× TBS, and then bait protein was eluted by incubating in TBS containing 150 ng µl^−1^ FLAG peptide for 10 min, shaking. Samples were submitted to the mass spectrometry facility at the Functional Genomics Center Zurich. Data were analysed using Limma^76^ using spectral counts as the input. The outputs are given in Supplementary Data 4.

### Co-IP

A total of 2 × 10^5^ HEK-293T cells per condition were transiently transfected with 1,000 ng of each plasmid in the presence of polyethyleneimine. Forty-eight hours later, cells were collected and lysed directly in nuclear lysis buffer (above). Lysates were clarified and used as input for immunoprecipitation with anti-FLAG M2 magnetic beads. Protein was eluted with 150 ng µl^−1^ FLAG peptide. The input and elution fractions were analysed by western blot.

### Recombinant protein production

Human SP110 and SP100 CARD domain cDNA sequences were cloned into pET28b backbone with an N-terminal His_6_ tag, maltose-binding protein (MBP) and TEV cleavage site (Supplementary Table 5). Each construct was transformed into BL21 Rosetta 2 (Merck) and grown in LB medium at 30 °C. At an optical density (OD)_600_ of 0.6, the cells were transferred to 18 °C for 30 min, then isopropyl β-D-1-thiogalactopyranoside was added to a final concentration of 0.5 mM to induce expression. Cells were incubated for 16 h at 18 °C, then pelleted at 4,000*g* for 30 min. The cell pellet was lysed in lysis buffer (20 mM HEPES, pH 8, 200 mM NaCl, 10% glycerol, 5 mM imidazole and 1 mM phenylmethylsulfonyl fluoride) and sonicated on ice for 8 min; 10 s on, 10 s off, 20% amplitude. The lysate was pelleted at 20,000*g* for 30 min, and the soluble fraction was collected. The soluble fraction was supplemented up to 15 mM imidazole and 500 mM NaCl and added to Ni-NTA agarose beads equilibrated in wash buffer (lysis buffer + 20 mM imidazole). The bead–lysate mixture was incubated for 1 h at 4 °C, rotating. Target protein was eluted in elution buffer (lysis buffer + 300 mM imidazole) and dialysed overnight at 4 °C in dialysis buffer (20 mM HEPES, pH 8, 150 mM NaCl and 1 mM dithiothreitol) in the presence of TEV protease to cleave off the His_6_–MBP tag. Dialysed protein was then subjected to a second Ni-NTA purification to remove the His_6_–TEV and His_6_–MBP. The flow-through fraction was collected and again dialysed overnight in dialysis buffer. The next day, the purified protein was concentrated in a 3-kDa molecular weight cut-off concentrator. Protein purity was assessed by sodium dodecyl sulfate (SDS)–polyacrylamide gel electrophoresis and size-exclusion chromatography, and 1 mg ml^−1^ aliquots were flash frozen in liquid nitrogen and stored at −80 °C.

### TEM

Quantifoil R2/2 grids coated with continuous 2-nm carbon foil were glow discharged for 45 s at 25 mA. Five microlitres of protein at 0.5 mg ml^−1^ were applied to the grid for 30 s, washed twice with water, stained for 30 s with 2% uranyl acetate and dried under an incandescent lamp. Grids were imaged on a FEI Morgagni microscope at 100 kV at 32,000× magnification. For the SP110/SP100 mixing experiment, purified aliquots of each protein were thawed and mixed at the indicated ratios for 30 min before mounting on grids.

### Cryo-EM sample preparation

Quantifoil R2/2 holey carbon copper grids (Quantifoil) were cleaned by overnight incubation in ethyl acetate and coated with 1-nm continuous carbon foil by floatation. The grids were subjected to glow discharge (15 mA for 15 s) in a Pelco easiGlow glow discharge cleaning system (Ted Pella). The climate chamber of a Vitrobot Mark IV (Thermo Fisher) was equilibrated to 4 °C at 95% humidity. Four microlitres of SP100 (crosslinked with 0.5% glutaraldehyde and containing 0.05% NP40) were applied to the grid, incubated for 30 s, blotted for 1 s and subsequently vitrified by plunging into an ethane–propane mixture (1:2) maintained at liquid nitrogen temperature (77 K).

### Cryo-EM data collection and image processing

A total of 10,995 movies were collected in counting mode on a Titan Krios cryo-electron microscope (Thermo Fisher) equipped with a K2 direct electron detector (Gatan) at a nominal magnification of 165,000×, equalling a pixel size of 0.84 Å at the specimen level. The energy filter slit width was set to 20 eV. Micrographs were exposed for 7 s and fractionated into 40 frames, with a dose rate of 8 e^−^ px^−1^ s^−1^ and an electron dose of 64 e^−^ Å^−^^2^. Data were collected at defocus set between −1 µm and −3 µm. Image processing was performed in cryoSPARC^77^. Movies were drift corrected, averaged and dose weighted with patch motion correction. Contrast transfer function (CTF) fitting was performed with Patch CTF. Filament Tracer was used for particle picking, using 70 Å filament diameter, 0.25× repeat distance and 140 Å minimum length, resulting in 3,201,771 particles. The 2,884,159 particles that remained after particle picking inspection and particle extraction (360 pixels box size binned to 96 pixels) were two-dimensionally classified into 200 classes, resulting in 362,138 particles, which were subsequently extracted without binning (box size 360 pixels). Three subsequent two-dimensional classifications resulted in 296,140 particles that were refined to a resolution of 3.5 Å with Helix Refine, using an initial model that had been generated from a subset of 74,239 particles using Helix Refine and initial helical parameters that had been determined from the initial model using Symmetry Search. The refined helical parameters of the final reconstruction were 46.4° helical twist and 19.8 Å helical rise.

### Cryo-EM model building and refinement

An initial model of the SP100 card domain was generated with AlphaFold2 structure prediction^78^ and placed into the symmetrized, sharpened map of the SP100 filament using the rigid body fitting function ‘Fit in Map’ of UCSF Chimera^79^. The atomic model was rebuilt in Coot^80^. The model was refined with application of non-crystallographic symmetry using Phenix real-space refinement^81^.

### Statistics and reproducibility

All bar graphs demonstrate the mean ± standard deviation (s.d.), except where noted. All biological replicates were performed at independent timepoints. Data with statistical tests performed are indicated in the respective figure legends. No statistical method was used to predetermine sample size. No data were excluded from the analyses except in cases of experimental failure. The experiments were not randomized. The investigators were not blinded to allocation during experiments and outcome assessment. All graphs were produced in Prism (v10.1.1; GraphPad), and figures were assembled using Affinity Designer (v1.10.8; Serif).

### Reporting summary

Further information on research design is available in the Nature Portfolio Reporting Summary linked to this article.

## Online content

Any methods, additional references, Nature Portfolio reporting summaries, source data, extended data, supplementary information, acknowledgements, peer review information; details of author contributions and competing interests; and statements of data and code availability are available at https://doi.org/10.1038/s41556-026-01916-w.

## Supplementary information

Supplementary Information Supplementary Figs. 1–3.

Reporting Summary

Supplementary Data 1 Screen sgRNA counts.

Supplementary Data 2 Screen analysis.

Supplementary Data 3 RNA-seq analysis.

Supplementary Data 4 SP110 IP–MS analysis.

Supplementary Data 5. Numerical source data for Supplementary Figure 1 and 3

Supplementary Tables Supplementary Tables 1–5.

## Source data

Source Data Figs. 1–6; Extended Data Figs. 1–9 Numerical source data.

Source Data Figs. 3 and 5; Extended Data Figs. 1–3, 5–7, 9 and 10 Unprocessed western blots.

## Data Availability

Raw sequencing data from the CRISPR screens, RNA-seq and ChIP-seq experiments are deposited in SRA BioProject PRJNA1302711. Processed data from ChIP-seq are deposited in GEO GSE304976. The SP100 structure is deposited in PDB 9TNZ and EMDB EMD-56096. Numerical source data for Supplementary Figs. 1 and 3 can be found in Supplementary Data 5. Source data are provided with this paper.

## Code availability

No custom code was used in this study. All analyses were performed with publicly available tools.
