# Supplementary material for: An SP110–SP100 axis is a critical regulator of promyelocytic leukaemia body dynamics and mitotic fidelity
Source: Nat Cell Biol. 2026 Mar 13;28(4):684–95. doi: 10.1038/s41556-026-01916-w (PMC13649554; doi:10.1038/s41556-026-01916-w)

Figure 3d

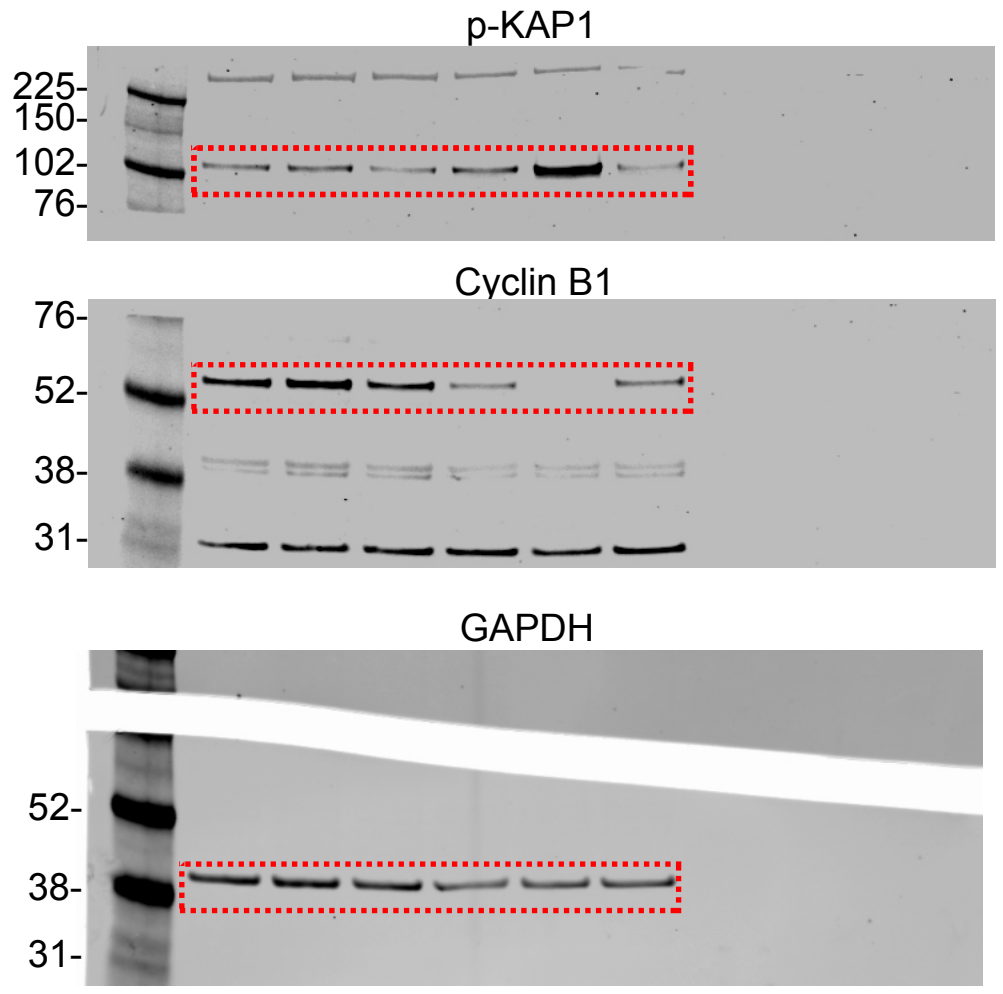

Figure 3e

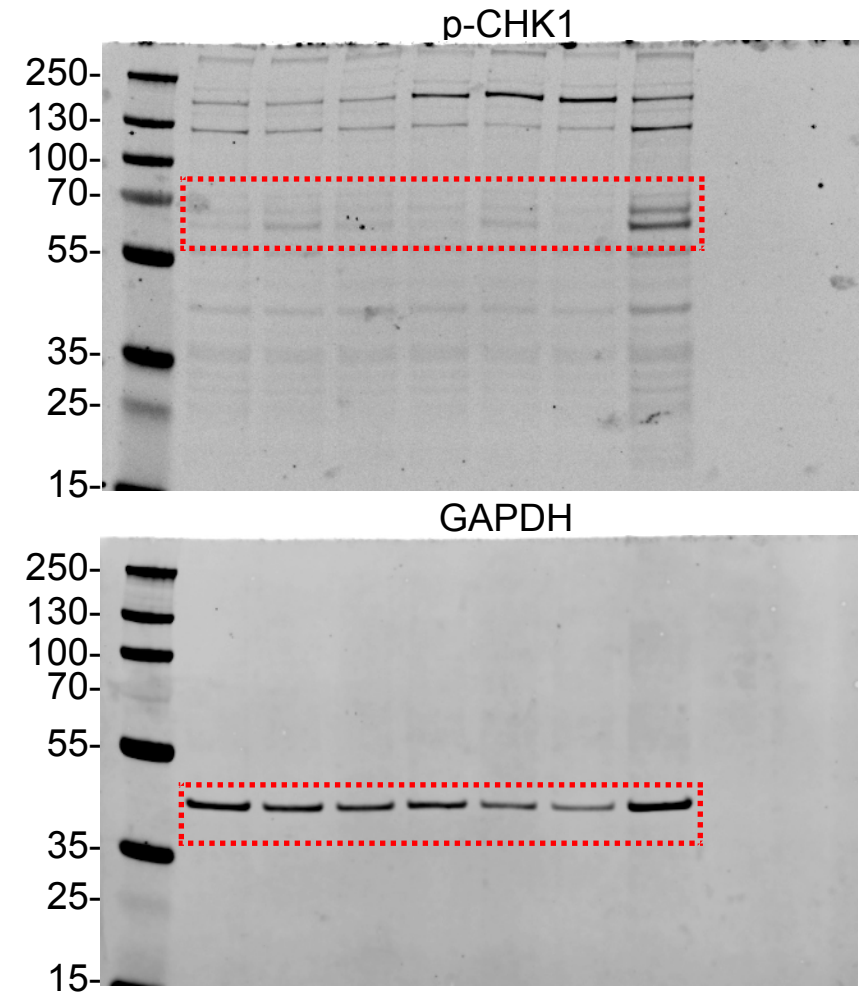

Figure 5h

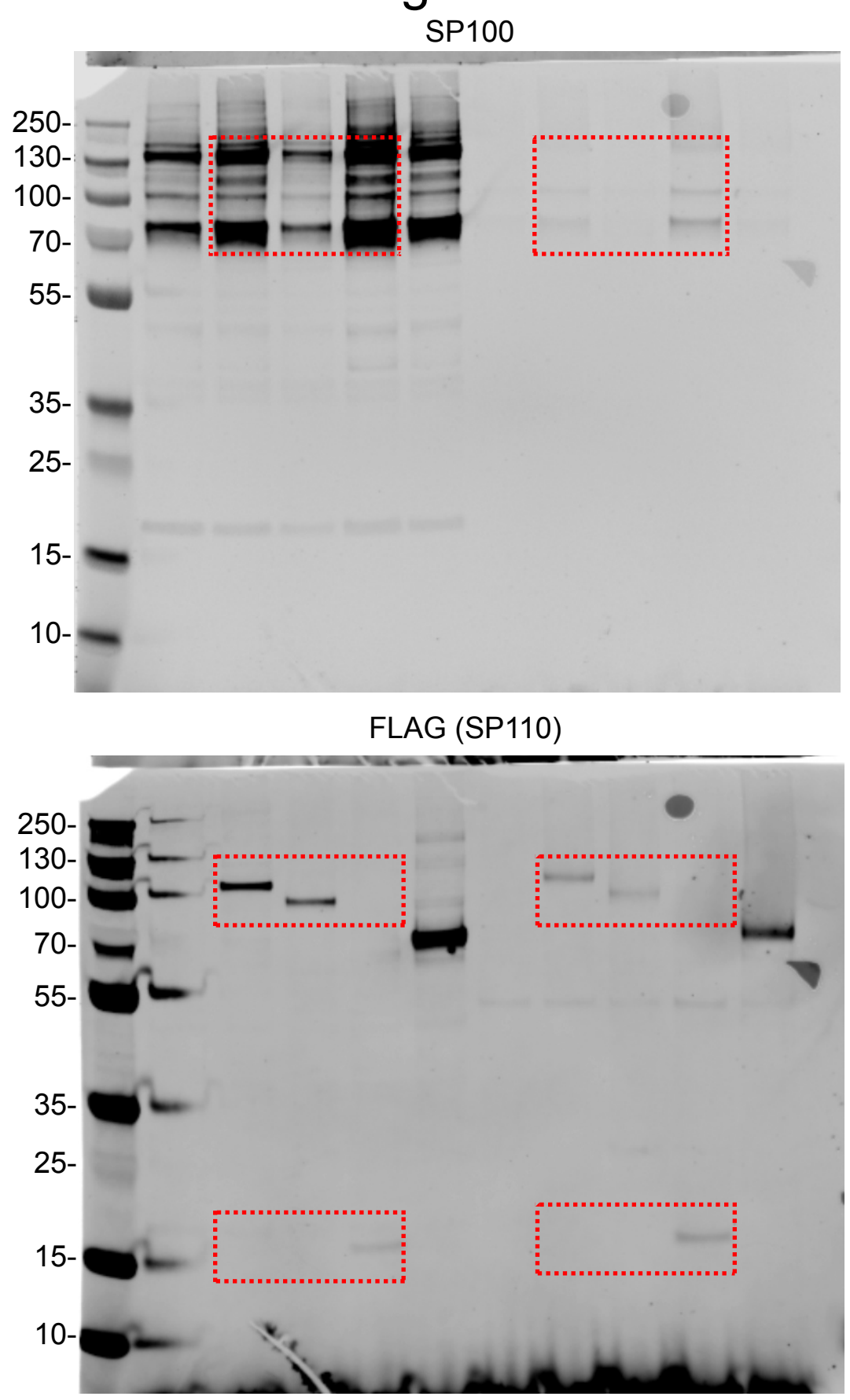

Figure 5i

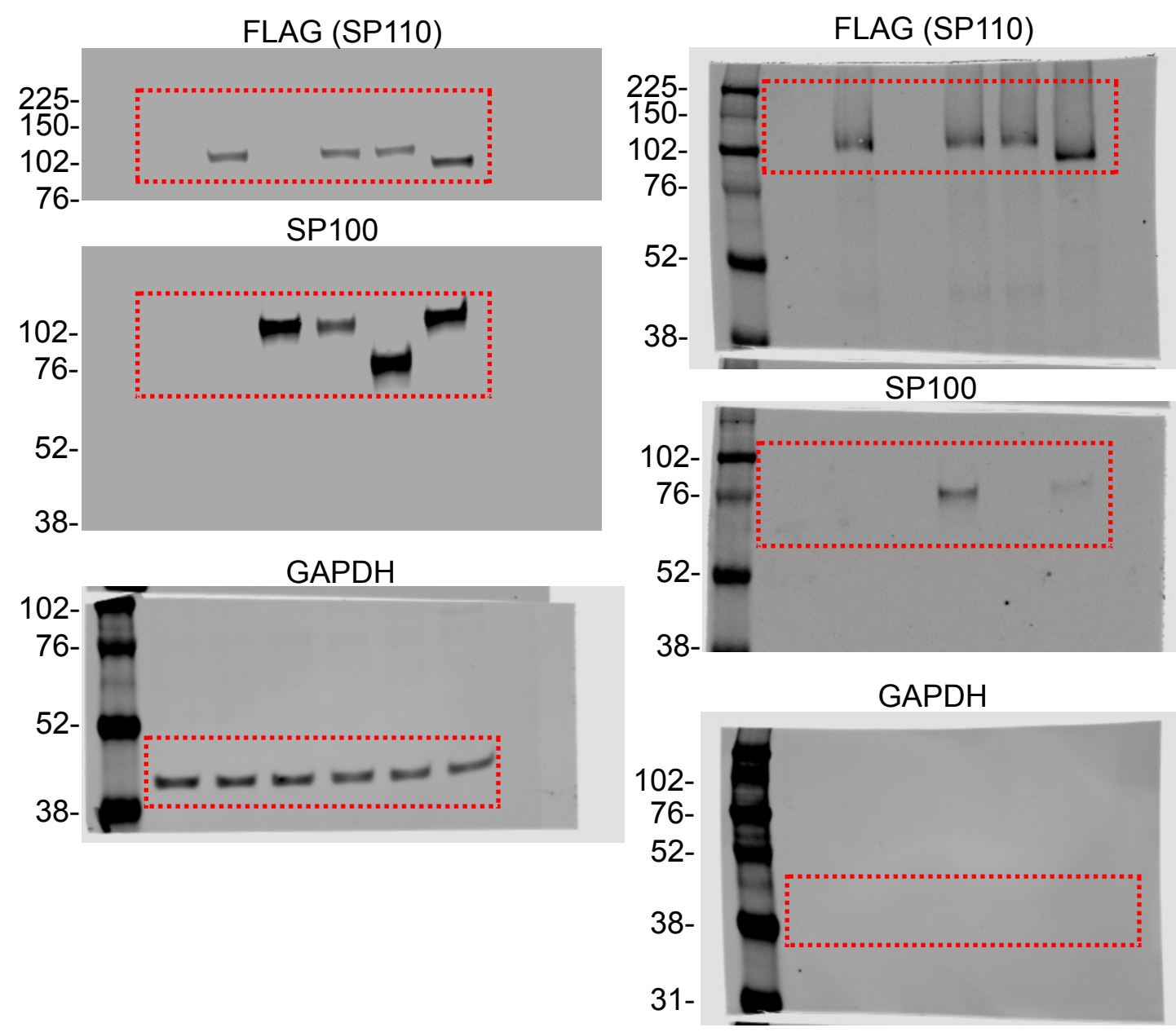

Extended Data Figure 1e

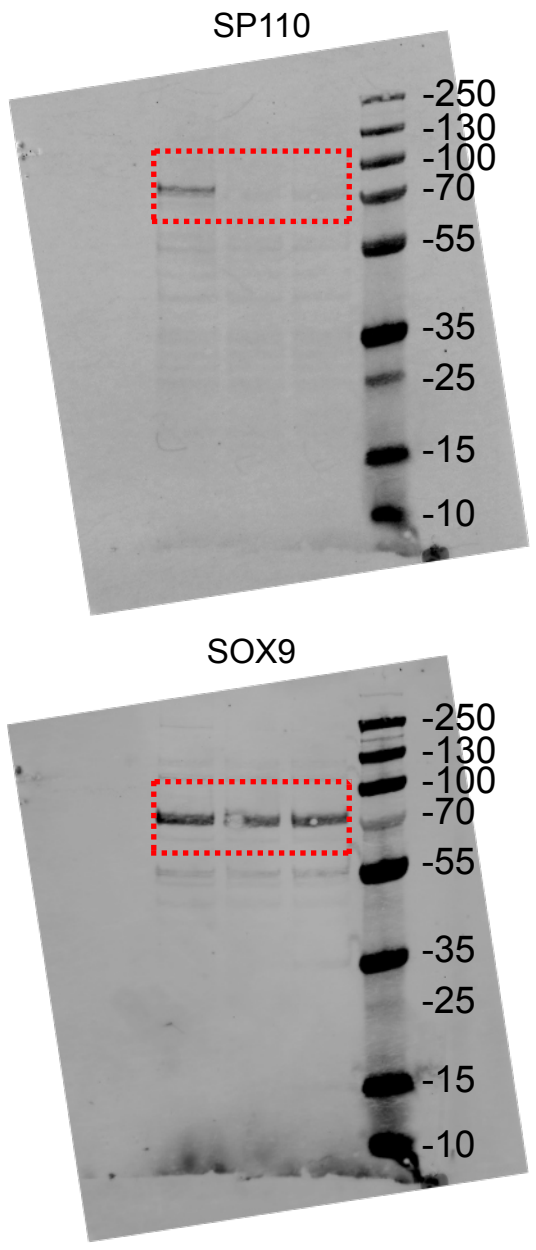

Extended Data Figure 1f

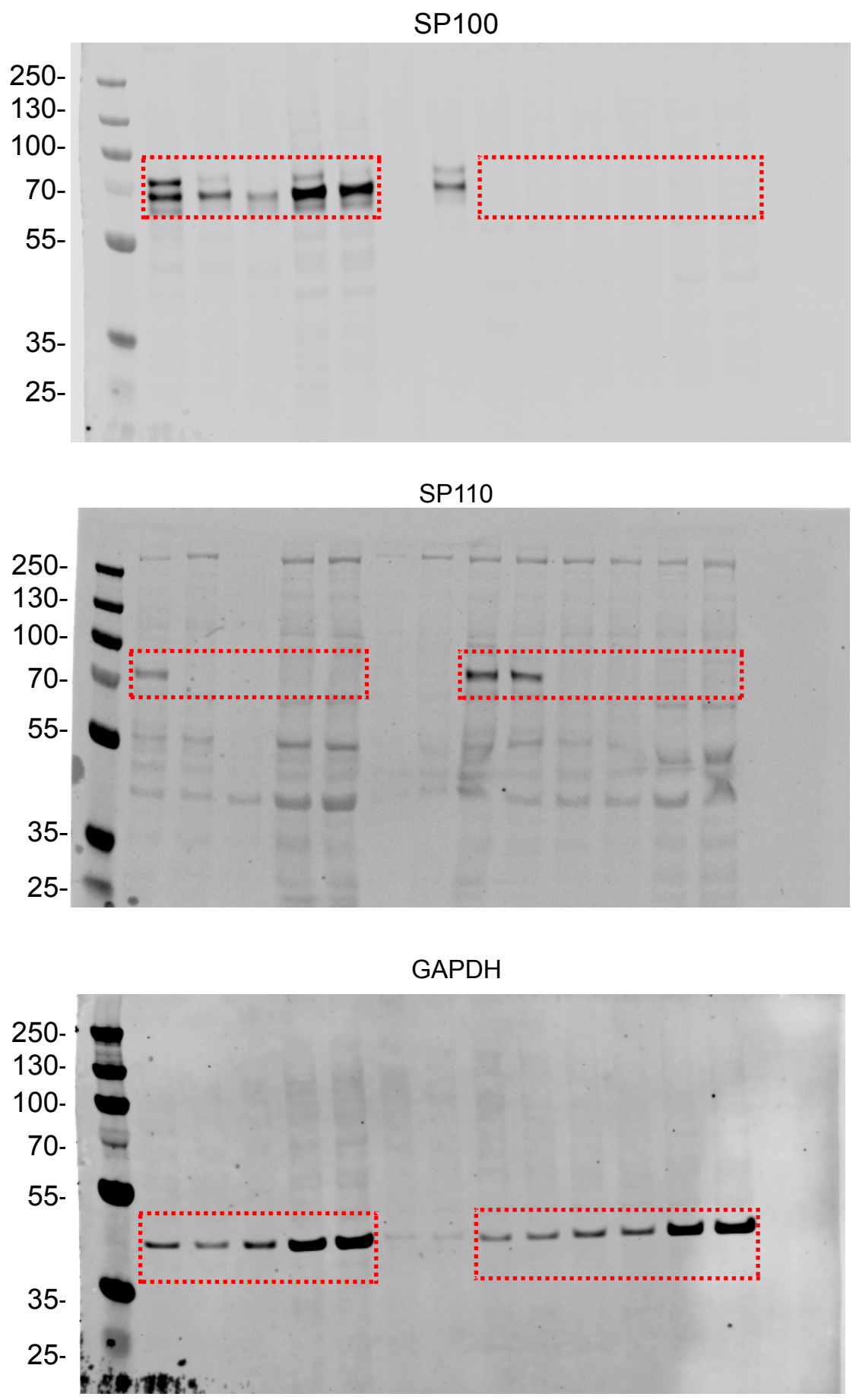

Extended Data Figure 1g

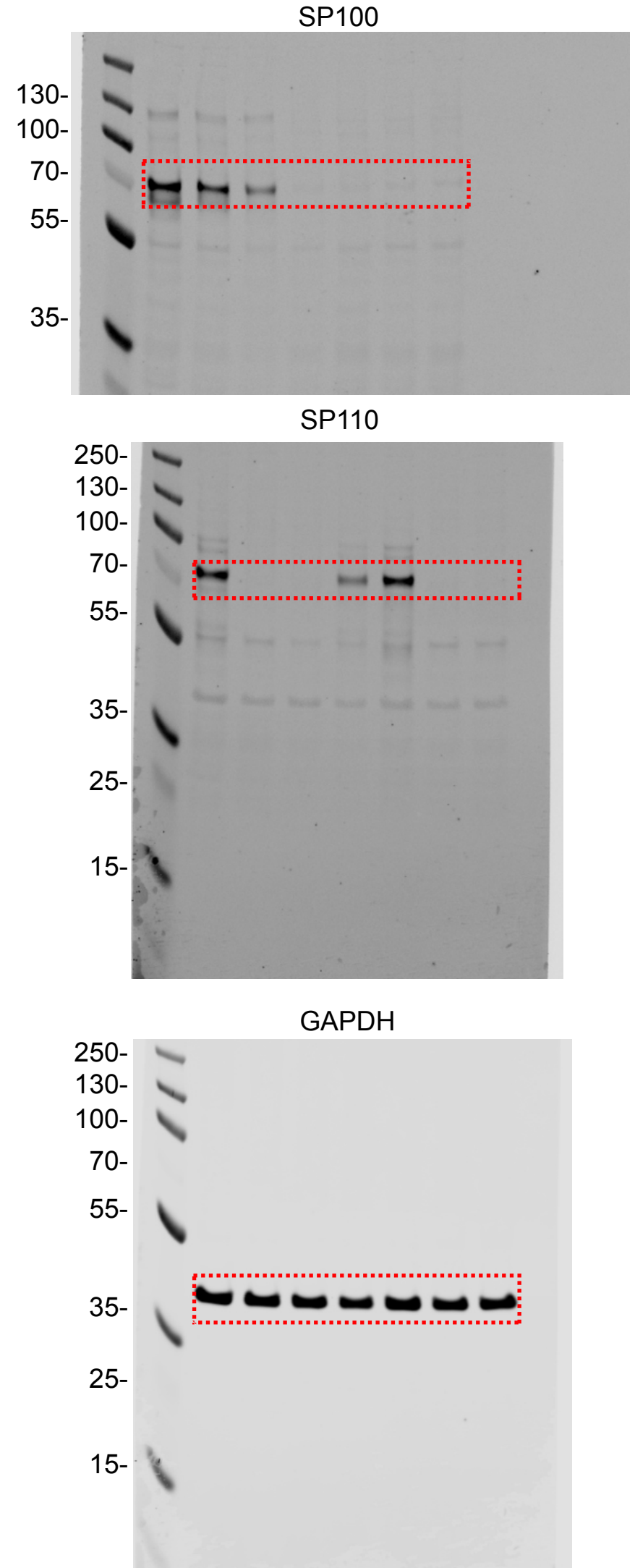

Extended Data Figure 1i

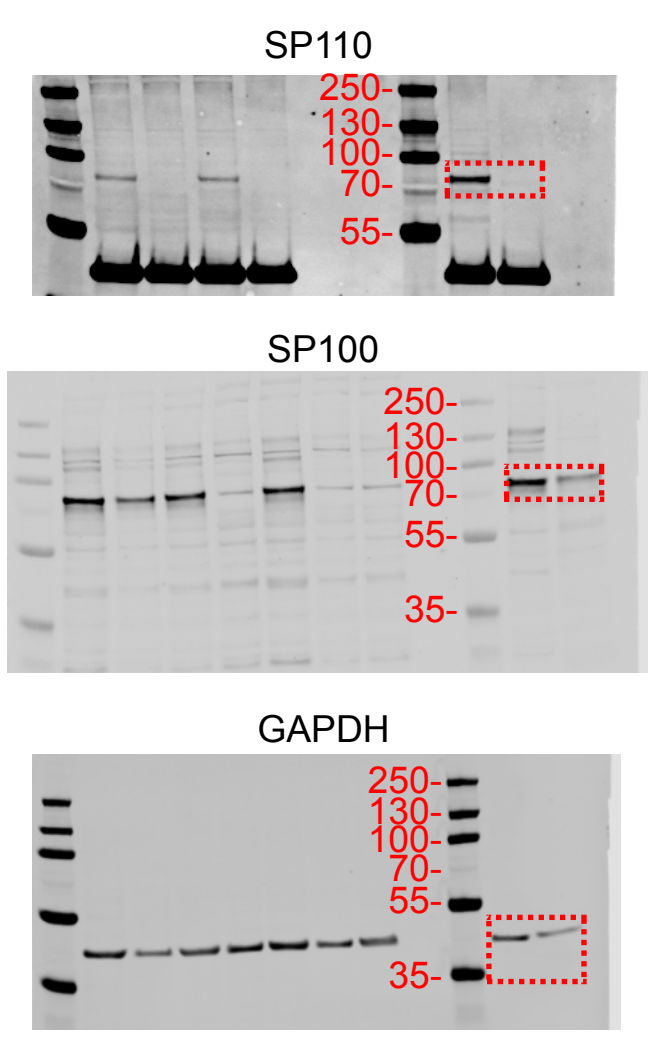

Extended Data Figure 2a

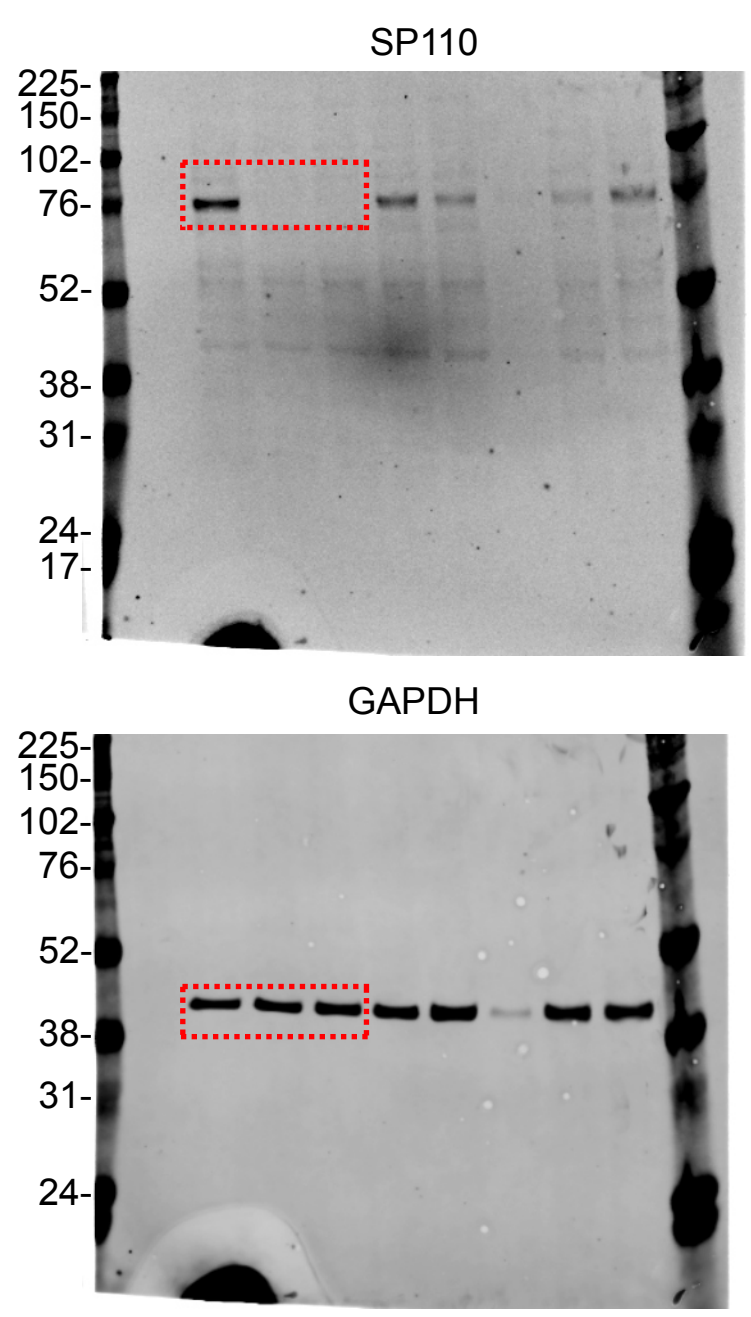

Extended Data Figure 3g

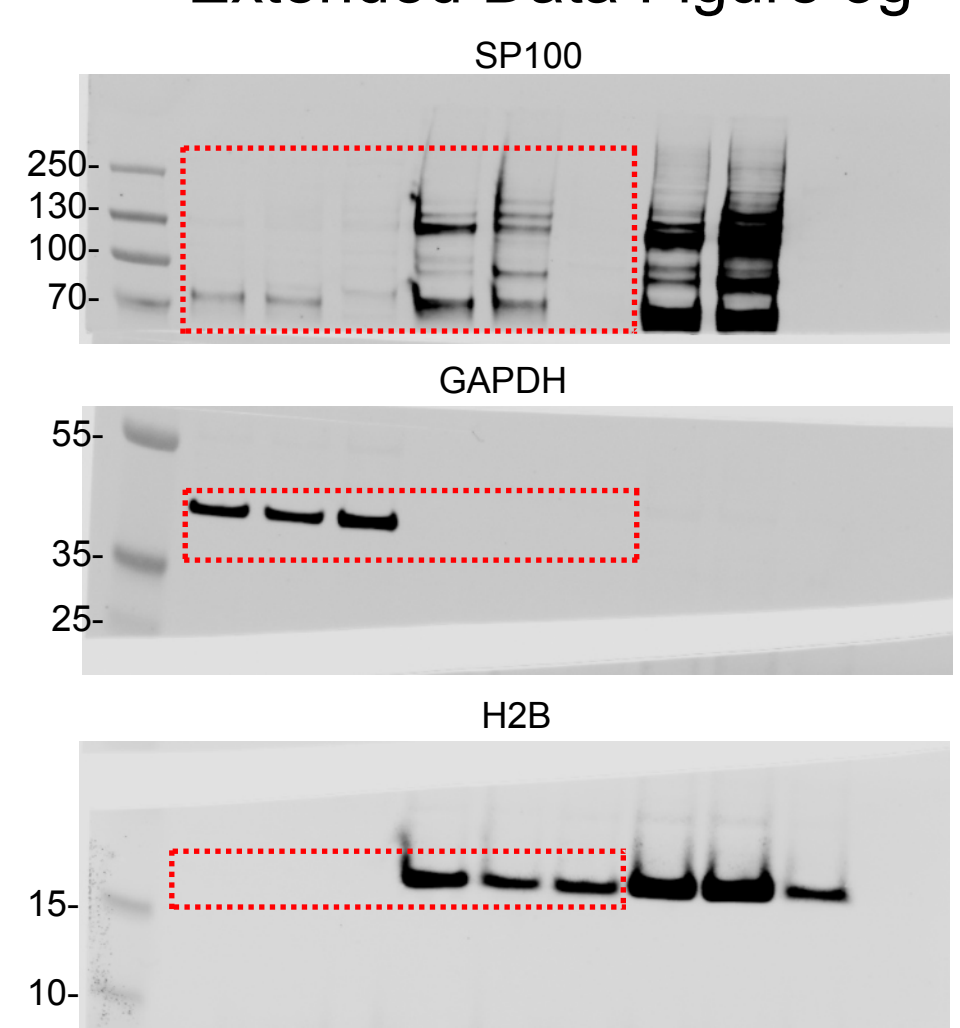

Extended Data Figure 5a

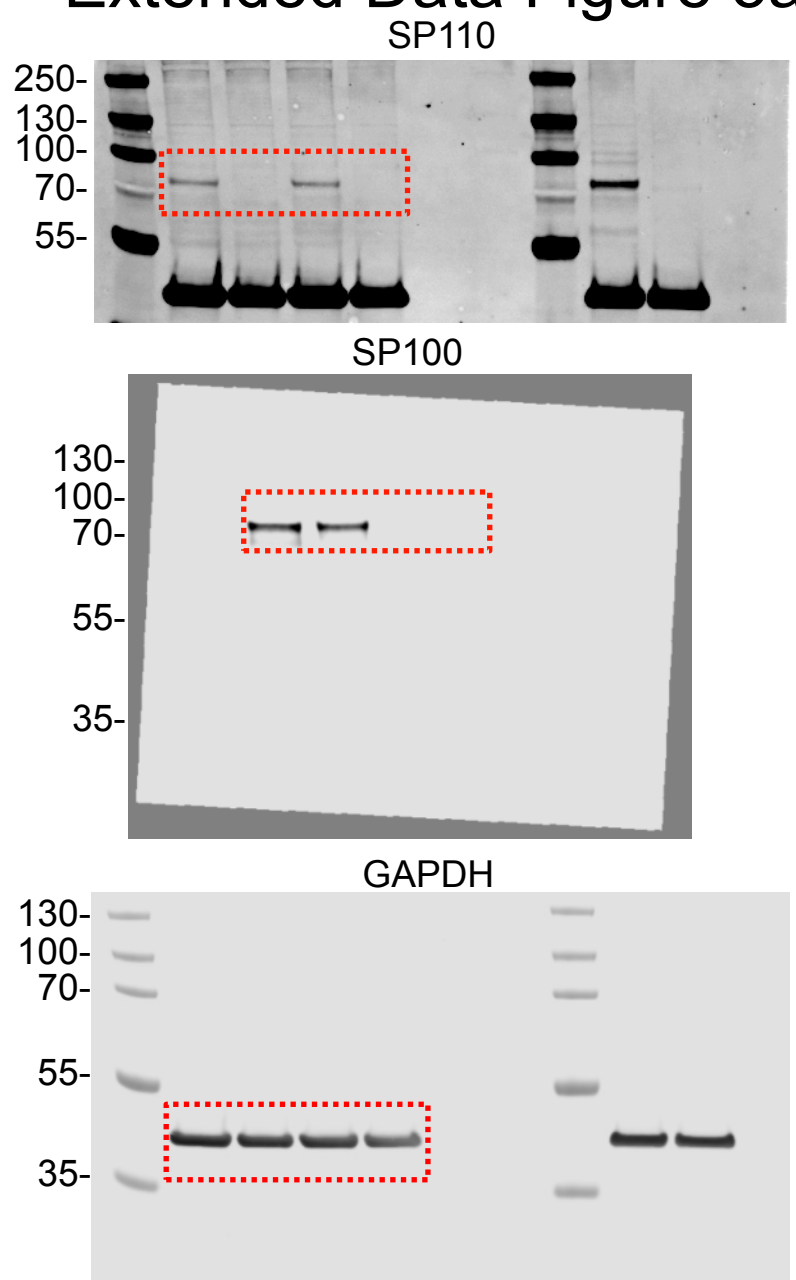

Extended Data Figure 6c

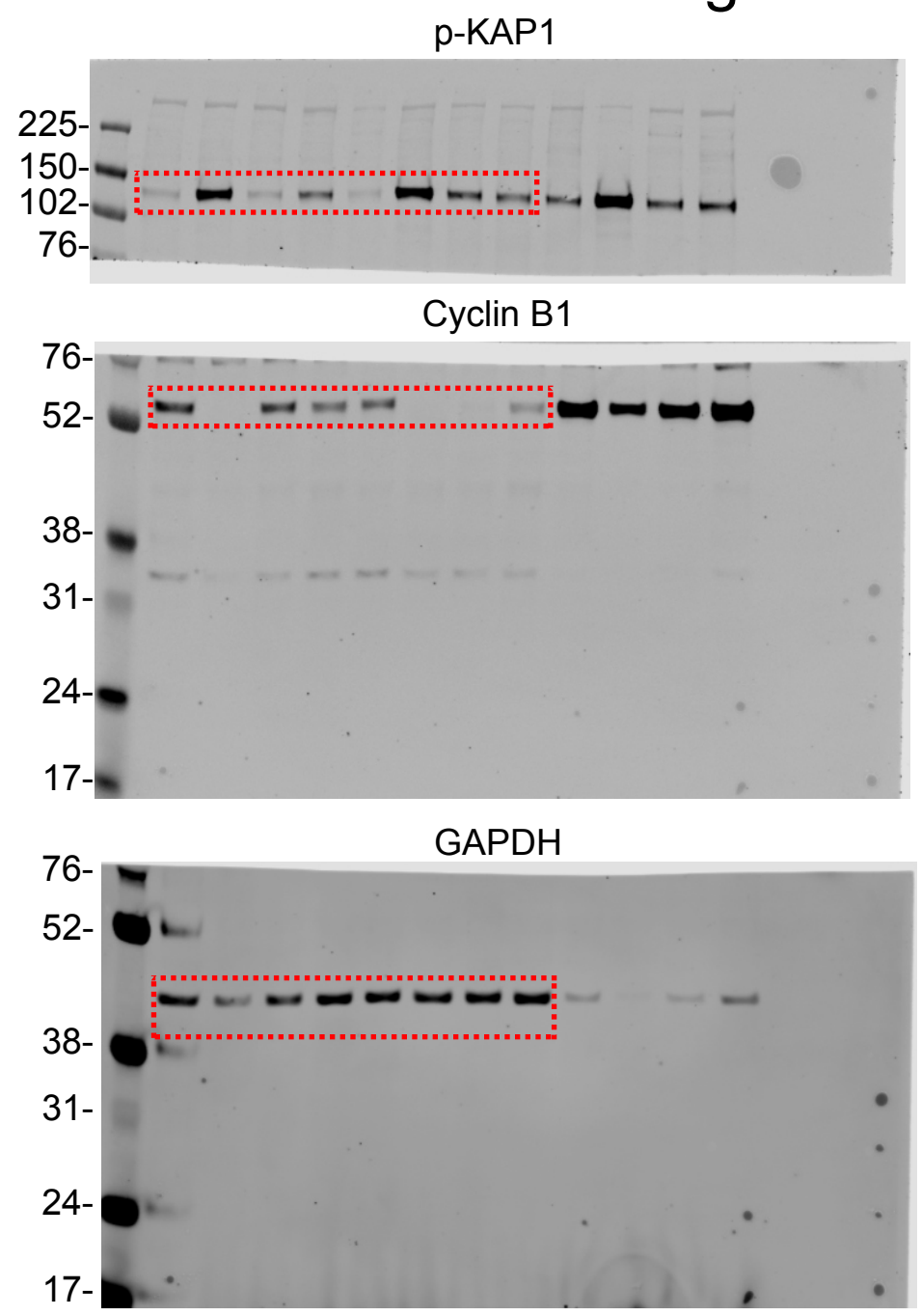

Extended Data Figure 6h

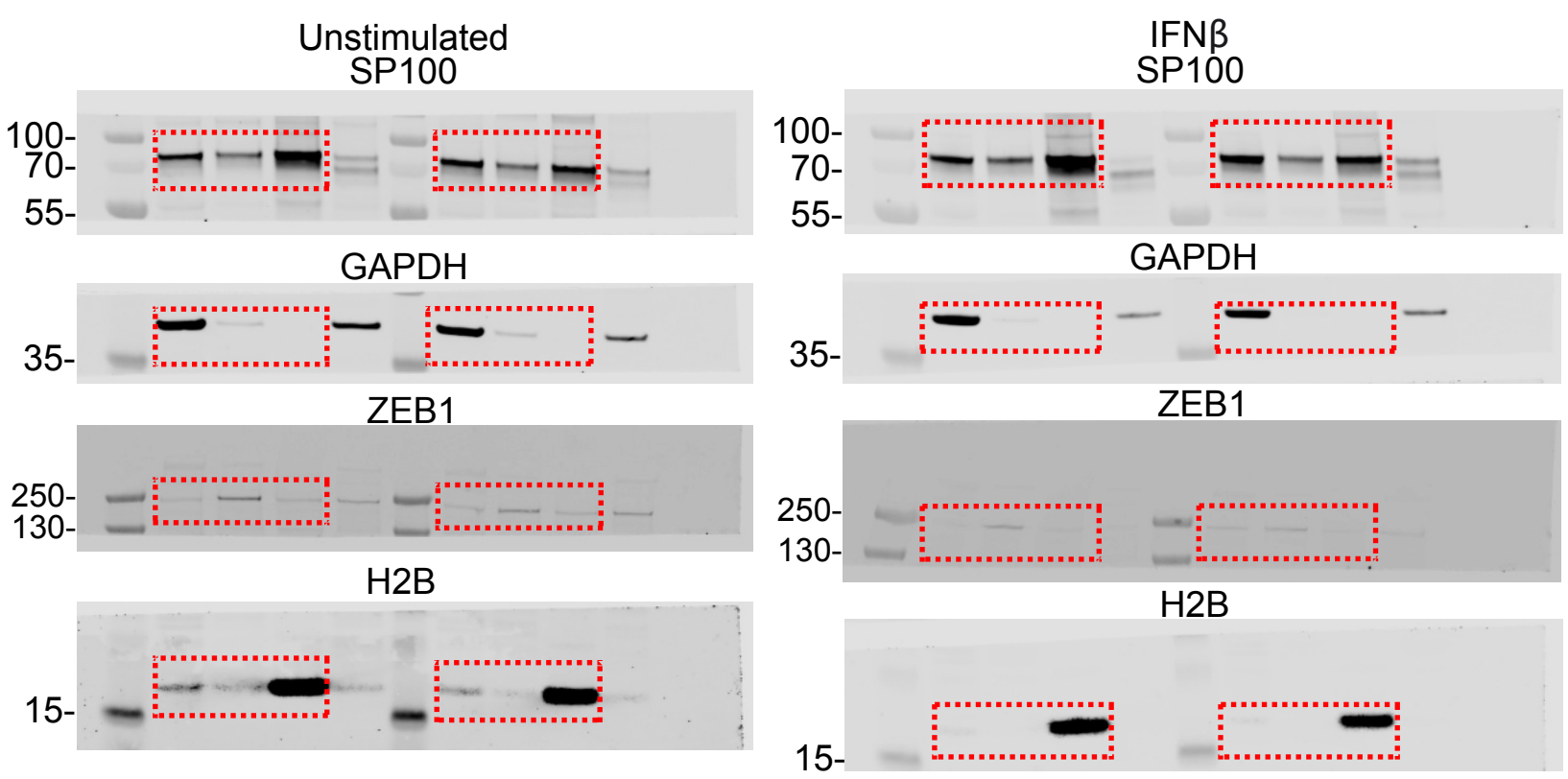

Extended Data Figure 7d

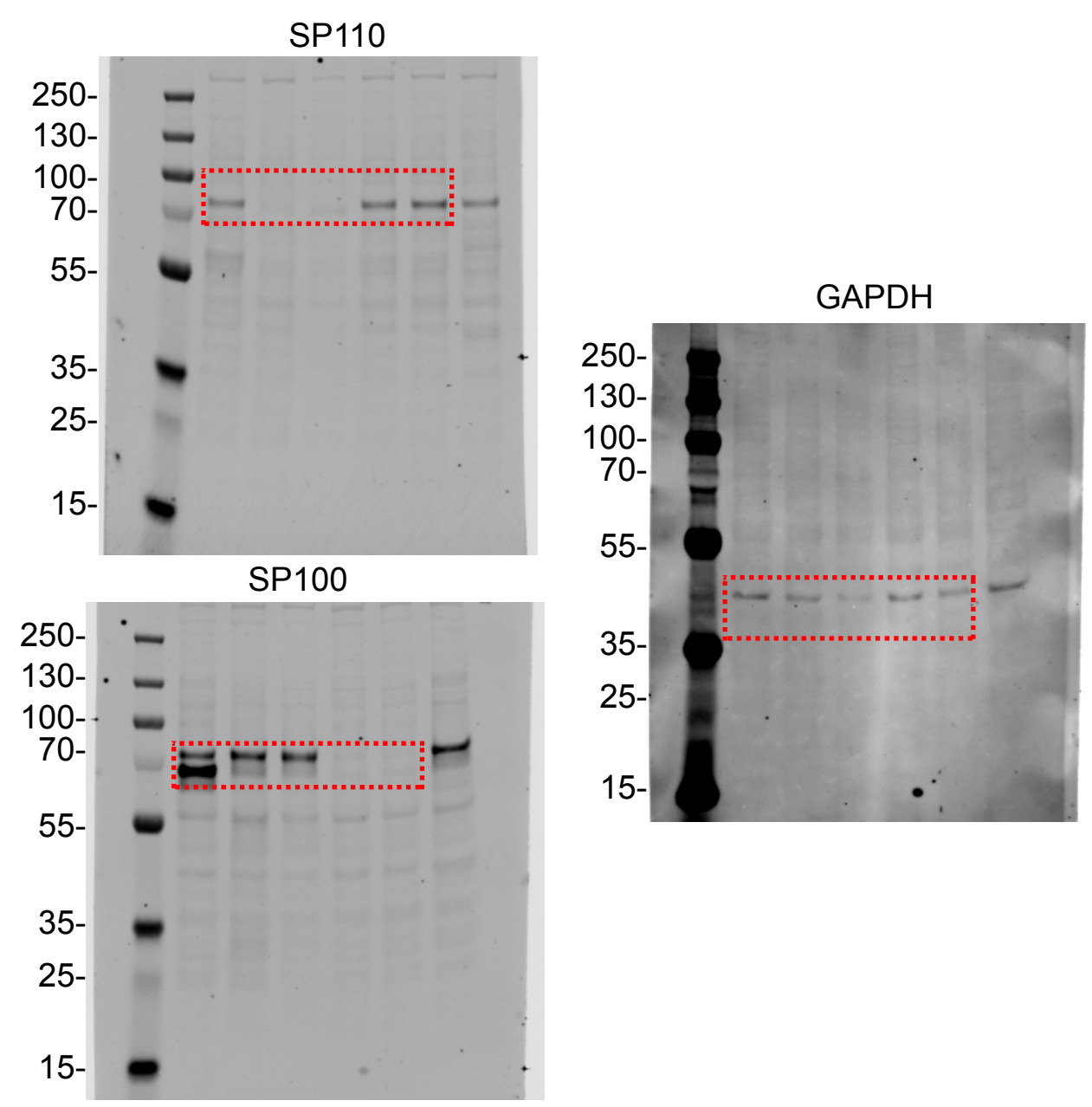

Extended Data Figure 9c

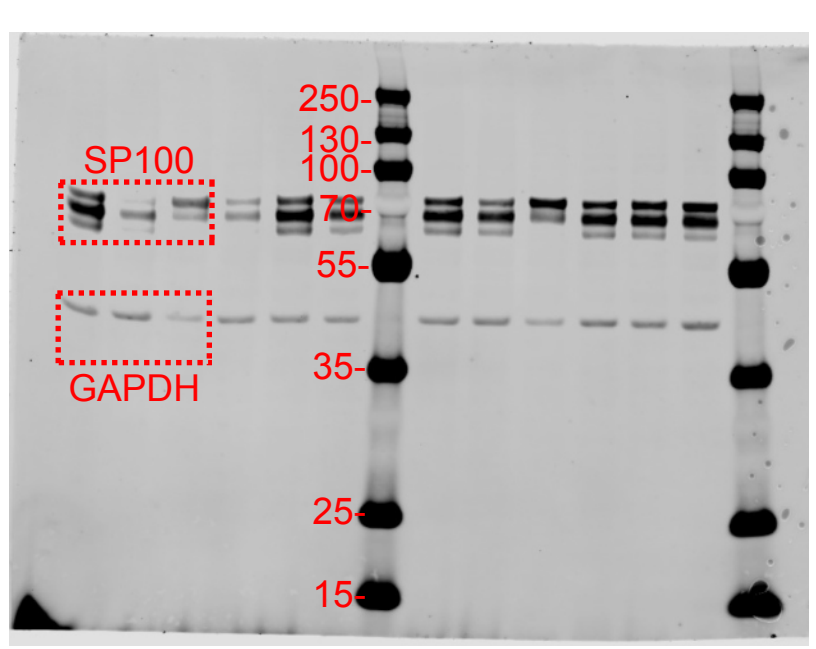

Extended Data Figure 10a

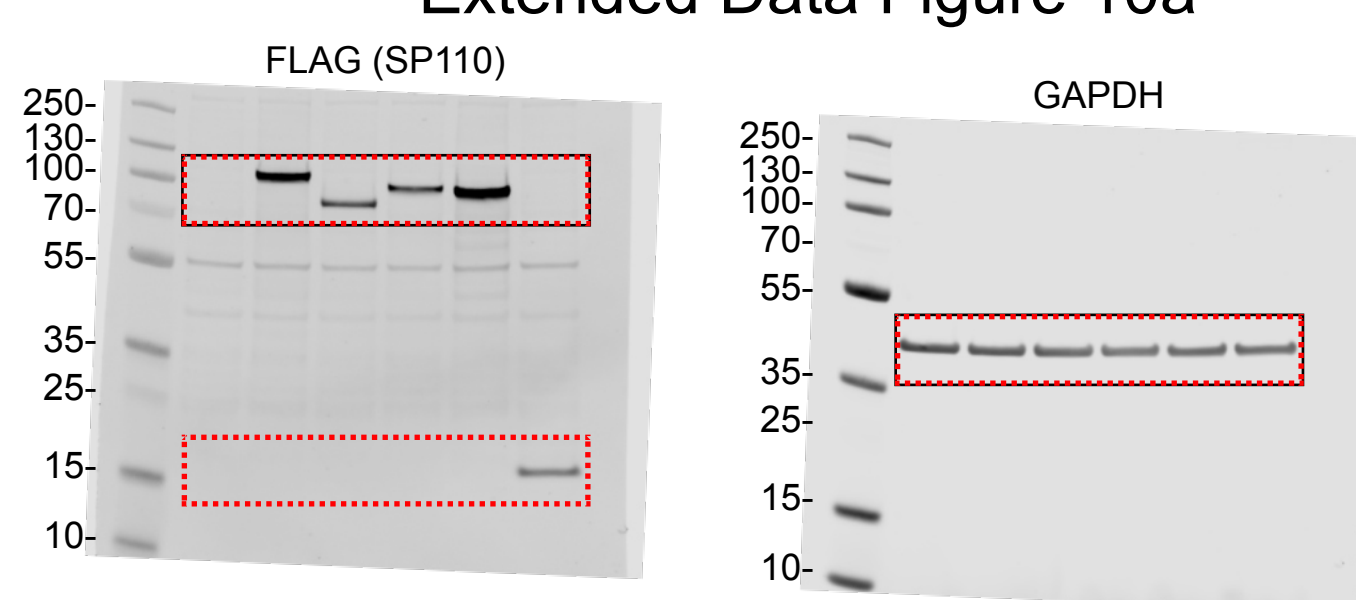

Extended Data Figure 10b

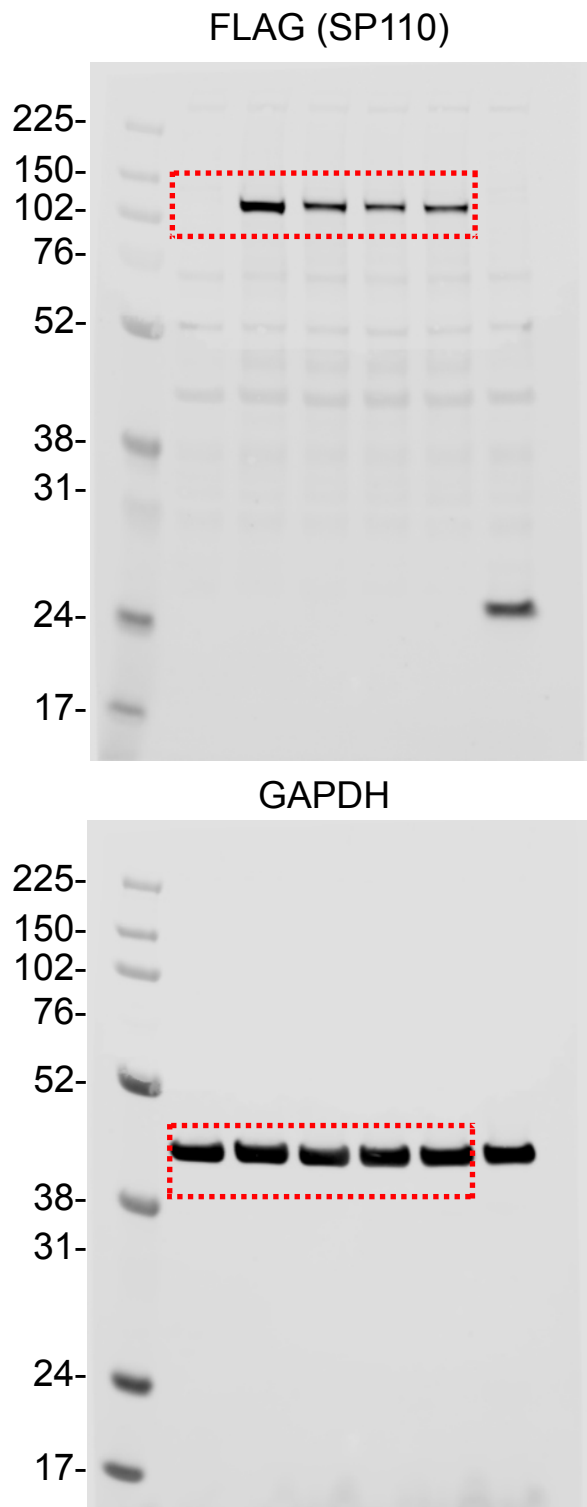

Extended Data Figure 10d

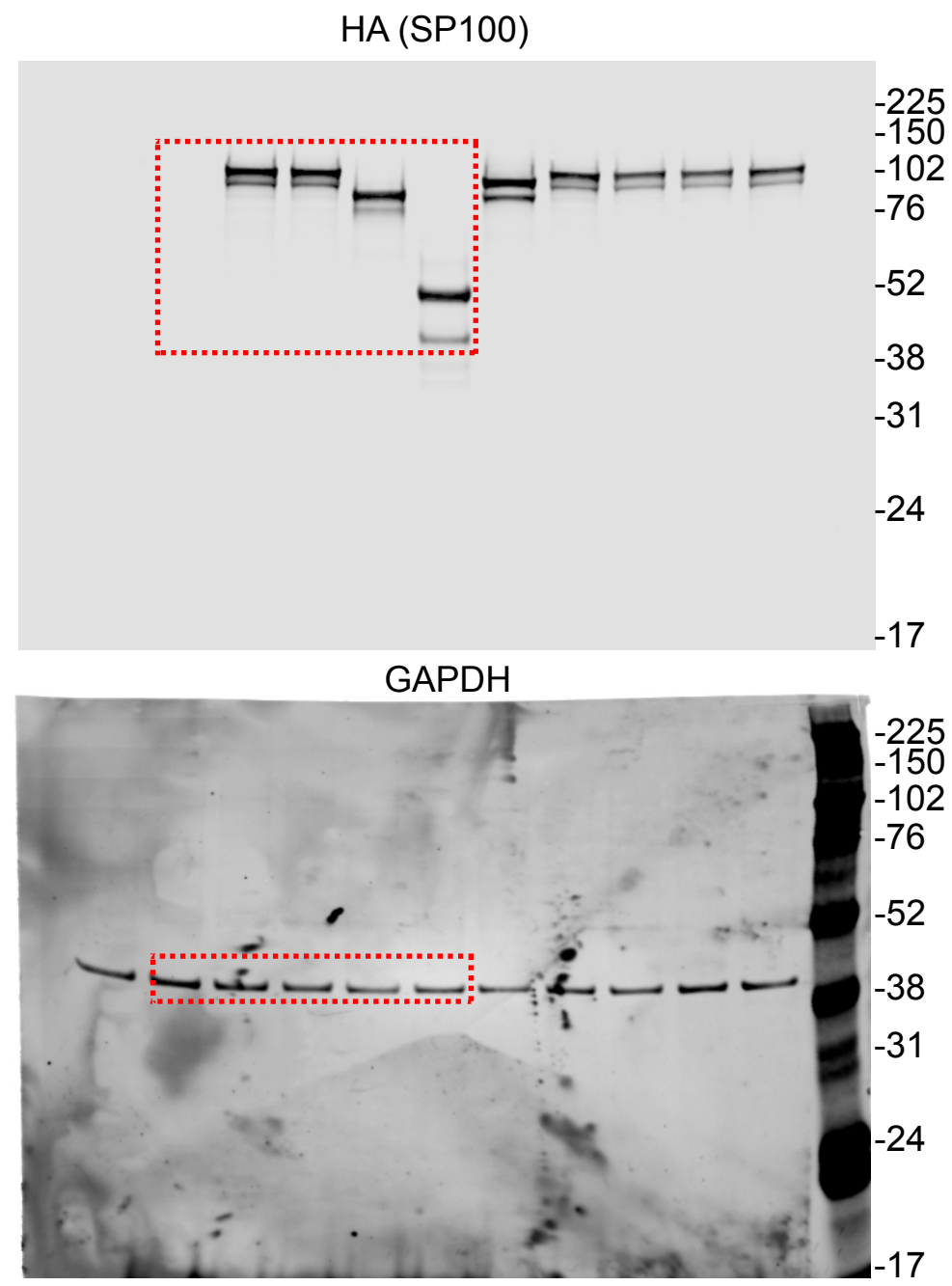

Extended Data Figure 10f

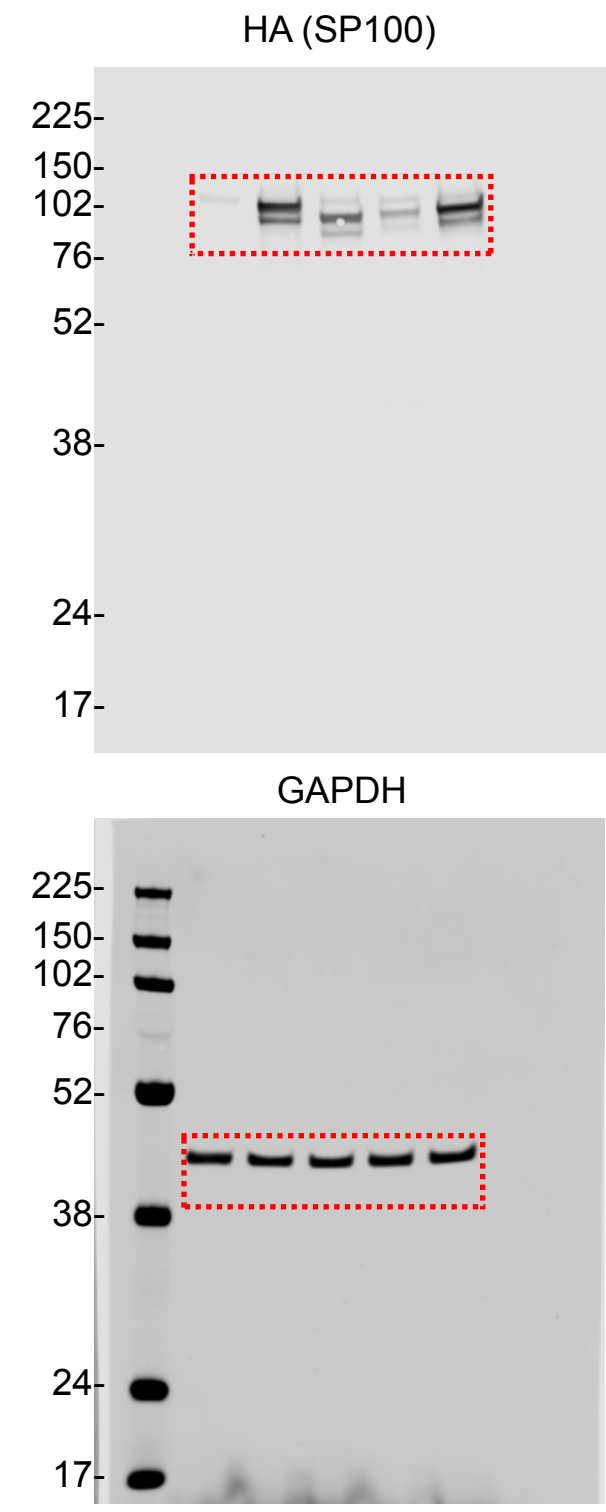

Supplement: Supplementary file 1 [file 41556_2026_1916_MOESM10_ESM.pdf]
