# Supplementary material for: An SP110–SP100 axis is a critical regulator of promyelocytic leukaemia body dynamics and mitotic fidelity
Source: Nat Cell Biol. 2026 Mar 13;28(4):684–95. doi: 10.1038/s41556-026-01916-w (PMC13649554; doi:10.1038/s41556-026-01916-w)

nature cell biology

Supplementary information

https://doi.org/10.1038/s41556-026-01916-w

An SP110–SP100 axis is a critical regulator of promyelocytic leukaemia body dynamics and mitotic fidelity

In the format provided by the authors and unedited

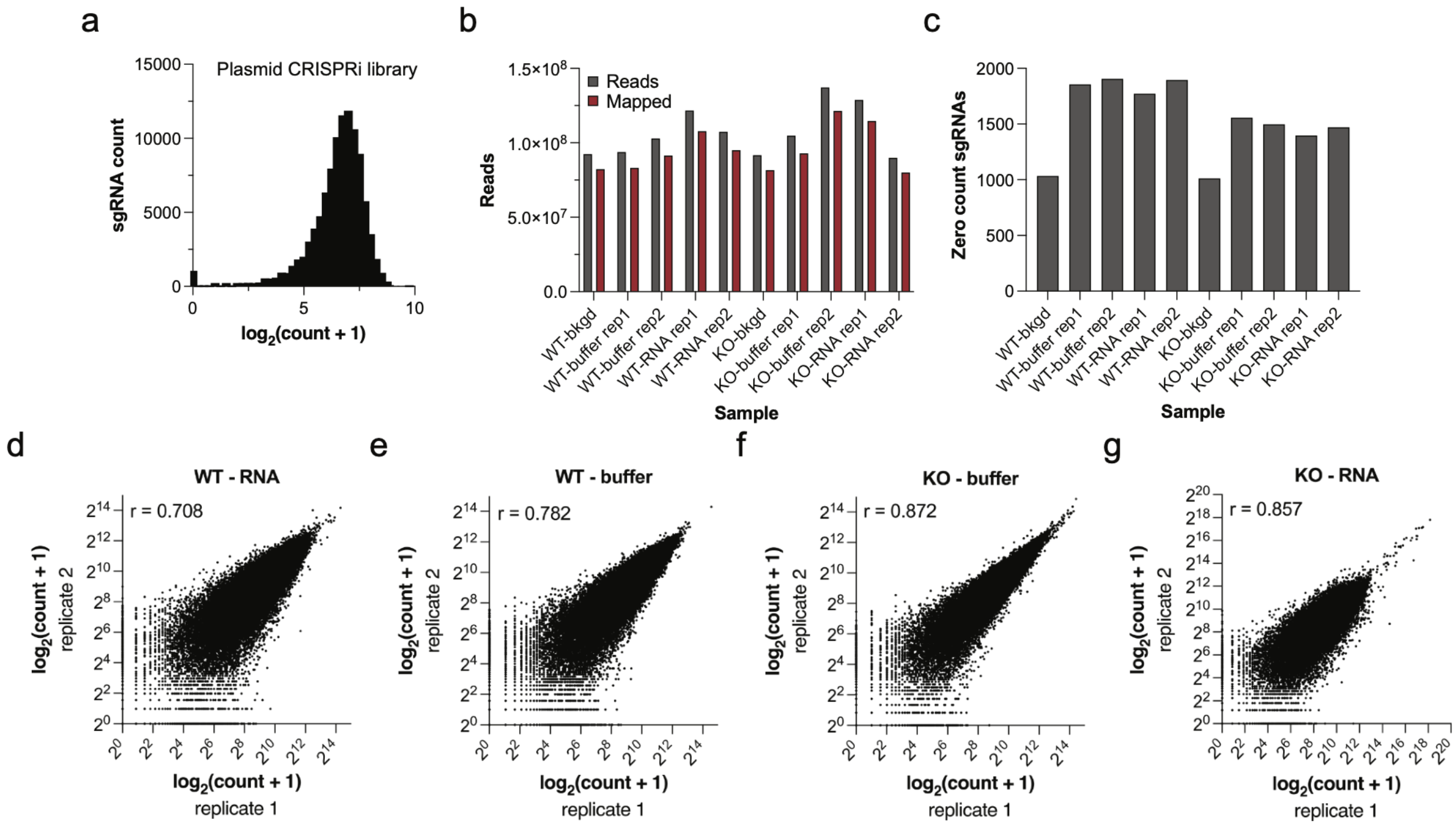

Supplementary Figure 1. CRISPR screen quality control metrics. **a**. Next-generation sequencing (NGS) $\log_2$ transformed counts of each sgRNA element of the Weissman CRISPRi-v2 plasmid library (n=104,535 elements). **b**. Quantification of total NGS read count ("Reads") and mapped reads to a library element ("Mapped") in each screen condition. WT = Screen performed in the RPE1 *TP53*$^{-/-}$ background. KO = Screen performed in *TP53*$^{-/-}$; *SP110*$^{-/-}$ background. RNA = ppp-RNA treated. **c**. Quantification of the number of sgRNA elements that had a count of zero in NGS on day 0 (bkgd) or day 14. **d**-**g**. Comparison of sgRNA element count between replicates in the indicated conditions. r = Pearson correlation coefficient. Numerical source data are available as **Supplementary Data 5**.

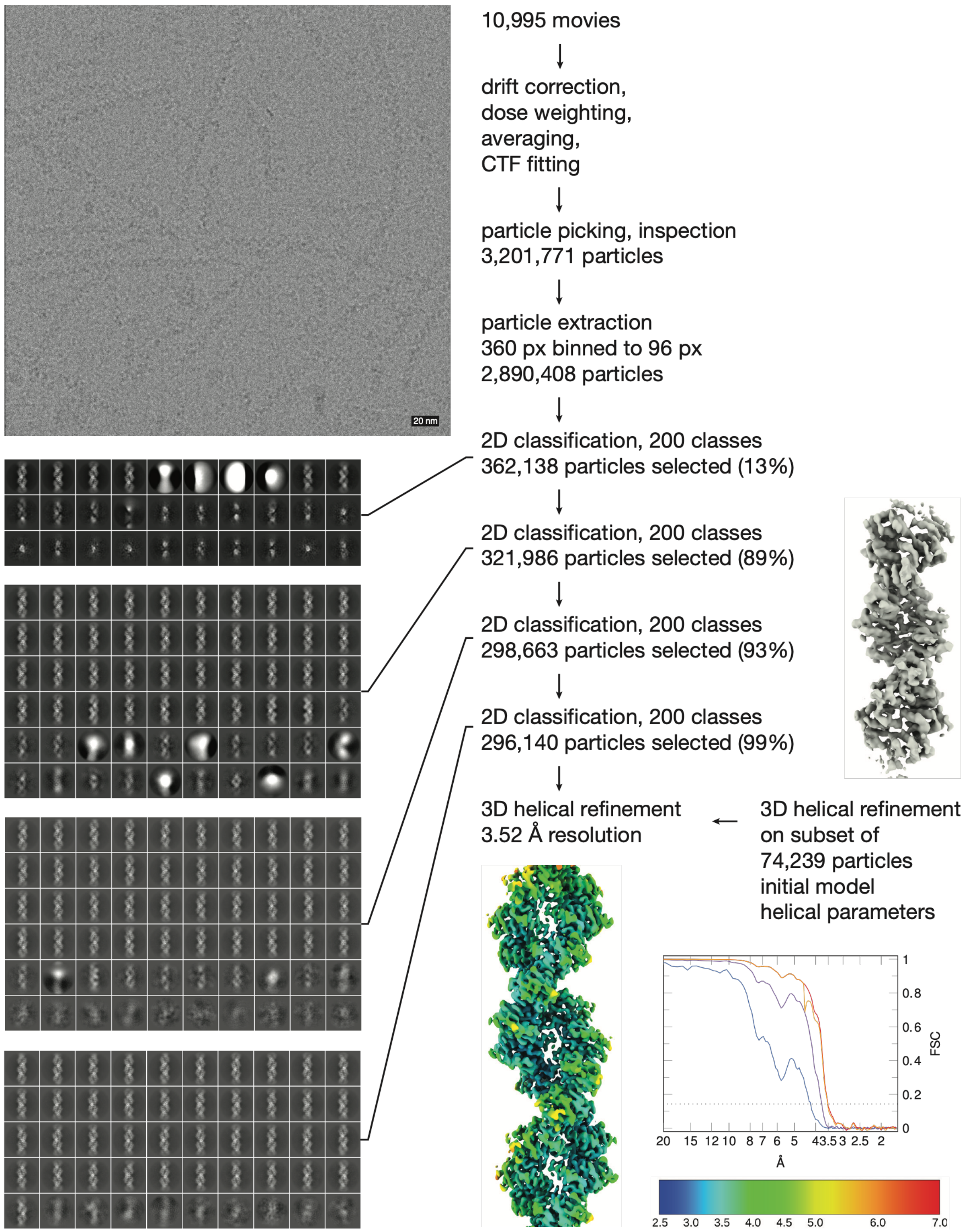

Supplementary Figure 2. **Cryo-EM image processing workflow**. Representative electron micrograph, averaged after gain correction, motion correction and dose weighting. Scale bar = 20 nm. The 2D class averages are sorted by occupancy, showing the most populated 30, 60, and 50 classes of 200 classes, respectively, with a box size of 30 nm. The FSC plot shows FSC curves without masking (blue), with loose masking (purple), tight masking (red), and corrected (yellow); correlation of 0.143 is indicated by the dotted line.

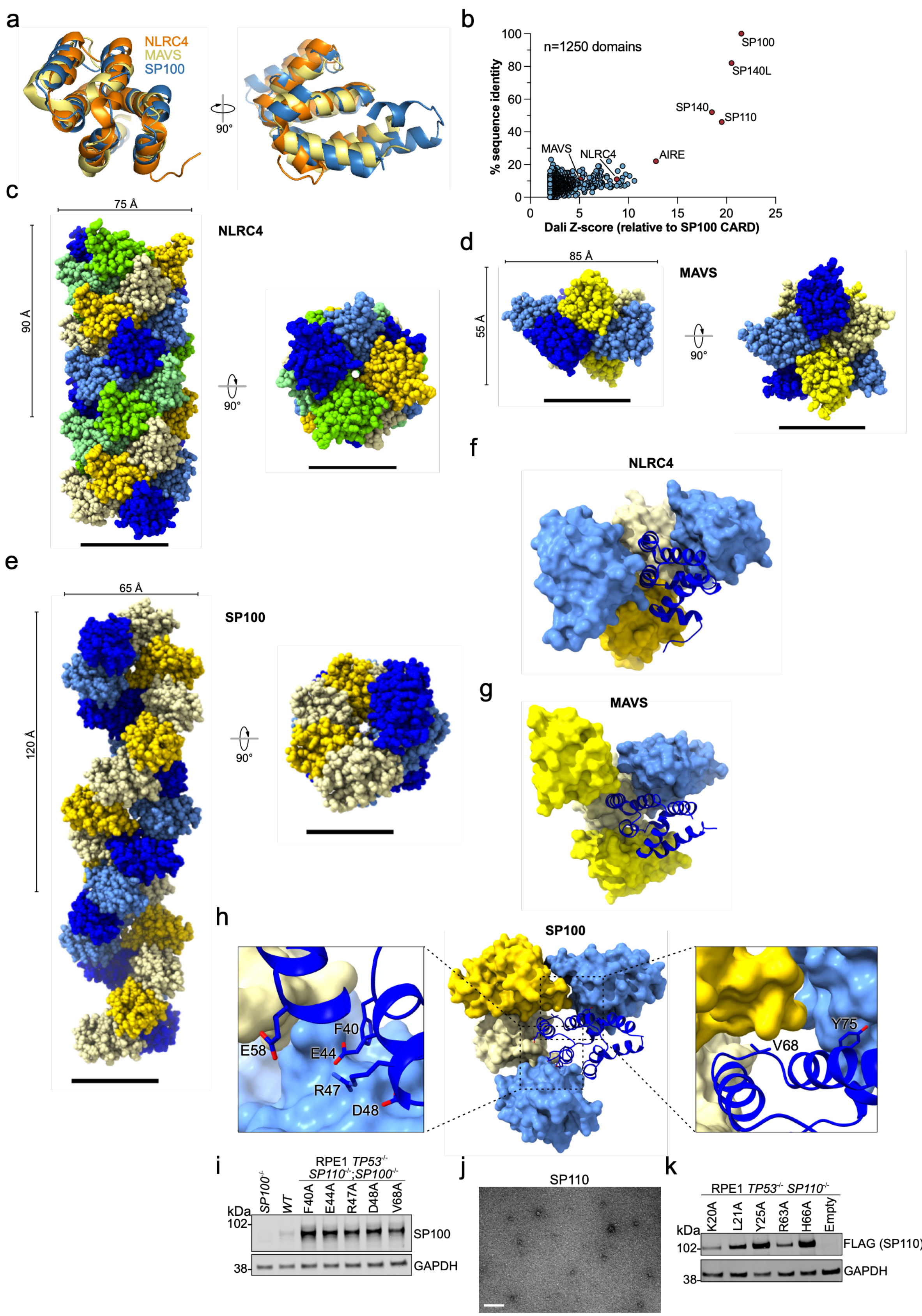
a
NLRC4
MAVS
SP100
90°
b
n=1250 domains
% sequence identity
Dali Z-score (relative to SP100 CARD)
SP100
SP140L
SP140
SP110
AIRE
MAVS
NLRC4
c
75 Å
90 Å
NLRC4
90°
d
85 Å
55 Å
MAVS
90°
e
65 Å
120 Å
SP100
90°
f
NLRC4
g
MAVS
h
SP100
E58
F40
E44
R47
D48
V68
Y75
i
RPE1 TP53-/-
SP110-/-;SP100-/-
SP100-/-
WT
F40A
E44A
R47A
D48A
V68A
kDa
102
38
SP100
GAPDH
j
SP110
k
RPE1 TP53-/- SP110-/-
K20A
L21A
Y25A
R63A
H66A
Empty
kDa
102
38
FLAG (SP110)
GAPDH

Supplementary Figure 3. **Comparison of CARD oligomers**. **a**. Overlay of CARD monomers from NLRC4 (PDB: 6MKS), MAVS (PDB: 6MKS), and SP100 (this study). **b**. Predicted structural similarity of SP100 with domains from 1250 proteins based on the Dali Z-score[44]. **c**-**e**. Cryo-EM structures of CARD oligomers (**c**) NLRC4, (**d**) MAVS, and (**e**) SP100. Scale bar = 50 Å. The top dimension corresponds to the oligomer width, and the side dimension corresponds to length of one turn of the helix. **f** and **g**. CARD binding interfaces in (**f**) NLRC4 and (**g**) MAVS. **h**. Detailed view of the locations of the engineered alanine point mutations in the SP100 CARD binding interface. **i**. Western blot of SP100 CARD point mutants stably expressed in RPE1 *SP110*;*SP100* dual KO cells. **j**. Transmission electron micrograph of recombinantly expressed SP110 CARD. Scale bar = 100 nm. **k**. Western blot of SP110 CARD point mutants stably expressed in RPE1 *SP110* KO cells. Numerical source data are available as **Supplementary Data 5**. Experiments in **i**-**k** were performed n=2-3 times independent times with similar results. Uncropped blots are available at the end of the Supplementary Information.

Uncropped blots for Supplementary Fig. 3

Supplementary Figure 3i

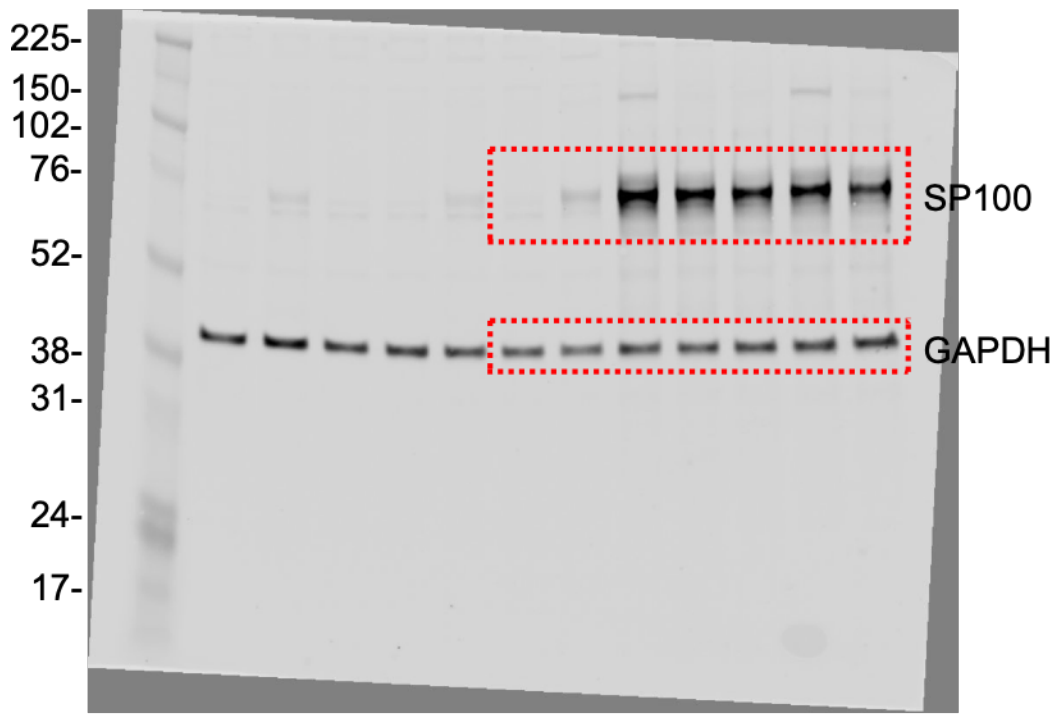

Supplementary Figure 3k

FLAG (SP110)

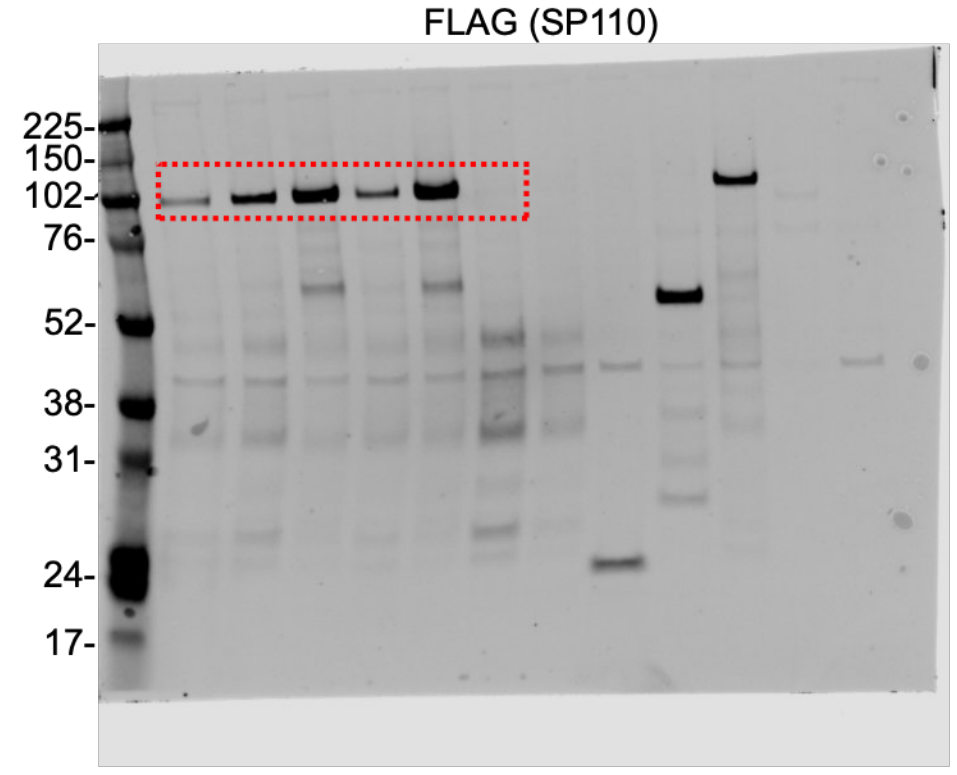

GAPDH

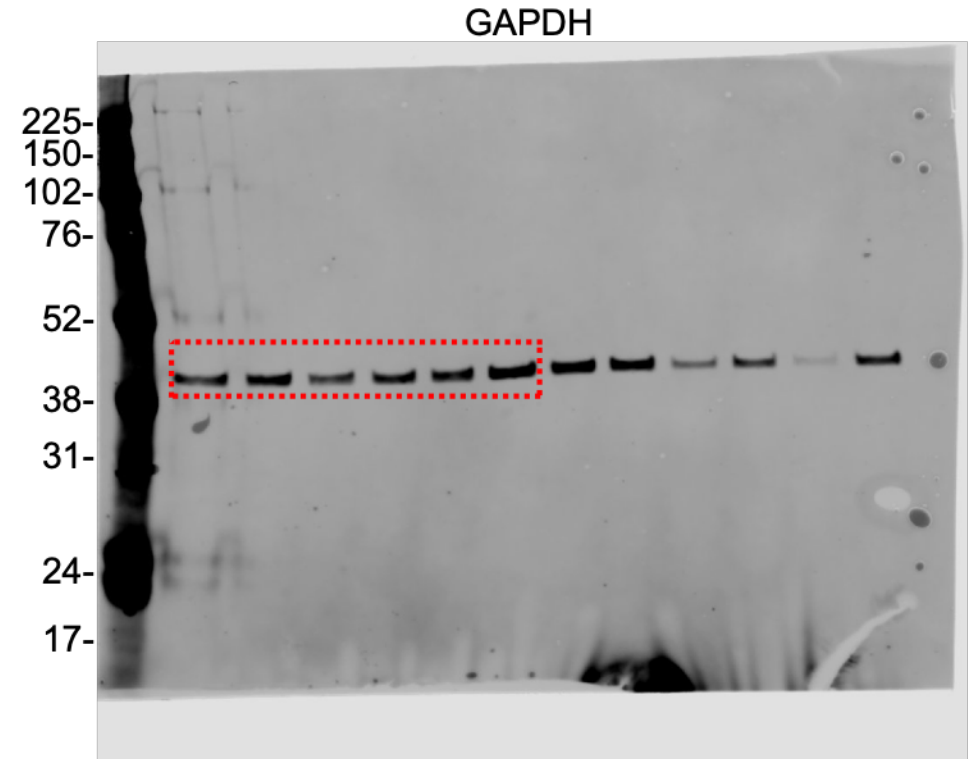

Supplement: Supplementary file 2 [file 41556_2026_1916_MOESM1_ESM.pdf]
