# Supplementary material for: An SP110–SP100 axis is a critical regulator of promyelocytic leukaemia body dynamics and mitotic fidelity
Source: Nat Cell Biol. 2026 Mar 13;28(4):684–95. doi: 10.1038/s41556-026-01916-w (PMC13649554; doi:10.1038/s41556-026-01916-w)
Supplement: Supplementary file 3 [file 41556_2026_1916_MOESM2_ESM.pdf]

nature portfolio

Corresponding author(s): Jacob E. Corn

Last updated by author(s): Feb 3, 2026

Reporting Summary

Nature Portfolio wishes to improve the reproducibility of the work that we publish. This form provides structure for consistency and transparency in reporting. For further information on Nature Portfolio policies, see our Editorial Policies and the Editorial Policy Checklist.

Statistics

For all statistical analyses, confirm that the following items are present in the figure legend, table legend, main text, or Methods section.

n/a	Confirmed	
☐	☒	The exact sample size (*n*) for each experimental group/condition, given as a discrete number and unit of measurement
☐	☒	A statement on whether measurements were taken from distinct samples or whether the same sample was measured repeatedly
☐	☒	The statistical test(s) used AND whether they are one- or two-sided<br>*Only common tests should be described solely by name; describe more complex techniques in the Methods section.*
☒	☐	A description of all covariates tested
☒	☐	A description of any assumptions or corrections, such as tests of normality and adjustment for multiple comparisons
☐	☒	A full description of the statistical parameters including central tendency (e.g. means) or other basic estimates (e.g. regression coefficient) AND variation (e.g. standard deviation) or associated estimates of uncertainty (e.g. confidence intervals)
☐	☒	For null hypothesis testing, the test statistic (e.g. *F*, *t*, *r*) with confidence intervals, effect sizes, degrees of freedom and *P* value noted<br>*Give P values as exact values whenever suitable.*
☒	☐	For Bayesian analysis, information on the choice of priors and Markov chain Monte Carlo settings
☒	☐	For hierarchical and complex designs, identification of the appropriate level for tests and full reporting of outcomes
☐	☒	Estimates of effect sizes (e.g. Cohen's *d*, Pearson's *r*), indicating how they were calculated

Our web collection on statistics for biologists contains articles on many of the points above.

Software and code

Policy information about availability of computer code

Data collection	AlphaFold3 was used to acquire predicted heterodimer structure of SP110-SP100 CARDs.
Data analysis	All graphs were produced in Prism (v10.1.1; GraphPad), and figures were assembled using Affinity Designer (v1.10.8). CRISPR screen data was analyzed using MAGeCK (v0.5.9.5) and drugZ (v1.1.0.2). Clone genotypes were analyzed using CRISPResso2 (v2.0.20b). RNA-seq data was aligned using STAR (v2) and analyzed using DEseq2 (1.40.2). IP-MS data was analyzed using Limma (v3.58.1). Microscopy data was visualized using ImageJ (v2.14.0). Colocalization was analyzed using JACoP plugin (v2.1.1). ChIP-seq data was aligned using Bowtie2 (v2.4.5) and peaks called using MACS3 (v3.0.1). Data were analyzed in deepTools2.0 and ChiPseeker (v1.32.1). CryoEM data was processed in cryoSPARC. The model was initially built in Chimera (v1.6.1) and rebuilt in Coot. Flow cytometry data was analyzed in FlowJo (v10.9).

For manuscripts utilizing custom algorithms or software that are central to the research but not yet described in published literature, software must be made available to editors and reviewers. We strongly encourage code deposition in a community repository (e.g. GitHub). See the Nature Portfolio guidelines for submitting code & software for further information.

Data

Policy information about availability of data

All manuscripts must include a data availability statement. This statement should provide the following information, where applicable:

- Accession codes, unique identifiers, or web links for publicly available datasets
- A description of any restrictions on data availability
- For clinical datasets or third party data, please ensure that the statement adheres to our policy

Raw sequencing data from the CRISPR screens, RNA-seq, and ChIP-seq experiments is deposited in SRA BioProject PRJNA1302711. Processed data from ChIP-seq is deposited in GEO GSE304976. The SP100 structure is deposited in PDB 9TNZ and EMDB EMD-56096. Numerical data and uncropped western blots are supplied in source data files. Numerical source data for Supplementary Fig. 1,3 can be found in Supplementary Data 5.

Research involving human participants, their data, or biological material

Policy information about studies with human participants or human data. See also policy information about sex, gender (identity/presentation), and sexual orientation and race, ethnicity and racism.

Reporting on sex and gender	Sex and gender were not considered in the study design nor were utilized in analysis or conclusions.
Reporting on race, ethnicity, or other socially relevant groupings	No social groupings were not considered in the study design nor were utilized in analysis or conclusions.
Population characteristics	See above.
Recruitment	Patients harnessing relevant mutations voluntary provided biological material for research use. No known biases were accounted for.
Ethics oversight	The patient cohort and associated immune cell characterization have been approved by the Ethical Commission of Northwestern and Central Switzerland (EKNZ-2015-187).

Note that full information on the approval of the study protocol must also be provided in the manuscript.

Field-specific reporting

Please select the one below that is the best fit for your research. If you are not sure, read the appropriate sections before making your selection.

☒ Life sciences ☐ Behavioural & social sciences ☐ Ecological, evolutionary & environmental sciences

For a reference copy of the document with all sections, see nature.com/documents/nr-reporting-summary-flat.pdf

Life sciences study design

All studies must disclose on these points even when the disclosure is negative.

Sample size	No sample size calculations were performed. Sample size was determined to be adequate based on the consistency of measurable differences and the standards used in the field.
Data exclusions	No data were excluded from analysis expect in the case of failed experiments due to other factors (i.e. loss of reagent activity)
Replication	All functional experiments were performed with at least three biological replicates. Each data point represents an individual biological replicate as listed in figure captions. Large data set experiments: Both screens were performed with two biological replicates and RNA-seq with three biological replicates. All replications were successful and closely aligned to each other.
Randomization	No randomization was performed. All independent biological replicates were treated identically.
Blinding	No blinding was performed as data were not subjective.

Reporting for specific materials, systems and methods

We require information from authors about some types of materials, experimental systems and methods used in many studies. Here, indicate whether each material, system or method listed is relevant to your study. If you are not sure if a list item applies to your research, read the appropriate section before selecting a response.

Materials & experimental systems

n/a	Involved in the study
☐	☒ Antibodies
☐	☒ Eukaryotic cell lines
☒	☐ Palaeontology and archaeology
☒	☐ Animals and other organisms
☒	☐ Clinical data
☒	☐ Dual use research of concern
☒	☐ Plants

Methods

n/a	Involved in the study
☐	☒ ChIP-seq
☐	☒ Flow cytometry
☒	☐ MRI-based neuroimaging

Antibodies

Antibodies used

Primary Antibody Supplier Product # Clone # Lot #
SP110 Santa Cruz Biotech sc-376741 Monoclonal B-10 K0116
SP100 Novus Biologicals NBP1-86060 Polyclonal A106767
FLAG Sigma F3165 Monoclonal M2 SLCQ9255
HA Cell Signalling Technologies 3724 Monoclonal C29F4 11
P-KAP1 (S824) Abcam ab70369 Polyclonal GR3316128-13
P-CHK1 (S345) Cell Signalling Technologies 2348 Monoclonal 133D3 4
Centromere Antibodies Inc 15-235 Polyclonal n/a
Cyclin B1 R&D Systems MAB60001 Monoclonal 2061D CKJC022109A
GAPDH (Rb) Cell Signalling Technologies 2118 Monoclonal 14C10 16
GAPDH (Ms) Cell Signalling Technologies 97166 Monoclonal D4C6R 7
SOX9 Abcam ab185966 Monoclonal EPR14335-78 GR3183675-1
53BP1 Novus Biologicals NB100-304 Polyclonal D148226
PML Santa Cruz sc-966 Monoclonal PG-M3 G1122
ZEB1 Cell Signalling Technologies 3396 Monoclonal D80D3 10
H2B Cell Signalling Technologies 12364 Monoclonal D2H6 3
Rabbit IgG isotype control Novus Biologicals NBP2-24891 Polyclonal n/a

Secondary Antibody Supplier Product # Clone # Lot #
IRDye 800CW anti-Rabbit IgG LI-COR Biosciences 926-32213 Polyclonal D30207-15
IRDye 800CW anti-Mouse IgG LI-COR Biosciences 926-32212 Polyclonal D30124-05
IRDye 680RD anti-Rabbit IgG LI-COR Biosciences 926-68073 Polyclonal D30328-05
IRDye 680RD anti-Mouse IgG LI-COR Biosciences 926-68072 Polyclonal D20503-05
Anti-rabbit Alexa Fluor Plus 405 Invitrogen A48254 Polyclonal YD369487
Anti-mouse Alexa Fluor 568 Invitrogen A11004 Polyclonal 2447869
Anti-rabbit Alexa Fluor 488 Invitrogen A11008 Polyclonal 2521157

Validation

Anti-SP110 was validated by making SP110 KO clones and comparing to WT cells (this manuscript).
Anti-SP100 was validated by making SP100 KO clones and comparing to WT cells (this manuscript). Also validated by manufacturer through independent antibody validation and orthogonal validation.
Anti-FLAG was validated through FLAG-tagged protein overexpression (this manuscript).
Anti-HA was validated through HA-tagged protein overexpression (this manuscript).
Anti-P-KAP1 has been cited over 58 times and was validated by the manufacturer for use in western blotting.
Anti-P-CHK1 has been cited over 1067 times and is approved by the manufacturer for use in western blotting.
Anti HuCentromere was tested by the manufacturer in the following protocol: Serum obtained from an autoimmune patient was purified by protein A chromatography and then tested at a series of dilutions by immunocytochemistry on ethanol-fixed Hep2 cells that were in log-phase growth. The staining pattern obtained was consistent with the pattern expected for anti-centromere staining.
Anti-cyclin B1 has been validated for use in western blot by the manufacturer.
Anti-GAPDH (Rb) has been cited for 7799 times.
Anti-GAPDH (Ms) has been cited over 440 times.
Anti-SOX9 has been cited over 175 times and is validated for use by the manufacturer in western blotting.
Anti-53BP1 has been validated by the manufacturer using genetic approaches.
Anti-PML has been cited over 696 times.
Anti-ZEB1 has been cited over 481 times and is approved for use by the manufacturer in western blotting.
Anti-H2B has been cited over 77 times and is approved for use by the manufacturer in western blotting.

Eukaryotic cell lines

Policy information about cell lines and Sex and Gender in Research

Cell line source(s)

HEK-293T ATCC
hTERT-RPE1 TP53 proficient ATCC
hTERT-RPE1 TP53 null Dr. Steve Jackson
RPE1-dCas9-BFP-KRAB(KOX1) TP53-/- Fielden, Siegner, et al. Nature, 2025
RPE1-dCas9-BFP-KRAB(KOX1) TP53-/-; SP110-/- This study
RPE1 TP53-/-; SP110-/- This study
RPE1 TP53+/+; SP110-/- This study

	RPE1 TP53-/-; SP100-/- This study<br>RPE1 TP53-/-; SP110-/-; SP100-/- This study<br>RPE1 TP53+/+; SP110-/-; SP100-/- This study<br>RPE1 TP53+/+ H2B-GFP This study<br>RPE1 TP53+/+; SP110-/- H2B-GFP This study<br>RPE1 TP53+/+; SP110-/-; SP100-/- H2B-GFP This study<br>Jurkat ATCC<br>Jurkat SP110-/- This study<br>Jurkat SP100-/- This study<br>Jurkat SP110-/-; SP100-/- This study<br>A549 Dr. Tim Clausen<br>A549 SP110-/- This study<br>A549 SP100-/- This study<br>A549 SP110-/-; SP100-/- This study<br>U2OS RNaseH1(D210N)-GFP Awwad, et al. Nat Commun 2025<br>MDCK ATCC<br>Vero-CCL81 ATCC
Authentication	STR profiling
Mycoplasma contamination	All cell lines were routinely tested for mycoplasma (MycoAlert; Lonza) and tested negative.
Commonly misidentified lines<br>(See ICLAC register)	No commonly misidentified lines were used in this study.

Plants

Seed stocks	n/a
Novel plant genotypes	n/a
Authentication	n/a

ChIP-seq

Data deposition

☒ Confirm that both raw and final processed data have been deposited in a public database such as GEO.

☒ Confirm that you have deposited or provided access to graph files (e.g. BED files) for the called peaks.

Data access links<br>*May remain private before publication.*	Raw sequencing data is deposited in SRA BioProject PRJNA1302711. Aligned data and called peak data is deposited in GEO GSE304976.
Files in database submission	Anti-SP100 in wildtype genotype<br>Anti-SP100 in SP110 KO genotype<br>Anti-IgG in wildtype genotype<br>Anti-IgG in SP110 KO genotype
Genome browser session<br>(e.g. UCSC)	*Provide a link to an anonymized genome browser session for "Initial submission" and "Revised version" documents only, to enable peer review. Write "no longer applicable" for "Final submission" documents.*

Methodology

Replicates	Two independent biological replicates for each condition were performed and generally agreed with each other.
Sequencing depth	The target read depth was 25 million reads per sample on a 150 bp paired-end Illumina NovaSeq 6000. Overall 90% of reads mapped.
Antibodies	SP100 Novus Biologicals NBP1-86060 Polyclonal lot # A106767<br>Rabbit IgG isotype control Novus Biologicals NBP2-24891 Polyclonal lot # n/a
Peak calling parameters	Read mapping to hg19 was performed using BowTie2 and peaks called using MACS3 comparing the anti-SP100 pulldown sample to the match genotype IgG pulldown sample.
Data quality	Anti-SP100 WT: 21,049 peaks above fold enrichment of 5 with p-value <0.05.<br>Anti-SP100 SP110 KO: 4,850 peaks above fold enrichment of 5 with p-value <0.05.

Software	Data was analyzed and visualized using deepTools2 and ChIPseeker.

Flow Cytometry

Plots

Confirm that:

☒ The axis labels state the marker and fluorochrome used (e.g. CD4-FITC).

☒ The axis scales are clearly visible. Include numbers along axes only for bottom left plot of group (a 'group' is an analysis of identical markers).

☒ All plots are contour plots with outliers or pseudocolor plots.

☒ A numerical value for number of cells or percentage (with statistics) is provided.

Methodology

Sample preparation	Cells were pelleted, washed 1x with PBS, resuspended in a PBS / 10 % FBS solution, and analyzed immediately.
Instrument	Attune NxT Flow Cytometer (with autosampler), Thermo Fisher
Software	Flow cytometry data was collected using Attune Nxt Software v3.2.1 and analyzed with FlowJo v10.9.
Cell population abundance	For each sample, 10,000 events (gated on single cells) were collected.
Gating strategy	Cells were first gated for live cells (SSC-A vs FSC-A), then gated for single cells (FSC-H vs FSC-A). XFP (GFP, mCherry, or BFP) positive cells were then gated on SSC-A vs XFP using a XFP negative cell line to set the gate.

☒ Tick this box to confirm that a figure exemplifying the gating strategy is provided in the Supplementary Information.
